# Discovery of Furanoquinone Derivatives as a Novel Class of DNA Polymerase and Gyrase Inhibitors for MRSA Eradication in Cutaneous Infection

**DOI:** 10.3389/fmicb.2019.01197

**Published:** 2019-05-29

**Authors:** Shih-Chun Yang, Kai-Wei Tang, Chih-Hung Lin, Ahmed Alalaiwe, Chih-Hua Tseng, Jia-You Fang

**Affiliations:** ^1^Department of Cosmetic Science, Providence University, Taichung, Taiwan; ^2^School of Pharmacy, College of Pharmacy, Kaohsiung Medical University, Kaohsiung, Taiwan; ^3^Center for General Education, Chang Gung University of Science and Technology, Taoyuan, Taiwan; ^4^Department of Pharmaceutics, College of Pharmacy, Prince Sattam Bin Abdulaziz University, Al Kharj, Saudi Arabia; ^5^Department of Fragrance and Cosmetic Science, College of Pharmacy, Kaohsiung Medical University, Kaohsiung, Taiwan; ^6^Department of Medical Research, Kaohsiung Medical University Hospital, Kaohsiung, Taiwan; ^7^Department of Pharmacy, Kaohsiung Municipal Ta-Tung Hospital, Kaohsiung, Taiwan; ^8^Pharmaceutics Laboratory, Graduate Institute of Natural Products, Chang Gung University, Taoyuan, Taiwan; ^9^Chinese Herbal Medicine Research Team, Healthy Aging Research Center, Chang Gung University, Taoyuan, Taiwan; ^10^Research Center for Food and Cosmetic Safety and Research Center for Chinese Herbal Medicine, Chang Gung University of Science and Technology, Taoyuan, Taiwan; ^11^Department of Anesthesiology, Chang Gung Memorial Hospital, Taoyuan, Taiwan

**Keywords:** MRSA, furanoquinone, skin, DNA polymerase, gyrase

## Abstract

Methicillin-resistant *Staphylococcus aureus* (MRSA) is the primary microbe responsible for skin infections that are particularly difficult to eradicate. This study sought to inhibit planktonic and biofilm MRSA using furanoquinone-derived compounds containing imine moiety. A total of 19 furanoquinone analogs were designed, synthesized, and assessed for anti-MRSA potency. Among 19 compounds, (Z)-4-(hydroxyimino)naphtho[1,2-*b*]furan-5(4*H*)-one (HNF) and (Z)-4-(acetoxyimino)naphtho[1,2-*b*]furan-5(4*H*)-one (ANF) showed antibacterial activity superior to the others based on an agar diffusion assay. HNF and ANF exerted a bactericidal effect with a minimum inhibitory concentration (MIC) of 9.7 ∼ 19.5 and 2.4 ∼ 9.7 μg/ml, respectively. Both compounds were able to reduce the MRSA count by 1,000-fold in biofilm as compared to the control. *In vivo* efficacy was evaluated using a mouse model of skin infection. Topical application of lead compounds significantly suppressed abscess occurrence and the MRSA burden, and also ameliorated the skin-barrier function. The biochemical assay indicated the compounds’ inhibition of DNA polymerase and gyrase. *In silico* docking revealed a favorable interaction of the compounds with DNA polymerase and gyrase although the binding was not very strong. The total DNA analysis and proteomic data suggested a greater impairment of some proteins by HNF than ANF. In general, HNF and ANF were similarly potent in MRSA inhibition *in vitro* and *in vivo*. The findings demonstrated that there was room for structural modification of furanoquinone compounds that could be used to identify anti-MRSA agent candidates.

## Introduction

*Staphylococcus aureus* is largely involved in hospital- and community-acquired infections. *S. aureus* has become resistant to conventional antibiotics due to its resilient ability to develop several approaches to fight against the antibiotics. More than 2 million people are infected by superbug pathogens, which cause >700,000 deaths each year (Fair and Tor, 2014). The most remarkable strain among the antibiotic-resistant bacteria is methicillin-resistant *S. aureus* (MRSA). More than 50% of clinical isolates from *S. aureus* show methicillin resistance (Hiramatsu et al., 2014). About 75% of MRSA causes infection in the skin and soft tissues (Kurosu et al., 2013). MRSA facilely locates in the lesions of atopic dermatitis and chronic wounds, playing a critical role in disease progression (Shi et al., 2018). As a resident microbe in skin appendages, MRSA is also responsible for causing folliculitis and hidradenitis suppurativa. The development of new agents for eradicating cutaneous drug-resistant *S. aureus* is urgently needed.

Previously, some compounds belonging to the quinine family were reported to show inhibitory activity against MRSA (Nagata et al., 1998; Rejiniemon et al., 2014; May Zin et al., 2017). We recently demonstrated the anti-MRSA capability of two furanoquinones, naphtho[1,2-*b*]furan-4,5-dione and naphtho[2,3-*b*]furan-4,9-dione (Yang et al., 2017). In this study, we designed alternatives for imine in the 4-position of both compounds in the search for derivatives with improved MRSA inhibition. The aim of the present work was to assess furanoquinone analogs for their *in vitro* killing of drug-resistant *S. aureus* and their *in vivo* efficacy in fighting skin infection. Bacteria generate extracellular polymeric substances to form biofilm, which is innately resistant to conventional antibiotics. MRSA is recognized as the frequent cause of biofilm (Vergara et al., 2017). The morbidity and mortality of patients with cutaneous wounds increase once the MRSA biofilm colonizes in the open lesion (Song et al., 2016). In addition to the planktonic form of MRSA, we also examined the anti-biofilm activity of the furanoquinone derivatives.

The antibacterial effect of new drugs or lead compounds is associated with a variety of targets such as ribosomes, polymerases, and topoisomerases (Brown and Wright, 2016). The anti-MRSA mechanisms of the furanoquinone derivatives with potential activity were investigated by using the experimental platforms of wrapping assay and proteomics. The biocidal action was further validated by docking simulation using the crystallographic structure of the target proteins in complex with the lead compounds to elucidate the binding modes at enzymes’ active sites.

## Materials and Methods

### Synthetic Procedures

Commercial reagents were used as received without additional purification. Melting points were determined with the Electrothermal IA9100 micro-melting point apparatus and were uncorrected. NMR spectra were recorded with a Varian Unity-400 MHz spectrometer using DMSO-*d*_6_ and CDCl_3_ as solvent and tetramethylsilane as the internal standard. Chemical shifts were expressed as δ (ppm). Splitting patterns had been described as follows: s = singlet; brs = broad singlet; d = doublet; t = triplet; dt = double triplet; m = multiplet. The raw data of NMR for all compounds are shown in Supplementary Materials. Analytical TLC was performed on Art. 5554 Kieselgel 60 GF254 produced by Merck, and the compound spots were detected with a UV light indicator irradiated at 254 and 366 nm. Art. 7734 Kieselgel 60 GF254 (70–400 mesh) made by Merck was used for column chromatography. The purity of the compounds was determined with elemental analysis (EA). EA was recorded on an Heraeus CHN-O Rapid apparatus and the results were within ±0.4% of the theoretical value.

#### General Procedure for the Synthesis of Compounds 3a-3c

The protocol for synthesizing compounds 3a, 3b, and 3c was described in our previous study (Tseng et al., 2010).

#### General Procedure for the Synthesis of Compounds 4a-4c

To a suspension of 2 (0.20 g, 1.0 mmol) in ethanol (30 ml) was added appropriate hydroxylamines hydrochloride (3.0 mmol), and the mixture was refluxed for 1 h. The solvent was removed in vacuum and the residue suspended in H_2_O (20 ml). The resulting precipitate was purified by methanol:CH_2_Cl_2_ (1:50) and recrystallized from ethanol to give the title products.

### (*Z*)-4-(Hydroxyimino)Naphtho[1,2-*b*]Furan-5(4*H*)-One (4a)

Yield 67%. Mp.: 233–234°C. ^1^H NMR (400 MHz, DMSO-*d*_6_): 8.69 (brs, 1H, -OH), 8.06–8.04 (m, 1H, 9-H), 7.96 (d, *J* = 2.0 Hz, 1H, 2-H), 7.80-7.75 (m, 2H, 6-H, 8-H), 7.53-7.49 (m, 1H, 7-H), 7.26 (d, *J* = 2.0 Hz, 1H, 3-H). ^13^C NMR (100 MHz, DMSO-*d*_6_): 181.15, 148.98, 144.85, 143.28, 134.90, 128.84, 128.68, 128.48, 128.20, 120.75, 113.35, 112.71. Anal. calcd for C_12_H_7_NO_3_ : C 67.61, H 3.31, N 6.57; found: C 67.47, H 3.36, N 6.53.

### (Z)-4-(Methoxyimino)Naphtho[1,2-*b*]Furan-5(4*H*)-One (4b)

Yield 71%. Mp.: 143–144°C. ^1^H NMR (400 MHz, CDCl_3_): 8.19–8.17 (m, 1H, 9-H), 7.72–7.70 (m, 1H, 6-H), 7.62 (dt, *J* = 1.2, 7.6 Hz, 1H, 8-H), 7.50 (d, *J* = 2.0 Hz, 1H, 2-H), 7.42–7.37 (m, 1H, 7-H), 7.11 (d, *J* = 2.0 Hz, 1H, 3-H), 4.31 (s, 3H, CH_3_). ^13^C NMR (100 MHz, CDCl_3_): 181.86, 150.86, 143.55, 143.08, 134.55, 129.22, 129.11, 129.05, 128.42, 120.02, 113.47, 112.87, 64.50. Anal. calcd for C_13_H_9_NO_3_ : C 68.72, H 3.99, N 6.16; found: C 68.65, H 4.02, N 6.12.

### (*Z*)-4-[(Benzyloxy)Imino]Naphtho[1,2-*b*]Furan-5(4*H*)-One (4c)

Yield 59%. Mp.: 146–147°C. ^1^H NMR (400 MHz, CDCl_3_): 8.19–8.17 (m, 1H, 9-H), 7.71–7.69 (m, 1H, 6-H), 7.62 (dt, *J* = 1.2, 7.6 Hz, 1H, 8-H), 7.47–7.34 (m, 7H, 2-H, 7-H, Ar-H), 7.04 (d, *J* = 1.6 Hz, 1H, 3-H), 5.55 (s, 2H, CH_2_). ^13^C NMR (100 MHz, CDCl_3_): 181.92, 150.88, 143.48, 143.38, 136.13, 134.57, 129.20, 129.10, 129.05, 128.60 (2C), 128.48, 128.45 (2C), 124.03, 120.03, 113.53, 113.07, 79.18. Anal. calcd for C_19_H_13_NO_3_ : C 75.24, H 4.32, N 4.62; found: C 75.03, H 4.37, N 4.43.

#### General Procedure for the Synthesis of Compounds 5e-5h

To a suspension of 1 (0.20 g, 1.0 mmol) in ethanol (30 ml) was added appropriate hydrazines (3.0 mmol), and the mixture was refluxed for 4 h. The solvent was removed in vacuum and the residue suspended in H_2_O (20 ml). The resulting precipitate was purified by methanol:CH_2_Cl_2_ (1:50) and recrystallized from ethanol to give the title products.

### (*Z*)-4-(2-Phenylhydrazono)Naphtho[2,3-*b*]Furan-9(4*H*)-One (5e)

Yield 43%. Mp.: 237–238°C.^1^H NMR (400 MHz, DMSO-*d*_6_): 10.58 (s, 1H, NH), 8.60–8.58 (m, 1H, 8-H), 8.42 (d, *J* = 2.0 Hz, 1H, 2-H), 8.20–8.18 (m, 1H, 5-H), 7.94 (d, *J* = 2.0 Hz, 1H, 3-H), 7.78–7.74 (m, 1H, 6-H), 7.64–7.57 (m, 3H, 7-H, Ar-H), 7.43–7.40 (m, 2H, Ar-H), 7.08–7.05 (m, 1H, Ar-H). ^13^C NMR (100 MHz, DMSO-*d*_6_): 171.35, 149.38, 147.25, 144.41, 136.13, 132.12, 129.99, 129.18 (2C), 127.74, 127.61, 125.43, 123.98, 122.76, 122.46, 115.13 (2C), 110.44. Anal. calcd for C_18_H_12_N_2_O_2_ ⋅ 0.1H_2_O : C 74.51, H 4.25, N 9.66; found: C 74.27, H 4.23, N 9.57.

### (*Z*)-4-(2-(4-Fluorophenyl)Hydrazono)Naphtho[2,3-*b*]Furan-9(4*H*)-One (5f)

Yield 47%. Mp.: 222–223°C. ^1^H NMR (400 MHz, DMSO-*d*_6_): 10.57 (s, 1H, NH), 8.58–8.56 (m, 1H, 8-H), 8.43 (d, *J* = 2.0 Hz, 1H, 2-H), 8.20–8.18 (m, 1H, 5-H), 7.93 (d, *J* = 2.0 Hz, 1H, 3-H), 7.77–7.73 (m, 1H, 6-H), 7.65–7.57 (m, 3H, 7-H, Ar-H), 7.28–7.24 (m, 2H, Ar-H). ^13^C NMR (100 MHz, DMSO-*d*_6_): 171.35, 158.07 (^1^*J*_CF_ = 237.3 Hz), 149.39, 147.23, 141.05 (^4^*J*_CF_ = 1.5 Hz), 136.07, 132.08, 130.00, 127.83, 127.68, 125.43, 123.98, 122.73, 116.59 (2C, ^3^*J*_CF_ = 8.3 Hz), 115.79 (2C, ^2^*J*_CF_ = 22.0 Hz), 110.42. Anal. calcd for C_18_H_11_FN_2_O_2_ : C 70.58, H 3.62, N 9.15; found: C 70.29, H 3.50, N 9.15.

### (*Z*)-4-(2-(4-Methoxyphenyl)Hydrazono)Naphtho[2,3-*b*]Furan-9(4*H*)-One (5g)

Yield 51%. Mp.: 178–179°C. ^1^H NMR (400 MHz, DMSO-*d*_6_): 10.54 (s, 1H, NH), 8.58–8.56 (m, 1H, 8-H), 8.46 (d, *J* = 2.0 Hz, 1H, 2-H), 8.21–8.18 (m, 1H, 5-H), 7.94 (d, *J* = 2.0 Hz, 1H, 3-H), 7.76–7.72 (m, 1H, 6-H), 7.61–7.55 (m, 3H, 7-H, Ar-H), 7.03–7.00 (m, 2H, Ar-H), 3.78 (s, 3H, OCH_3_). ^13^C NMR (100 MHz, DMSO-*d*_6_): 171.13, 155.32, 149.37, 147.02, 138.06, 136.24, 131.91, 129.86, 127.35, 126.24, 125.38, 123.81, 122.71, 116.47 (2C), 114.54 (2C), 110.21, 55.33. Anal. calcd for C_19_H_14_N_2_O_3_ : C 71.69, H 4.43, N 8.80; found: C 71.29, H 4.39, N 8.76.

### (*Z*)-4-(2-(p-Tolyl)Hydrazono)Naphtho[2,3-*b*]Furan-9(4*H*)-One (5h)

Yield 49%. Mp.: 212–213°C. ^1^H NMR (400 MHz, DMSO-*d*_6_): 10.54 (s, 1H, NH), 8.59–8.57 (m, 1H, 8-H), 8.42 (d, *J* = 2.0 Hz, 1H, 2-H), 8.20–8.18 (m, 1H, 5-H), 7.93 (d, *J* = 2.0 Hz, 1H, 3-H), 7.77–7.73 (m, 1H, 6-H), 7.60–7.56 (m, 1H, 7-H), 7.54–7.52(m, 2H, Ar-H), 7.23–7.21 (m, 2H, Ar-H), 2.31 (s, 3H, CH_3_). ^13^C NMR (100 MHz, DMSO-*d*_6_): 171.25, 149.36, 147.15, 142.10, 136.20, 132.01, 131.53, 129.93 (2C), 129.62, 127.55, 126.86, 125.40, 123.90, 122.73, 115.15 (2C), 110.32, 20.44. Anal. calcd for C_19_H_14_N_2_O_2_ ⋅ 0.15H_2_O : C 75.02, H 4.72, N 9.21; found: C 74.83, H 4.50, N 9.15.

#### General Procedure for the Synthesis of Compounds 6d-6h

To a suspension of 2 (0.20 g, 1.0 mmol) in ethanol (30 ml) was added appropriate hydrazines (3.0 mmol), and the mixture was refluxed for 2 h. The solvent was removed in vacuum and the residue suspended in H_2_O (20 ml). The resulting precipitate was purified by methanol:CH_2_Cl_2_ (1:50) and recrystallized from ethanol to give the title products.

### (*Z*)-*N*′-(5-Oxonaphtho[1,2-*b*]Furan-4(5*H*)-Ylidene)Acetohydrazide (6d)

Yield 65%. Mp.: 188–189°C. ^1^H NMR (400 MHz, DMSO-*d*_6_): 13.66 (brs, 1H, NH), 8.08–8.06 (m, 1H, 9-H), 7.93–7.92 (m, 1H, 2-H), 7.80–7.76 (m, 1H, 8-H), 7.72–7.70 (m, 1H, 6-H), 7.49–7.45 (m, 1H, 7-H), 6.90–6.89 (m, 1H, 3-H), 2.36 (s, 3H, CH_3_). ^13^C NMR (100 MHz, DMSO-*d*_6_): 181.72, 148.18, 145.96, 135.56, 129.03, 128.45, 128.20, 127.69, 120.38, 120.00, 107.36, 19.56. Anal. calcd for C_14_H_10_N_2_O_3_ : C 66.14, H 3.96, N 11.02; found: C 66.22, H 4.02, N 10.91.

### (*Z*)-2-(5-Oxonaphtho[1,2-*b*]Furan-4(5*H*)-Ylidene)Hydrazine-1-Carboxamide (6e)

Yield 55%. Mp.: 221–222°C. ^1^H NMR (400 MHz, DMSO-*d*_6_): 13.23 (brs, 1H, NH), 8.17–8.15 (m, 1H, 9-H), 7.94 (d, *J* = 2.0 Hz, 1H, 2-H), 7.84–7.76 (m, 2H, 6-H, 8-H), 7.52–7.47 (m, 1H, 7-H), 7.36 (brs, 2H, NH_2_), 7.28 (d, *J* = 2.0 Hz, 1H, 3-H ). ^13^C NMR (100 MHz, DMSO-*d*_6_): 181.20, 155.15, 146.96, 145.53, 135.09, 128.72, 128.48, 128.19, 127.20, 120.54, 120.17, 107.87. Anal. calcd for C_13_H_9_N_3_O_3_ ⋅ 0.1H_2_O : C 60.73, H 3.61, N 16.35; found: C 60.71, H 3.53, N 16.18.

### (*Z*)-2-(5-Oxonaphtho[1,2-*b*]Furan-4(5*H*)-Ylidene)Hydrazine-1-Carbothioamide (6f)

Yield 53%. Mp.: 219–220°C. ^1^H NMR (400 MHz, DMSO-*d*_6_): 13.84 (brs, 1H, NH), 9.33 (brs, 1H, NH_2_), 9.00 (brs, 1H, NH_2_), 8.17–8.15 (m, 1H, 9-H), 7.94 (d, *J* = 2.0 Hz, 1H, 2-H), 7.83–7.76 (m, 2H, 6-H, 8-H), 7.53–7.49 (m, 1H, 7-H), 7.35 (d, *J* = 2.0 Hz, 1H, 3-H ). ^13^C NMR (100 MHz, DMSO-*d*_6_): 181.57, 179.26, 148.24, 145.60, 135.34, 128.93, 128.44, 128.26, 127.52, 120.33, 120.29, 108.12. Anal. calcd for C_13_H_9_N_3_O_2_S : C 57.55, H 3.34, N 15.49; found: C 57.72, H 3.45, N 15.15.

### (*Z*)-*N*-Methyl-2-(5-Oxonaphtho[1,2-*b*]Furan-4(5*H*)-Ylidene)Hydrazine-1-Carbothioamide (6g)

Yield 58%. Mp.: 218–219°C. ^1^H NMR (400 MHz, DMSO-*d*_6_): 14.00 (brs, 1H, NH), 9.51–9.48 (m, 1H, NHCH_3_), 8.17–8.15 (m, 1H, 9-H), 7.95 (d, *J* = 2.0 Hz, 1H, 2-H), 7.83–7.76 (m, 2H, 6-H, 8-H), 7.53–7.48 (m, 1H, 7-H), 7.28 (d, *J* = 2.0 Hz, 1H, 3-H ), 3.14 (d, *J* = 4.8 Hz, 3H, NHCH_3_). ^13^C NMR (100 MHz, DMSO-*d*_6_): 181.35, 177.91, 148.08, 145.62, 135.28, 128.87, 128.37, 128.23, 128.10, 127.48, 120.29, 120.20, 107.91, 31.56. Anal. calcd for C_14_H_11_N_3_O_2_S ⋅ 0.5H_2_O : C 57.12, H 4.11, N 14.27; found: C 57.43, H 3.95, N 13.94.

### (*Z*)-4-(2-Phenylhydrazono)Naphtho[1,2-*b*]Furan-5(4*H*)-One (6h)

Yield 65%. Mp.: 159–160°C. ^1^H NMR (400 MHz, CDCl_3_): 15.70 (brs, 1H, NH), 8.37–8.35 (m, 1H, 9-H), 7.87–7.85 (m, 1H, 6-H), 7.68–7.64 (m, 1H, 8-H), 7.58 (d, *J* = 2.0 Hz, 1H, 2-H), 7.56–7.54 (m, 2H, Ar-H), 7.44–7.39 (m, 3H, Ar-H), 7.19–7.15 (m, 1H, 7-H), 6.99 (d, *J* = 2.0 Hz, 1H, 3-H). ^13^C NMR (100 MHz, CDCl_3_): 178.82, 145.87, 144.17, 142.39, 133.25, 129.55, 128.90, 128.58, 128.22 (2C), 127.78, 126.00 (2C), 125.20, 121.22, 120.22, 116.24, 106.70. Anal. calcd for C_18_H_12_N_2_O_2_ ⋅ 0.1H_2_O : C 74.51, H 4.25, N 9.66; found: C 74.28, H 4.23, N 9.53.

#### General Procedure for the Synthesis of Compounds 7a, 7b, 8a, and 8b

The synthetic protocol of compound 7a was described in the previous study (Tseng et al., 2010). To a suspension of 3a or 4a (0.21 g, 1.0 mmol) in acetic anhydride (3 ml) or methanesulfonyl chloride (3 ml) was added pyridine (1.0 ml). The reaction mixture was stirred at room temperature for 1 h. The solvent was removed in vacuum, and the residue was triturated with H_2_O (20 ml), filtered, and washed with H_2_O. The crude product was recrystallized from ethanol to give the title product.

### (*Z*)-4-{[(Methylsulfonyl)Oxy]Imino}Naphtho[2,3-*b*]Furan-9(4*H*)-One (7b)

Yield 78%. Mp.: 226–227°C. ^1^H NMR (400 MHz, CDCl_3_): 8.40–8.37 (m, 1H, 8-H), 8.34–8.30 (m, 1H, 5-H), 7.84 (d, *J* = 2.0 Hz, 1H, 2-H), 7.73–7.68 (m, 2H, 6-H, 7-H), 7.42 (d, *J* = 2.0 Hz, 1H, 3-H), 3.37 (s, 3H, CH_3_). ^13^C NMR (100 MHz, CDCl_3_) δ 172.50, 149.76, 148.67, 148.59, 148.24, 133.29, 131.90, 131.42, 130.83, 127.35, 124.97, 113.59, 36.87. Anal. calcd for C_13_H_9_NO_5_S : C 53.60, H 3.11, N 4.81; found: C 53.49, H 3.11, N 4.88.

### (*Z*)-4-(Acetoxyimino)Naphtho[1,2-*b*]Furan-5(4*H*)-One (8a)

Yield 82%. Mp.: 170–171°C. ^1^H NMR (400 MHz, DMSO-*d*_6_): 8.03 (d, *J* = 2.0 Hz, 1H, 2-H), 8.02–8.00 (m, 1H, 9-H), 7.81–7.74 (m, 2H, 6-H, 8-H), 7.56–7.52 (m, 1H, 7-H), 7.26 (d, *J* = 2.0 Hz, 1H, 3-H ), 2.40 (s, 3H, CH_3_). ^13^C NMR (100 MHz, DMSO-*d*_6_): 180.99, 167.73, 152.31, 146.35, 145.52, 135.48, 129.55, 128.49, 128.34, 128.05, 121.33, 113.08, 112.72, 19.46. Anal. calcd for C_14_H_9_NO_4_ : C 65.88, H 3.55, N 5.49; found: C 65.45, H 3.64, N 5.44.

### (*Z*)-4-{[(Methylsulfonyl)Oxy]Imino}Naphtho[1,2-*b*]Furan-5(4*H*)-One (8b)

Yield 84%. Mp.: 196–197°C. ^1^H NMR (400 MHz, DMSO-*d*_6_): 8.05 (d, *J* = 2.0 Hz, 1H, 2-H), 8.02–8.00 (m, 1H, 9-H), 7.83–7.75 (m, 2H, 6-H, 8-H), 7.58–7.54 (m, 1H, 7-H), 7.07 (d, *J* = 2.0 Hz, 1H, 3-H), 3.50 (s, 3H, CH_3_). ^13^C NMR (100 MHz, DMSO-*d*_6_): 180.01, 153.24, 147.52, 145.79, 135.64, 129.88, 128.59, 128.31, 127.81, 121.54, 112.64, 112.02, 36.77. Anal. calcd for C_13_H_9_NO_5_ S : C 53.60, H 3.11, N 4.81; found: C 53.21, H 3.29, N 4.86.

### Bacterial Strains

MRSA (ATCC 33591) and *Escherichia coli* (ATCC 8739) were supplied by American Type Culture Collection. The clinical isolates of MRSA (KM-1 and KM-5) were isolated from Kaohsiung Medical University Hospital. KV-1 and KV-5 were vancomycin-intermediate *S. aureus* (VISA) strains found in Kaohsiung Medical University Hospital provided by Dr. Po-Liang Lu. The clinical isolates were taken from infected patients using a protocol approved by the institutional review board at Kaohsiung Medical University Hospital. The patients provided written informed consent to participate. These strains were cultured in tryptic soy broth (TSB) at 37°C with 150 rpm.

### Agar Diffusion Test

We began the antibacterial study with an agar diffusion assay. MRSA (OD_600_ = 0.7) was inoculated in TSB agar (0.75%). The MRSA-agar mixture (5 ml) was distributed in the dish for 15 min, followed by the dropping of lead compounds (10 μl) onto the agar. DMSO was used as the vehicle of the compounds. The inhibition-zone diameter was measured after a 16-h treatment. The control group was the MRSA-agar mixture added with DMSO alone.

### Minimum Inhibitory Concentration (MIC) and Minimum Bactericidal Concentration (MBC)

A twofold serial dilution method was employed to detect MIC. An overnight bacteria culture was diluted in TSB to achieve OD_600_ = 0.01 (about 2 × 10^6^ CFU/ml). The detailed procedure for examining MIC and MBC was described previously (Yang et al., 2017).

### Live/Dead MRSA Assay

The viability and death of MRSA by treatment of compounds (10, 50, and 100 μg/ml) for 4 h were stained with a Live/Dead BacLight^®^ (Molecular Probes). The kit was incubated with bacteria for 15 min. The death rate was then determined by flow cytometry (Accuri C6, BD Biosciences). The image of live/dead MRSA distribution was visualized using Leica DMi8 fluorescence microscopy.

### Biofilm Detection

A Cellview^®^ dish was used by inoculating the microbes (OD_600_ = 0.1) in TSB with 1% glucose at 37°C for 24 h to form biofilm. The biofilm was treated by furanoquinone derivatives at 25, 50, or 100 μg/ml or cetylpyridinium chloride (CPC) at 100 μg/ml for 24 h. PBS was utilized to rinse the biofilm for removing the loosely adherent planktonic bacteria and suspended in PBS. The biofilm structure in PBS was broken by strong vortex for recovering MRSA inside the biofilm. The recovered MRSA outside and inside the biofilm were serially diluted and plated in an agar plate for 24 h in order to count CFU. The biofilm, which was treated with 100 μg/ml compounds, was stained by BacLight^®^ for 15 min for confocal microscopic observation. The 3D structure and thickness of the biofilm were evaluated by Leica confocal microscopy (TSC SP2).

### Cutaneous MRSA Infection

A female BALB/c mouse (8 weeks old) was purchased from the National Laboratory Animal Center (Taipei, Taiwan). This study was carried out in accordance with the principles of the Basel Declaration and recommendations of Guidelines for the Care and Use of Laboratory Animals of Chang Gung University. The protocol was approved by the Chang Gung University. After shaving the mouse’s back hair, MRSA (1 × 10^6^ CFU) in PBS (150 μl) was subcutaneously injected into the dorsal region. The topical lead compounds (100 μg/ml) with a volume of 0.1 ml were applied onto the injection site every 24 h for 6 days. The appearance of the skin surface was monitored using a Mini Scope-V digital magnifier (M&T Optics). Transepidermal water loss (TEWL) was assessed using Tewameter (TM300, Courage and Khazaka) from 0 to 6 days after MRSA injection. The yellow–brown color (b^∗^) of the skin was quantified by CD100 colorimeter (Yokogawa). The skin was excised for homogenization by MagNA Lyser (Roche) at the end of the experiment. CFU of bacteria within the skin was detected by serially diluted skin homogenates on TSB.

### Cutaneous Tolerance

The 10% DMSO/PBS containing 100 μg/ml lead compounds was topically applied daily (0.1 ml) on the mouse’s back for 5 consecutive days. The compound-containing solution was replaced with a new one each day. After removal, the treated skin area was determined by TEWL, erythema (a^∗^), and cutaneous surface pH value.

### Morphology of MRSA

MRSA morphology was observed by scanning electron microscopy (SEM). The bacteria at OD_600_ = 0.1 were treated with furanoquinone compounds or CPC at 100 μg/ml for 24 h. MRSA was then fixed with 3% glutaraldehyde and 2% paraformaldehyde in cacodylate buffer several times. After dehydration in an ascending series of ethanol, the samples were coated with gold and visualized under Hitachi SU8220 SEM.

### Total DNA in MRSA

The bacteria were grown in TSB to OD_600_ = 3 and then treated with furanoquinone compounds (200 μg/ml) at 37°C for 4 h. The centrifuged pellet was resuspended in water. The analysis of total DNA was carried out using a Presto Mini Bacteria Kit based on the manufacturer’s instructions (Yang et al., 2017).

### Total RNA in MRSA

The MRSA culture and compound treatment protocol for total RNA quantification was the same with total DNA measurement. The RNA in MRSA was extracted using a Direct-zol RNA Miniprep kit (Zymo) based upon the manufacturer’s instructions. The total RNA concentration was analyzed using a UV/visible spectrophotometer at 260 nm.

### Total Protein in MRSA

The MRSA culture and subsequent treatment for the protein concentration measurement was the same with the total DNA measurement. The total protein content was analyzed using a Bio-Rad protein assay kit in an ELISA reader at 595 nm.

### Anti-Taq DNA Polymerase Activity

The polymerase chain reaction (PCR) was performed with a 50-μl volume at the following concentrations: 100 ng MRSA genomic DNA as the template, 0.4 μM primer 1 (isaB-F: 5′-atgaataaaaccagtaaagtttg-3′), 0.4 μM primer 2 (isaB-R: 5′-ttatttacttgttttgtatgg-3′), 1x YEA Taq PCR reaction buffer, 2.5 mM dNTP, 2.5 U YEA Taq DNA polymerase, and lead compounds (0.1 ∼ 2 μg/μl). The PCR reaction was done with the instrument at 95°C for 1 min to complete 30 cycles of 10 s at 95°C and 30 s at 56°C to generate 528 bp amplicons and analyzed by agar electrophoresis.

### Wrapping Assay

The relaxed form of Pbr322 DNA was prepared by incubating supercoiled pBR322 DNA with topoisomerase I according to the manufacturer’s instructions. The relaxed DNA was extracted with a Gel/PCR DNA Fragments Extraction kit and resuspended in buffer. For the gyrase supercoiling reaction, the reaction mixture (20 μl) of 100 ng relaxed from DNA, gyrase reaction buffer, 2 U gyrase, and lead compounds (0.1 ∼ 2 μg/μl) was incubated at 37°C for 1 h. The reaction was terminated by increasing the temperature to 65°C for 20 min. The DNA products were analyzed by electrophoresis on the agarose gel.

### Proteomic Characterization

The treatment procedure of the lead compounds was the same as for the total DNA quantification. The SDS-PAGE was carried out followed by Coomassie blue staining. The bands were taken and digested with trypsin for 12 h. Trichloroacetic acid (0.5%) was employed to acidify the digested proteins. The MALDI-mass analysis was carried out on a Bruker Ultraflex MALDI-TOF mass spectrometer. The spectra were collected from 400 shots per spectrum over an m/z range of 600 ∼ 3000, and calibrated by 4-point internal calibration. The fold change of the protein expression after lead compound treatment was estimated by band quantification of Prodigy Samespots analysis software. The protein masses were assigned and used for a database search with the MASCOT search engine^1^. The detailed information of mass/mass analysis was the same as previous study (Pan et al., 2012).

### *In silico* Docking

The crystal structures of the proteins were downloaded from the RCSB Protein Data Bank. The 3D conformation of the compounds was generated by ChemBio 3D Ultra 14.0. The molecular docking was conducted using an Achilles Blind Docking Server^2^. The “blind docking” approach was the setup for docking of the small molecule to the targets (Sánchez-Linares et al., 2012). Visual representation of molecules was created with 3Dmol described by Rego and Koes (2015).

### Statistical Analysis

The data presented as mean and standard deviation (SD). The difference in the data of the experimental groups was analyzed using the Kruskal–Wallis test. The *post hoc* test for checking the individual difference was Dunn’s test. The significance was demonstrated as ^∗^ for *p* < 0.05, ^∗∗^ for *p* < 0.01, and ^∗∗∗^ for *p* < 0.001 in the figures.

## Results

### Inhibitory Activity Screening Against Drug-Resistant Bacteria

We herein synthesized imine-containing furanoquinone derivatives from their alkoxime and hydrazino precursors in the regiospecific and stereospecific modes. Schemes 1, 2 describe the synthetic routes to the furanoquinone derivatives. Both schemes are illustrated in Supplementary Materials. The physicochemical properties including Alog *P*, hydrogen-bonding donor number (HBD), hydrogen-bonding acceptor number (HBA), and molecular weight (MW) are estimated by molecular modeling (Discovery Studio 3.1, Accelrys) as shown in Table 1. We first tested the anti-MRSA activity of compounds 3a through 8b by agar diffusion assay. The initial antibacterial screening showed no inhibition zone against MRSA by all linear furanoquinones (3, 5, and 7 series) as demonstrated in Table 1. Some angular furanoquinones (4, 6, and 8 series) were found to effectively inhibit MRSA growth. The angular compounds with an alkoxime linker (4 series) displayed stronger inhibition than those with a hydrazino linker (6 series). Compounds 4a ((Z)-4-(hydroxyimino)naphtho[1,2-*b*]furan-5(4*H*)-one, HNF) and 8a ((Z)-4-(acetoxyimino)naphtho[1,2-*b*]furan-5(4*H*)-one, ANF) provided the largest inhibition zone with a diameter of 23.5 and 24.4 mm, respectively. Most of the compounds with a hydrazino linker were inactive, except for compounds 6g and 6h (TNF), which had the moderate inhibition zone of 15.3 and 9.1 mm.HNF, ANF, and TNF were selected for further evaluation of MIC and MBC against MRSA isolates. As shown in Table 2, HNF and ANF were more active than TNF against ATCC 33591 in terms of MIC and MBC. ANF was found to be twofold more active than HNF according to MIC. The presence of many MRSA isolates in clinics highlights the need for improved potency of new compounds. The lead compounds revealed comparable MIC and MBC between ATCC 33591 and clinical MRSA isolates (KM-1 and KM-2). Again, TNF exhibited poorer activity than HNF and ANF against clinical isolates. Vancomycin is approved as the first choice for MRSA infection treatment. However, vancomycin resistance has emerged in *S. aureus*. The clinical isolates of vancomycin-intermediate *S. aureus* (VISA) were also employed to test the inhibitory potency of the lead compounds. A high anti-VISA effect for HNF and ANF with MIC between 9.7 and 19.5 μg/ml was detected. The compounds were also evaluated for activity against *E. coli*, which is a Gram-negative species. The selected agents were less effective against *E. coli* than against Gram-positive bacteria.

**Table 1:** The physicochemical properties of furanoquinone derivatives and diameter of inhibition zone of MRSA after treatment with furanoquinone derivatives.

Code	Formula	Alog*P*	HBD	HBA	MW (Da)	Inhibition zone (mm)
3a	C_12_H_7_NO_3_	2.27	1	3	213.19	0
3b	C_13_H_9_NO_3_	2.31	0	3	227.22	0
3c	C_19_H_13_NO_3_	3.89	0	3	303.31	0
4a (HNF)	C_12_H_7_NO_3_	2.27	1	3	213.19	23.49 ± 1.24
4b	C_13_H_9_NO_3_	2.31	0	3	227.22	23.10 ± 0.76
4c	C_19_H_13_NO_3_	3.89	0	3	303.31	0
5e	C_18_H_12_N_2_O_2_	4.06	1	3	288.30	0
5f	C_18_H_11_FN_2_O_2_	4.27	1	3	306.29	0
5g	C_19_H_14_N_2_O_3_	4.05	1	4	318.33	0
5h	C_19_H_14_N_2_O_2_	4.55	1	3	302.33	0
6d	C_14_H_10_N_2_O_3_	1.63	1	3	254.24	0
6e	C_18_H_12_N_2_O_2_	4.06	1	3	288.30	0
6f	C_18_H_11_FN_2_O_2_	4.27	1	3	306.29	0
6g	C_19_H_14_N_2_O_3_	4.05	1	4	318.33	15.34 ± 0.73
6h (TNF)	C_19_H_14_N_2_O_2_	4.55	1	3	302.33	9.12 ± 0.16
7a	C_14_H_9_NO_4_	2.09	0	4	255.23	0
7b	C_13_H_9_NO_5_S	2.04	0	5	291.28	0
8a (ANF)	C_14_H_9_NO_4_	2.09	0	4	255.23	24.35 ± 2.84
8b	C_13_H_9_NO_5_S	2.04	0	5	291.28	14.98 ± 15.47


**Each value represents the mean and SD (n = 3)*.*

**Table 2:** The MIC and MBC of MRSA, VISA, and *E. coli* after treatment with furanoquinone derivatives.

Strain	MIC (μg/ml)			MBC (μg/ml)		
		
	HNF	ANF	TNF	HNF	ANF	TNF
MRSA	9.7∼ 19.5	2.4 ∼ 9.7	625 ∼ 1250	19.5	19.5	625 ∼ 1250
KM-1	9.7 ∼ 19.5	19.5	>312.5	19.5 ∼ 39	19.5 ∼ 39	>312.5
KM-5	2.4 ∼ 19.5	2.4 ∼ 19.5	>312.5	19.5 ∼ 156	19.5 ∼ 39	>312.5
KV-1	9.7 ∼ 19.5	19.5	>312.5	3.9 ∼ 19.5	19.5 ∼ 39	>312.5
KV-5	9.7 ∼ 19.5	9.7 ∼ 19.5	>312.5	9.7 ∼ 39	39	>312.5
*E. coli*	39	19 ∼ 39	>312.5	312.5	312.5	>312.5


**Each value represents the mean and SD (n = 3)*.*

### Inhibitory Activity of HNF and ANF Against Planktonic MRSA

**FIGURE 1:**
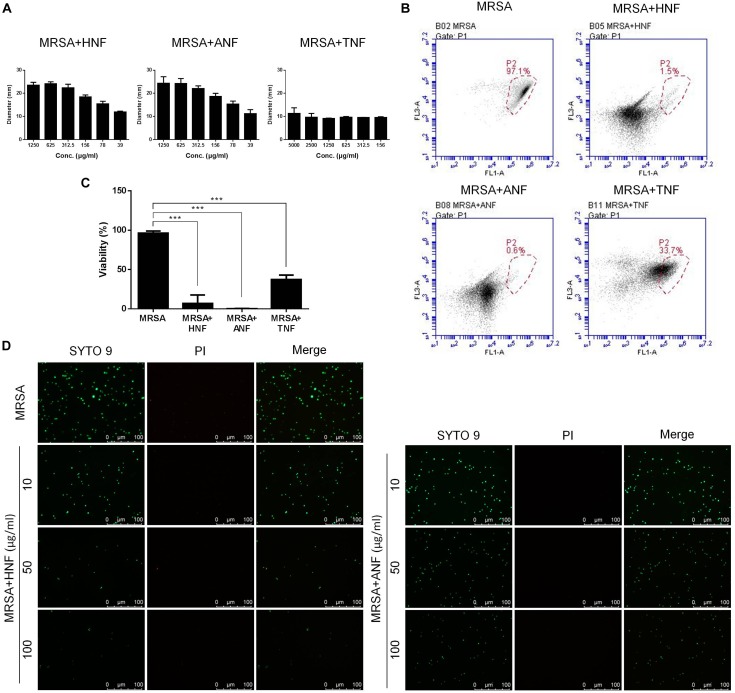
Determination of the anti-MRSA activity of furanoquinone derivatives. **(A)** Zone of inhibition measured from disk diffusion assay. Panel **(B)** represented flow cytometry diagram of live/dead MRSA. **(C)** MRSA viability measured from flow cytometry. **(D)** The planktonic live/dead MRSA strain viewed under fluorescence microscopy. All data are presented as the mean of three experiments ± SD. ^∗∗∗^*p* < 0.001.

The selected compounds at various concentrations were assessed using an agar diffusion test as shown in Figure 1A. HNF and ANF displayed a clear inhibition zone on the agar media against planktonic MRSA. Both compounds showed a dose-dependent inhibition zone at 39 ∼ 1,250 μg/ml. HNF and ANF had a similar effect on the inhibition zone. TNF displayed an inhibition zone at all concentrations tested, but the zone was smaller than with HNF and ANF. None of the zone diameters exhibited statistical significance. Flow cytometry was employed to measure the MRSA death rate. As shown in Figure 1B, the representative profiles of flow cytometry evidenced a high survival of MRSA without any treatment (97% in the red circle). HNF and ANF killed >98% of MRSA. TNF was less potent than HNF and ANF against the majority of MRSA. Figure 1C illustrates the viable bacteria percentage calculated from flow cytometry. ANF revealed greater reduction in viability compared to HNF, as ANF diminished the MRSA burden by more than 99%. HNF and ANF were chosen as the most promising of the derivatives based on an agar diffusion assay and flow cytometry. For this reason, both compounds were further evaluated by live/dead staining observed under a fluorescence microscope. Live MRSA with an intact membrane would be stained green by SYTO9-DNA interaction. Propidium iodide with red fluorescence is a molecule that diffuses to bacteria with a compromised membrane, implying cell death. As shown in Figure 1D, the non-treatment control exhibited a diffuse distribution of live MRSA with green staining. The green signal decreased following the increase of compound concentration from 10 to 100 μg/ml. Although the live bacteria were significantly reduced by lead compound treatment, the propidium iodide signal was very low. This indicates that HNF and ANF killed MRSA with limited cell-membrane damage.

### Inhibitory Activity of HNF and ANF Against Biofilm MRSA

**FIGURE 2:**
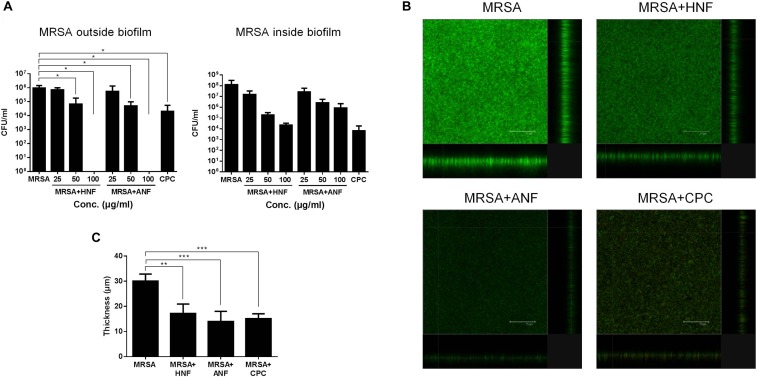
Determination of the antibacterial activity of HNF and ANF against biofilm MRSA. **(A)** MRSA CFU outside and inside the biofilm. **(B)** The three-dimensional images of biofilm visualized by confocal microscopy. **(C)** The corresponding biofilm thickness measured by confocal microscopy. All data are presented as the mean of three experiments ± S.D. ^∗∗∗^*p* < 0.001, ^∗∗^*p* < 0.01, ^∗^*p* < 0.05.

The bacteria in biofilm virulently resist antibiotics. Therefore, we examined whether HNF and ANF were also useful for eradicating biofilm MRSA. Figure 2A presents the effect of furanoquinone compounds on the MRSA CFU outside and inside the biofilm. Cetylpyridinium chloride (CPC) as an effective biofilm inhibitor was used as a reference. We found that HNF and ANF inhibited MRSA production outside the biofilm at concentrations of >25 μg/ml. A complete MRSA inhibition occurred at 100 μg/ml. The comparator agent CPC showed a preferential inhibition on MRSA outside the biofilm, which was comparable to 50 μg/ml HNF or ANF. An ideal antibacterial agent should disrupt and transport into the biofilm matrix to eradicate pathogens. The anti-MRSA activity of lead compounds toward bacteria inside the biofilm was dose-dependent. HNF prevented MRSA growth inside the biofilm more effectively than ANF. HNF at 100 μg/ml achieved a 4-log CFU reduction compared to the non-treatment control.

Figure 2B shows the antibiofilm effect seen by live/dead staining. The viable MRSA in the intact biofilm (control) revealed a massive, thick architecture. The green fluorescence was weakened by treatment of lead compounds and CPC, suggesting the restriction of bacterial colonization. Figure 2C illustrates the average thickness of biofilm calculated from confocal microscopy. The biofilm thickness could be reduced from 30.1 to 17.2, 14.0, and 15.1 μm by HNF, ANF, and CPC, respectively.

### Inhibitory Activity of HNF and ANF Against Cutaneous MRSA Infection

**FIGURE 3:**
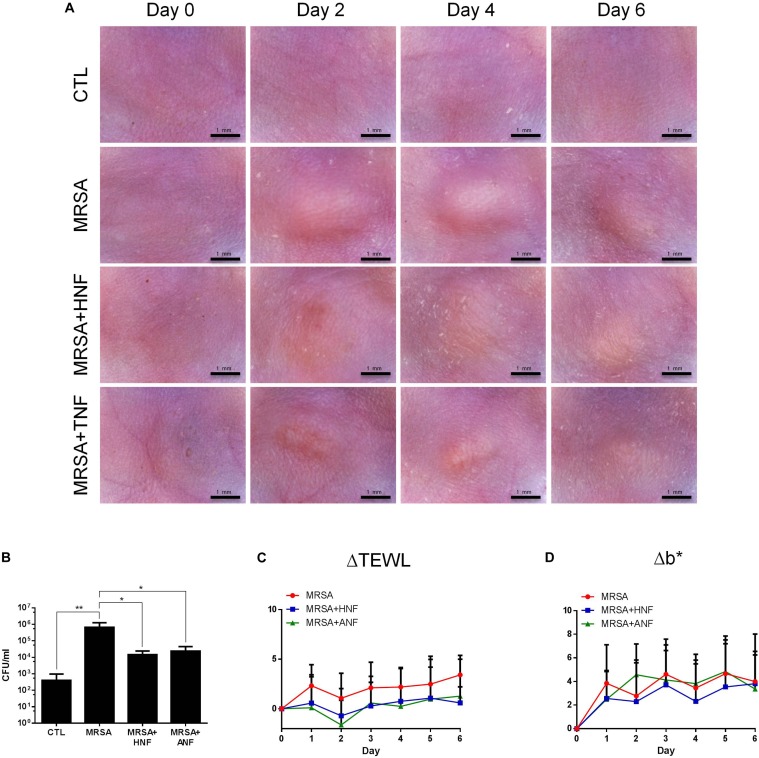
*In vivo* topical application of HNF and ANF against MRSA. **(A)** The skin surface of mice after treatment of MRSA at Days 0, 2, 4, and 6 viewed under handheld digital magnifier. **(B)** Survival of MRSA in mice skin treated with MRSA. **(C)** ΔTEWL of mice skin after treatment of MRSA from Day 0 to Day 6. **(D)** Δb^∗^ of mice skin after treatment of MRSA from Day 0 to Day 6. All data are presented as the mean of six experiments ± S.D. ^∗∗^*p* < 0.01, ^∗^*p* < 0.05.

The mouse was subcutaneously infected with the bacteria to determine the *in vivo* antimicrobial effect of HNF and ANF. Figure 3A depicts the appearance of the skin surface with and without infection 0, 2, 4, and 6 days post-injection. We observed the abscess after injection, finding that it resembled localized skin infection. The diameter of the abscess was about 3 mm. The topical application of HNF and ANF significantly reduced the lesional area after a 2-day treatment. The phyma nearly disappeared after a 6-day application of ANF. The MRSA CFU was detected 6 days post-infection as shown in Figure 3B. A 3-log increase of bacterial CFU in the skin could be seen by MRSA invasion. HNF and ANF suppressed the bacterial load in the skin relative to the vehicle control by about 2 logs. ANF and HNF were similarly efficient in the reduction of the MRSA burden in the skin. ΔTEWL (TEWL at the lesional site minus TEWL at the non-treatment control site) was detected daily to estimate the skin-barrier characteristic disrupted by MRSA-associated inflammation. As shown in Figure 3C, the MRSA-infected lesion displayed an increase of ΔTEWL from Day 1. This indicates MRSA’s deficient barrier property. Topical administration of both compounds significantly ameliorated the barrier function, approximating the TEWL baseline. The abscess generally exhibited a yellow–brown color. This appearance is analyzed by b^∗^ axis of colorimetry as depicted in Figure 3D. MRSA infection was significantly elevated to b^∗^ level in the injection region. The furanoquinone compounds had no potential effect on the improvement of b^∗^ although the compounds reduced the abscess size. This could be because we measured b^∗^ right on the lesional surface although the lesional size of the compound-treated skin was smaller than that of the vehicle-treated skin.

### Cutaneous Tolerance of HNF and ANF

**FIGURE 4:**
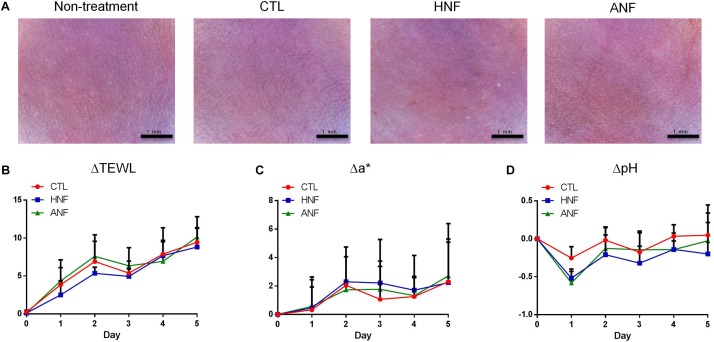
Skin tolerance examination of mouse skin by a 5-day treatment of topically applied HNF and ANF. **(A)** The skin surface of mice viewed under handheld digital magnifier. **(B)** ΔTEWL of mice skin from Day 0 to Day 5. **(C)** Δa^∗^ of mice skin from Day 0 to Day 5. **(D)** ΔpH of mice skin from Day 0 to Day 5. All data are presented as the mean of six experiments ± SD.

A prerequisite of the development of new candidates for antibiotics is the assurance of the safety use. HNF and ANF were administered on intact mouse skin to examine the possibility of irritation. As shown in Figure 4A, no redness or scaling was observed on cutaneous surface of the vehicle control. In contrast, mice treated with HNF and ANF revealed a slight redness. ΔTEWL was increased following the increase of the treatment duration for all groups tested (Figure 4B). This could be due to the ability of the aqueous vehicle to hydrate the stratum corneum and disturb the barrier function. Although the skin’s appearance showed a slight erythema after compound treatment, this trend was not detected by erythema quantification (a^∗^) since no groups demonstrated a significant difference (Figure 4C). A similar tendency was observed in the skin-surface pH (Figure 4D). The cutaneous tolerance study suggested that HNF and ANF showed only marginal irritation.

### Anti-MRSA Mechanisms of HNF and ANF

**FIGURE 5:**
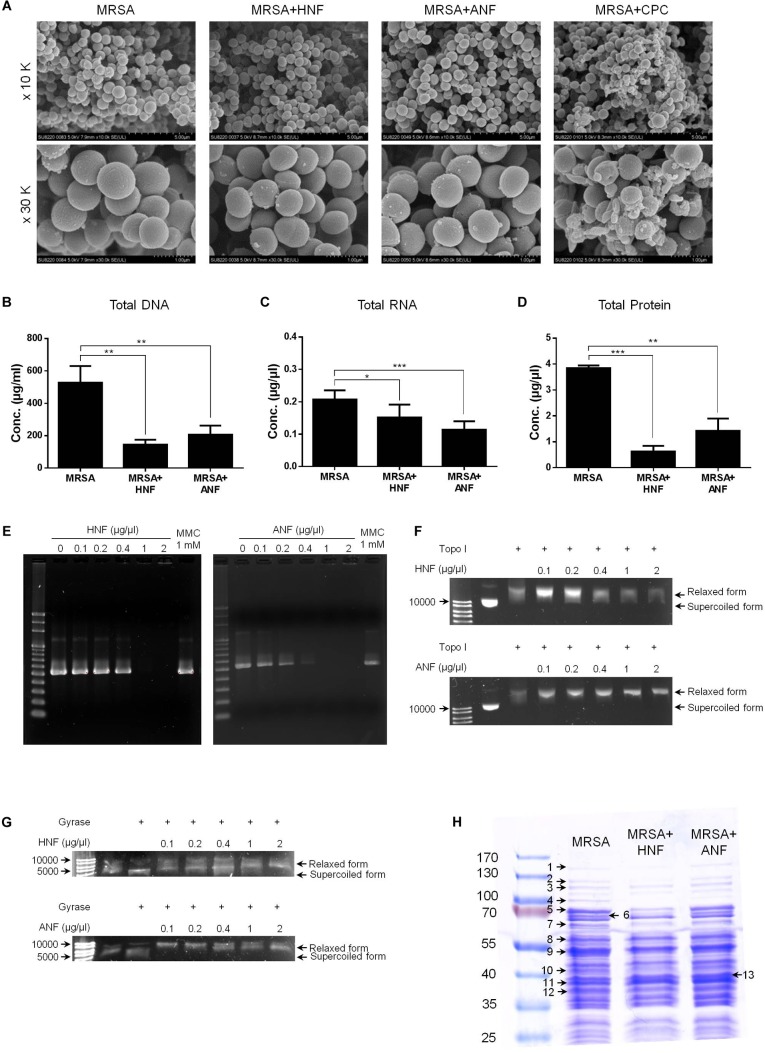
Anti-MRSA mechanisms of HNF and ANF. **(A)** Morphological changes of MRSA viewed under SEM at the magnification of 10 K or 30 K. **(B)** Total DNA amount in MRSA. **(C)** Total RNA amount in MRSA. **(D)** Total protein amount in MRSA. **(E)** Taq DNA polymerase in PCR. **(F)** Topoisomerase I in wrapping assay. **(G)** DNA gyrase in wrapping assay. **(H)** The protein change of MRSA analyzed by SDS-PAGE and MALDI-TOF/TOF mass. All data are presented as the mean of three experiments ± SD. ^∗∗∗^*p* < 0.001, ^∗∗^*p* < 0.01, ^∗^*p* < 0.05.

Further experiments aimed to explore the antibacterial mechanisms of furanoquinone compounds against MRSA. SEM allowed us to directly visualize MRSA integrity and morphology. As shown in Figure 5A, the untreated bacteria exhibited a smooth surface without debris. CPC as a comparator agent altered the cell structure. The enlarged image (30 K magnification) showed membrane destruction and the leakage of cytoplasm materials, resulting in the bacterial shrinkage. Most of the bacteria remained intact after the HNF and ANF treatment. Some cells revealed an irregular shape without obvious membrane disintegration and cell lysis. The total DNA, RNA, and protein of furanoquinone-treated MRSA are quantified as shown in Figure 5B–D, respectively. The genomic DNA analysis indicated that the content of DNA decreased significantly in furanoquinone-treated bacteria. HNF or ANF exerted about a threefold reduction of DNA concentration compared to the untreated control. Total RNA was also decreased by HNF and ANF although the reduction level was minor as compared to the DNA decrease. ANF caused a 63% inhibition of the total protein content than the control. A significant degradation was shown with HNF-treated MRSA, leading to an 84% decrease.

Enzymes such as DNA polymerases and topoisomerases are potential targets of antibacterial mechanisms. We hypothesized that HNF and ANF could affect these enzymes, thus disrupting the transcription from DNA to RNA and the translation from RNA to proteins. The gene products of DNA polymerase, topoisomerase I, and gyrase were chosen for the enzyme-inhibition study. As illustrated in Figure 5E, furanoquinone compounds significantly inhibited PCR products of DNA polymerase. This inhibition activated by HNF and ANF was concentration-dependent, with stronger activity with ANF than with HNF. Mitomycin C (MMC), an anticancer agent known to induce DNA damage via interstrand DNA crosslink with no effect on DNA polymerase activity, was used as the comparator. Figure 5E demonstrates a momentous presence of PCR products by MMC application. The compounds were also utilized for targeted enzyme inhibition using a wrapping assay to analyze the suppression of supercoiling by topoisomerase I and gyrase. As shown in Figure 5F, HNF and ANF at concentrations from 0.1 to 2 μg/μl did not inhibit DNA relaxation activity of topoisomerase I. On the other hand, furanoquinone treatment markedly reduced gyrase activity (Figure 5G). Based on these results, we can suggest that HNF and ANF deactivated both DNA polymerase and gyrase consistent with a dual-targeting mechanism.

MRSA was analyzed by SDS-PAGE for the soluble proteins as shown in Figure 5H. Protein bands of MRSA treated by vehicle, HNF, and ANF were somewhat different. There were 13 proteins differentially expressed after the treatment of compounds. The mass spectra and the related data of these proteins are summarized in the Supplementary Materials. Most of these bands were apparently shallow after compound intervention as summarized in Table 3. The alteration in the proteins caused by HNF and ANF was detected by mass spectrometry. HNF produced a distinct reduction in DNA-directed RNA polymerase (band Nos. 1 and 2), acetyltransferases (band Nos. 5 and 7), and elongation factors Tu and Ts (band Nos. 9 and 12). However, the change in these proteins was minor for ANF. Both compounds significantly downregulated elongation factor G (band No. 4).

**Table 3:** Differentially expressed proteins of MRSA after treatment of lead compounds HNF and ANF.

Band no	Protein	Accession no.	MW (Da)	Matched-peptides	Sequence Coverage % (SCORE)	Ratios to MRSA^a,b^		Biological function
							
						HNF	ANF	
1	DNA-directed RNA polymerase subunit beta	P60279	133.418	20	112(18%)	0.345	1.323	DNA-dependent RNA polymerase catalyzes the transcription of DNA into RNA using the four ribonucleoside triphosphates as substrates
2	DNA-directed RNA polymerase subunit beta’	P60286	134.748	25	125(25%)	0.152	1.084	DNA-dependent RNA polymerase catalyzes the transcription of DNA into RNA using the four ribonucleoside triphosphates as substrates.
3	Aconitate hydratase A	P63434	99.135	22	120(34%)	0.698	1.245	Involved in the catabolism of short chain fatty acids (SCFA) via the tricarboxylic acid (TCA) (acetyl degradation route) and probably the 2-methylcitrate cycle 1 (propionate degradation route).
	Isoleucyl-tRNA synthetase	Q8NX29	97.364	20	94(30%)			Catalyzes the attachment of isoleucine to tRNA(lle).
4	Elongation factor G	P68791	76.877	25	186(49%)	0.025	0.262	Catalyzes the GTP-dependent ribosomal translocation step during translation elongation.
5	Formate acetyltransferase	Q7A1W9	85.250	40	226(70%)	0.240	0.883	Activated by pfl-activating enzyme under anaerobic conditions via generation of an organic free radical.
6	Formate acetyltransferase	Q7A1W9	85.278	36	226(49%)	0.382	0.771	Activated by pfl-activating enzyme under anaerobic conditions via generation of an organic free radical.
7	Dihydrolipoyllysine-residue acetyltransferase component of pyruvate dehydrogenase complex	Q8NX76	46.452	17	88(44%)	0.330	0.710	The pyruvate dehydrogenase complex catalyzes the overall conversion of pyruvate to acetyl-CoA and CO(2). It contains multiple copies of three enzymatic components: pyruvate dehydrogenase (El), dihydrolipoamide acetyltransferase (E2) and lipoamide dehydrogenase (E3).
8	Phosphoenolpyruvate carboxykinase (ATP)	Q8NVZ8	59.599	26	168(56%)	0.860	1.010	Involved in the gluconeogenesis.
9	Elongation factor TU	P64029	43.148	16	95(49%)	0.496	0.944	This protein promotes the GTP-dependent binding of aminoacyl-tRNA to the A-site of ribosomes during protein biosynthesis.
	2-phosphoglycerate dehydratase	P64079	46.036	14	90(47%)			Catalyzes the reversible conversion of 2-phosphoglycerate into phosphoenolpyruvate.
10	Phosphoglycerate kinase	P6S82I	28.722	12	87(61%)	0.428	0.841	
11	Ornithine carbamoyltransferase	QKNX44	37.781	15	128(61%)	0.943	1.713	Reversibly catalyzes the transfer of the carbamoyl group from carbamoyl phosphate (CP) to the N(epsilon) atom of ornithine (ORN) to produce L-citrulline.
12	Elongation factor Ts	Q8NWZ6	32.618	13	77(40%)	0.484	0.986	Associates with the EF-Tu. GDP complex and induces the exchange of GDP to GTP. It remains bound to the aminoacyl-tRNA.
13	Ornithine carbamoyltransferase	Q8NX44	37.739	17	137(71%)	0.884	1.673	Reversibly catalyzes the transfer of the carbamoyl group from carbamoyl phosphate (CP) to the N(epsilon) atom of ornithine (ORN) to produce L-citrulline.


*^*a*^Ratios to MRSA indicated the fold changes in protein amount between HNI- and ANF-treated samples vs. MRSA control samples, respectively. The higher ratios (>1.0) mean that the protein expression levels were increased upon treatments of compounds, while lower ratios (<1.0) indicate that the proteins were downregulated under the exposure to compounds. ^*b*^Analyzing the gel images using Gene Tools software. MW, molecular weigh*.

### *In silico* Docking

**FIGURE 6:**
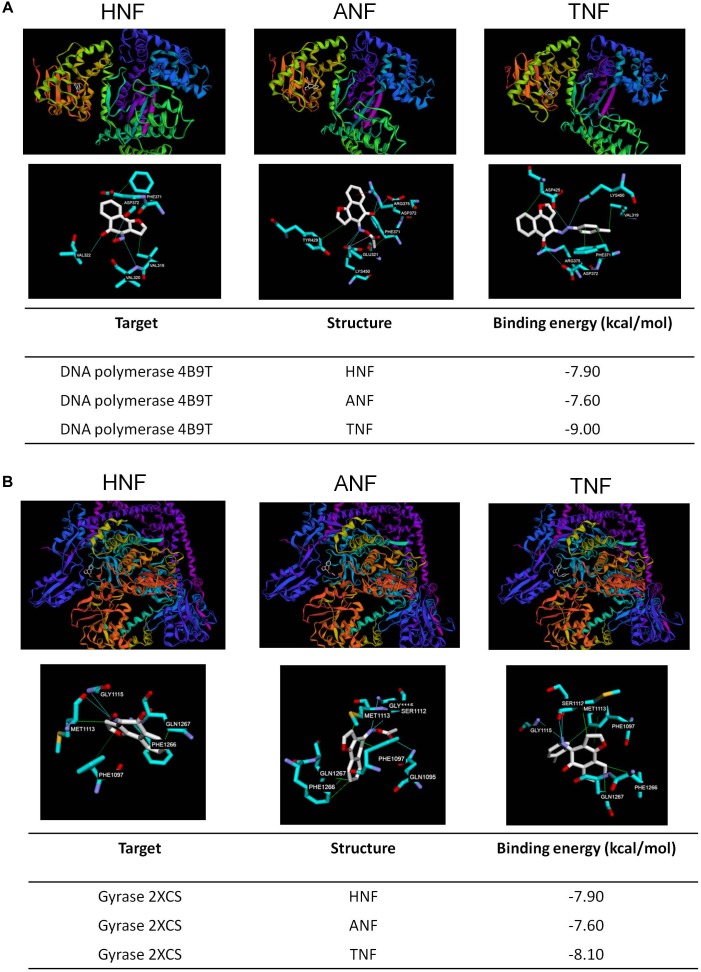
Docking poses and binding energy (kcal/mol) of HNF, ANF, and TNF at the enzymes. **(A)** The docking pose between the compounds and DNA polymerase (PDB code 4B9T). **(B)** The docking pose between the compounds and gyrase (PDB code 2XCS).

Induced fit docking is a tool to anticipate the binding affinity between the screened proteins and the active agents. To determine the possible binding modes of HNF and ANF, we docked the compound structures against DNA polymerase and gyrase. TNF with the lower anti-MRSA activity was also used in the docking experiment for comparison. Figure 6A shows the 3D molecular interaction of furanoquinones against DNA polymerase. All three compounds were located in the same binding pocket. HNF caused van der Waals interaction and hydrogen binding at the active sites with Val319, Val320, Val322, Phe371, and Asp372 residues. The binding between ANF and DNA polymerase was stabilized through van der Waal interaction, hydrogen binding, salt bridge, and π-cation interaction. The amino acids involved included Phe371, Tyr429, Glu321, Asp372, and Lys450. Val319, Phe371, Asp372, Asp425, Arg375, and Lys450 located in the binding pocket played vital roles in the conformation of TNF. The calculated binding energy between the compounds and DNA polymerase (PDB code 4B9T) was identified as shown in Figure 6A. HNF and ANF had a comparable binding energy of -7.9 and -7.6 kcal/mol, respectively. TNF revealed a lower energy (-9.0 kcal/mol) as compared to the others.

Figure 6B shows the docking poses of the compounds with gyrase. The three compounds were docked in the same binding pocket with similar conformations. Hydrophobic interaction, hydrogen bonds, and π–π stacking could be observed between HNF and the pocket. The most involved amino acids of gyrase in the binding of HNF were Met1113, Phe1266, Gln1267, and Gly1115. ANF imparted hydrophobic interaction with Phe1097, Met1113, Phe1266, and Gln1267, and a hydrogen binding with Gln1095, Ser1112, and Gly1115. TNF projected into Phe1097, Met1113, Phe1266, and Gln1267 to form hydrophobic interaction. Hydrogen binding was built between TNF and Ser1112, Met1113, and Gly1115. TNF and gyrase Phe1097 formed stable π–π stacking. The binding energy between the compounds and gyrase was of comparable strength (Figure 6B).

## Discussion

The continued development of antibiotic resistance in superbug microbes has limited the spectra of clinically approved drugs. The introduction of a new class of antibacterial agents is required to improve the management of drug-resistant pathogens. To address this need, we designed furanoquinone derivatives of a novel class of polymerase and gyrase inhibitors. HNF and ANF had the strongest anti-MRSA activity as assessed in both forms of planktonic and biofilm bacteria. We found some correlations between the compound structure and anti-MRSA activity. The furanoquinone derivatives with linear structure were totally devoid of MRSA inhibition. The angular structure was required to exhibit the growth inhibition. The presence of alkoxime linker in the angular furanoquinones was of utmost importance to showing the MRSA inhibition. The alkoxime-containing compounds conjugated with –H (HNF) and –COMe (ANF) were particularly promising. An increase in the bulkiness of the substituent at the alkoxime part (4c and 8b) was associated with less activity against MRSA. This may have been due to the smaller size of HNF and ANF compared to 4c and 8b for facile binding to the pocket of the target enzymes. Most of the compounds containing hydrazino linker showed no inhibition. The introduction of methoxyphenyl (6g) or the methylphenyl (6h, TNF) group in the hydrazino part led to a moderate potency. However, the replacement of methyl moiety in the methoxyphenyl group of 6g with fluorine (6f) ended the activity.

The data of MIC and MBC also confirmed the superior anti-MRSA activity of HNF and ANF. An agent is considered bactericidal but not bacteriostatic as its MBC is no more than 4x MIC (French, 2006). Our data indicated that both compounds were bactericidal against MRSA. HNF and ANF showed broad activity against drug-resistant *S. aureus*, including clinical isolates of MRSA and VISA. There are 3% of MRSA strains classified as VISA (Zhang et al., 2015). Many MRSA-infected patients do not respond favorably to vancomycin. The potential of the furanoquinones was also tested against *E. coli*. The MBC for *E. coli* killing was greater than 300 μg/ml. The lower activity on Gram-negative bacteria could be due to the complex cell-wall composition. There are two cell-envelope membranes in *E. coli*, leading to the limitation of interaction and penetration of antibacterial agents. This result suggested a potential specificity of HNF and ANF for Gram-positive microbes.

The resistance of bacteria in biofilm is attributed to the penetration barrier and the persistence of slow-growing bacteria with a low metabolic rate (Nair et al., 2016). MRSA is known to create the biofilm by producing exopolysaccharides, DNA, and proteins (McCarthy et al., 2015). Biofilm acts as a barrier restricting antibiotic delivery, resulting in the increased survival rate against antibiotics and host defense. HNF and ANF could reduce MRSA CFU in biofilm and biofilm height. This suggests that the furanoquinone-derived compounds could facilely enter into the biofilm for MRSA killing. The decrease in biofilm formation could be related to the decreased MRSA survival rate. CPC is a quaternary ammonium surfactant revealing strong antibiofilm activity by destabilizing the bacterial membrane (Latimer et al., 2015). The antibiofilm effect of HNF and ANF achieved a comparable level to CPC. Some of the MRSA can be dispersed from the biofilm to the perimeter in the final stage of development (Chung and Toh, 2014). HNF and ANF showed a greater MRSA inhibition outside the biofilm as compared to CPC. Biofilm represents different gene expression profiles and physiologies compared with the planktonic form (Abouelhassan et al., 2018). Our results indicated the efficient eradication of both forms by HNF and ANF. HNF and ANF were profiled in the murine efficacy experiment. The result demonstrated the translation from *in vitro* to *in vivo* anti-MRSA activity. Both compounds were effective in reducing MRSA-infected abscess and bacterial burden with the recovery of the skin barrier. The inhibition of MRSA colonization by furanoquinones was expected to impede the cutaneous inflammation for improving barrier function. The topical application of the lead compounds did not induce remarkable skin irritation although a slight erythema was visualized.

The genes involved in transcription and translation, as well as the metabolic process related to DNA, RNA, or protein synthesis, strongly affect bacterial viability. The data pertaining to the total amount of DNA, RNA, and protein in furanoquinone-treated MRSA showed an obvious reduction compared with the control. The loss of these components could be due to the cell-membrane damage’s causing leakage of cytoplasm materials or the inhibition of enzyme systems related to DNA replication (Yang et al., 2018). The SEM images displayed very slight cell-membrane damage of the MRSA treated by the lead compounds. Fluorescence microscopy of the live/dead bacteria showed very few bacteria with a damaged membrane; these damaged bacteria were stained red. These results implied that DNA replication suppression but not membrane disintegration governed the anti-MRSA activity of HNF and ANF. The physicochemical nature of both compounds could make feasible their permeation into MRSA for enzyme inhibition. We hypothesized that the targets of HNF and ANF were synthetic enzymes to selectively interfere with DNA and the subsequent RNA or protein synthesis. DNA polymerases and topoisomerases are major virulence determinants to impact MRSA infection persistence via their effect on viability (Lakhundi and Zhang, 2018). Our data validated the role of DNA polymerase inhibitors for both HNF and ANF. DNA topoisomerase enzymes function to regulate DNA topology for maintaining essential bacterial performance during replication and transcription (Savage et al., 2016). Topoisomerases such as topoisomerase I and gyrase are potential targets for antibacterial agents (Ranjan et al., 2017). The wrapping assay indicated the important role of gyrase but not topoisomerase I for MRSA eradication by the lead compounds. Gyrase belongs to the type IIA topoisomerase family. It relieves torsional tension by introducing negative supercoils to DNA during replication, which is vital for bacterial survival (Franco-Ulloa et al., 2018). Gyrase is the target for fluoroquinolone antibiotics. Thus, it can be a potential target for new anti-MRSA agents. Furanoquinone compounds might inhibit gyrase and further block DNA replication, resulting in the total DNA lessening and cell death. Inhibition of gyrase in *S. aureus* can affect membrane morphology, creating a rough surface and blebs (Franci et al., 2018). Our SEM images verified a slight alteration of the normal shape and some blebs on the MRSA surface after HNF or ANF treatment.

We also used proteomics to elucidate the possible anti-MRSA mechanisms of furanoquinones. Besides DNA polymerase, RNA polymerase might be the possible target for HNF because of a significant reduction in both subunits β and β′. The transcriptional process should be controlled for *S. aureus* to adjust the environment for survival (Elgaher et al., 2016). Rifamycins are a group of conventional antibiotics used to bind with the RNA polymerase subunit β to kill bacteria. RNA polymerase can also be the target of HNF. Since the enzyme is required through the development of *S. aureus* biofilm (Weiss et al., 2017), the inhibition of RNA polymerase might be the reason of biofilm disruption by HNF treatment. HNF but not ANF also apparently downregulated formate acetyltransferase. Acetyltransferase can act as a detoxifying enzyme to acetylate non-metabolizing carbohydrates to retard the reentry into MRSA (Stogios et al., 2014). The acetyltransferase superfamily is demonstrated to be reduced in MRSA in the presence of fusidic acid (Luo et al., 2013). Fusidic acid also works by blocking elongation factor G on the ribosomes, thus interfering with protein synthesis (Koripella et al., 2012). A similar mechanism was observed for furanoquinone compounds. The drug-resistant bacteria can upregulate the elongation factor superfamily to defend against antibiotics in order to survive. Furanoquinones may have been inhibiting elongation factor G, Tu, and Ts to eradicate MRSA.

DNA polymerase and gyrase were possible targets for furanoquinone compounds. We applied docking calculation and measured the free energy of the binding of the compounds using crystalline structures of DNA polymerase and gyrase. Induced fit docking indicated that HNF and ANF identically bound to both enzymes. The formation of van der Waal interaction, hydrogen binding, and π–π stacking contributed to good stability and strong affinity to these proteins. The binding energy for the lead compounds on both proteins was -8 ∼-7 kcal/mol. It can be recognized that the binding energy score of <-9 kcal/mol is meaningful to produce a direct dock for deactivating the proteins. The binding energy of HNF and ANF may be insufficient to directly interact with DNA polymerase and gyrase to induce protein inhibition. Modification of the 4-position of furanoquinones was related to the antibacterial potency. TNF showed weaker anti-MRSA activity than HNF and ANF, but their binding energy was comparable. The result suggests that, besides the direct targeting to the enzymes, HNF and ANF might function with the other DNA or proteins in suppressing the activity of DNA polymerase and gyrase. The mechanisms of furanoquinones can be more complex than our study’s investigation showed. The precise mechanisms of HNF and ANF interference with enzymatic activity were not determined in detail in this work. Additional mechanistic study is needed to clarify the actual target of the lead compounds. Although molecular docking can assist the elucidation of the enzymatic target, it cannot promise the correlation with antibacterial activity. The other factors such as bacterial membrane permeation and the influence of the microenvironment in cytoplasm also largely impact the outcome of antibacterial efficiency (Werner et al., 2014).

The MRSA-related growth inhibition by HNF and ANF was related to DNA polymerase and gyrase. Both proteins in prokaryotic cells show considerable difference as compared to eukaryotes (Sissi and Palumbo, 2010). DNA gyrase is even absent in eukaryotes (Chiriac et al., 2015). Furanoquinones exhibit a specific toxicity against MRSA with limited impact on humans. The dual inhibition of both enzymes further delays the occurrence of bacterial resistance because the simultaneous mutation on two targets is very low (Durcik et al., 2018). Topical administration of anti-MRSA agents for treating cutaneous infection have the advantages over oral or systemic counterparts of avoiding side effects, lowering cost, and decreasing drug resistance (Mohammad et al., 2015). However, most of the antibiotics currently used to treat MRSA such as vancomycin, tigecycline, and rifampicin manifest a large molecular size (Kurosu et al., 2013). The large size may impede the penetration into the biofilm and skin. The small MW of HNF and ANF is beneficial for cutaneous delivery.

## Conclusion

Furanoquinone derivatives with imine moiety were successfully synthesized via regiospecific and stereospecific modes. The resulting compounds were examined for their capability to eradicate MRSA infection *in vitro* and *in vivo*. The attempt had been made to discover potent and safe anti-MRSA agents for cutaneous infection treatment. Among 19 compounds tested, HNF and ANF had powerful anti-MRSA potency, which was proved by agar diffusion assay, MIC, MBC, and live/dead staining. Both compounds also showed great potential to inhibit biofilm formation with bacterial viability reduction. The lead compounds demonstrated antibacterial activity against skin MRSA infection with very slight irritation. The inhibition of DNA polymerase and gyrase could be the mechanism for bactericidal activity on MRSA. The docking assay indicated that the compounds interacted with DNA polymerase and gyrase although the binding was only mild. HNF and ANF might also suppress the upstream proteins to indirectly inhibit the enzymes. HNF and ANF generally revealed a comparable MRSA lethality. A small increase in antibacterial activity could be observed with ANF as compared to HNF according to MIC, cell death determined by flow cytometry, the gross appearance of skin abscess, and DNA polymerase inhibition. On the other hand, the total protein analysis and proteomic profiles demonstrated a greater loss of proteins by HNF than ANF. Our results suggested that the lead furanoquinone compounds possessed potential anti-MRSA activity for further antibiotic development.

## Ethics Statement

### Humans

The clinical isolates of MRSA (KM-1 and KM-5) were isolated from Kaohsiung Medical University Hospital. KV-1 and KV-5 were vancomycin-intermediate *S. aureus* (VISA) strains found in Kaohsiung Medical University Hospital provided by Dr. Po-Liang Lu. The clinical isolates were taken from infected patients using a protocol approved by the institutional review board at Kaohsiung Medical University Hospital. The patients provided written informed consent to participate.

### Animals

A female BALB/c mouse (8 weeks old) was purchased from the National Laboratory Animal Center (Taipei, Taiwan). This study was carried out in accordance with the principles of the Basel Declaration and recommendations of Guidelines for the Care and Use of Laboratory Animals of Chang Gung University. The protocol was approved by the Chang Gung University.

## Author Contributions

S-CY initiated the study and drafted the manuscript. J-YF involved in the design of all experiments. S-CY and K-WT carried out the experiments. C-HL and AA analyzed data and wrote the manuscript. J-YF supervised the entire project. C-HT reviewed critically and approved the final manuscript. All authors read and approved the final manuscript.

## Conflict of Interest Statement

The authors declare that the research was conducted in the absence of any commercial or financial relationships that could be construed as a potential conflict of interest.

## Footnotes

1 www.matrixscience.com

2 http://bio-hpc.ucam.edu/achilles/

## Supplementary Material

SCHEMES 1, 2 The synthetic routes to the furanoquinone derivatives.
