# Supplementary figures and images for: Discovery of Furanoquinone Derivatives as a Novel Class of DNA Polymerase and Gyrase Inhibitors for MRSA Eradication in Cutaneous Infection
Source: Front Microbiol. 2019 May 29;10:1197. doi: 10.3389/fmicb.2019.01197 (PMC6549599; doi:10.3389/fmicb.2019.01197)

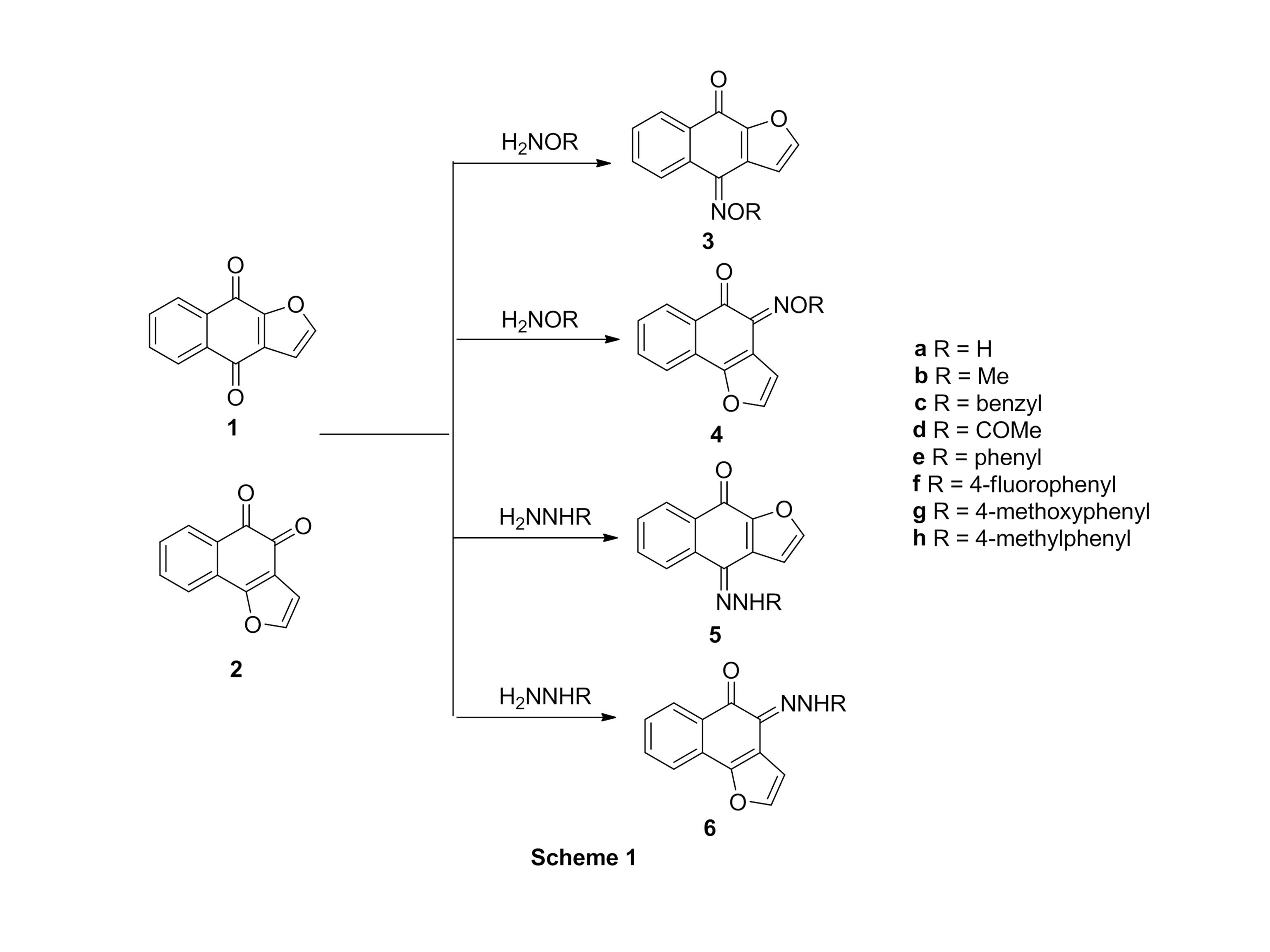

Supplement: SCHEMES 1, 2 — The synthetic routes to the furanoquinone derivatives. [file Image_1.JPEG]

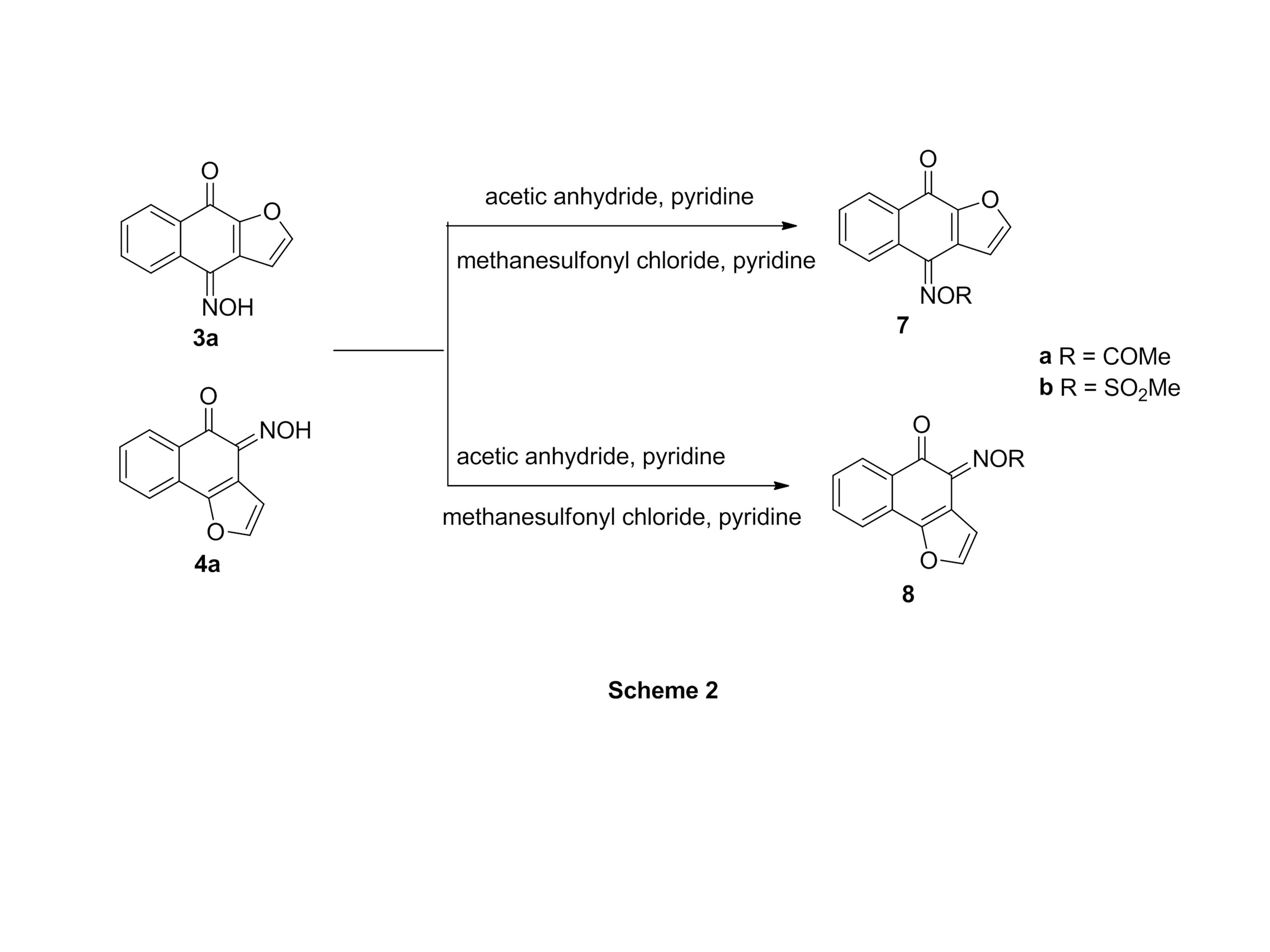

Supplement: Supplementary file 2 [file Image_2.JPEG]
