# Supplementary material for: Discovery of Furanoquinone Derivatives as a Novel Class of DNA Polymerase and Gyrase Inhibitors for MRSA Eradication in Cutaneous Infection
Source: Front Microbiol. 2019 May 29;10:1197. doi: 10.3389/fmicb.2019.01197 (PMC6549599; doi:10.3389/fmicb.2019.01197)
Supplement: Supplementary file 3 [file Presentation_1.PPT]

## Slide 1
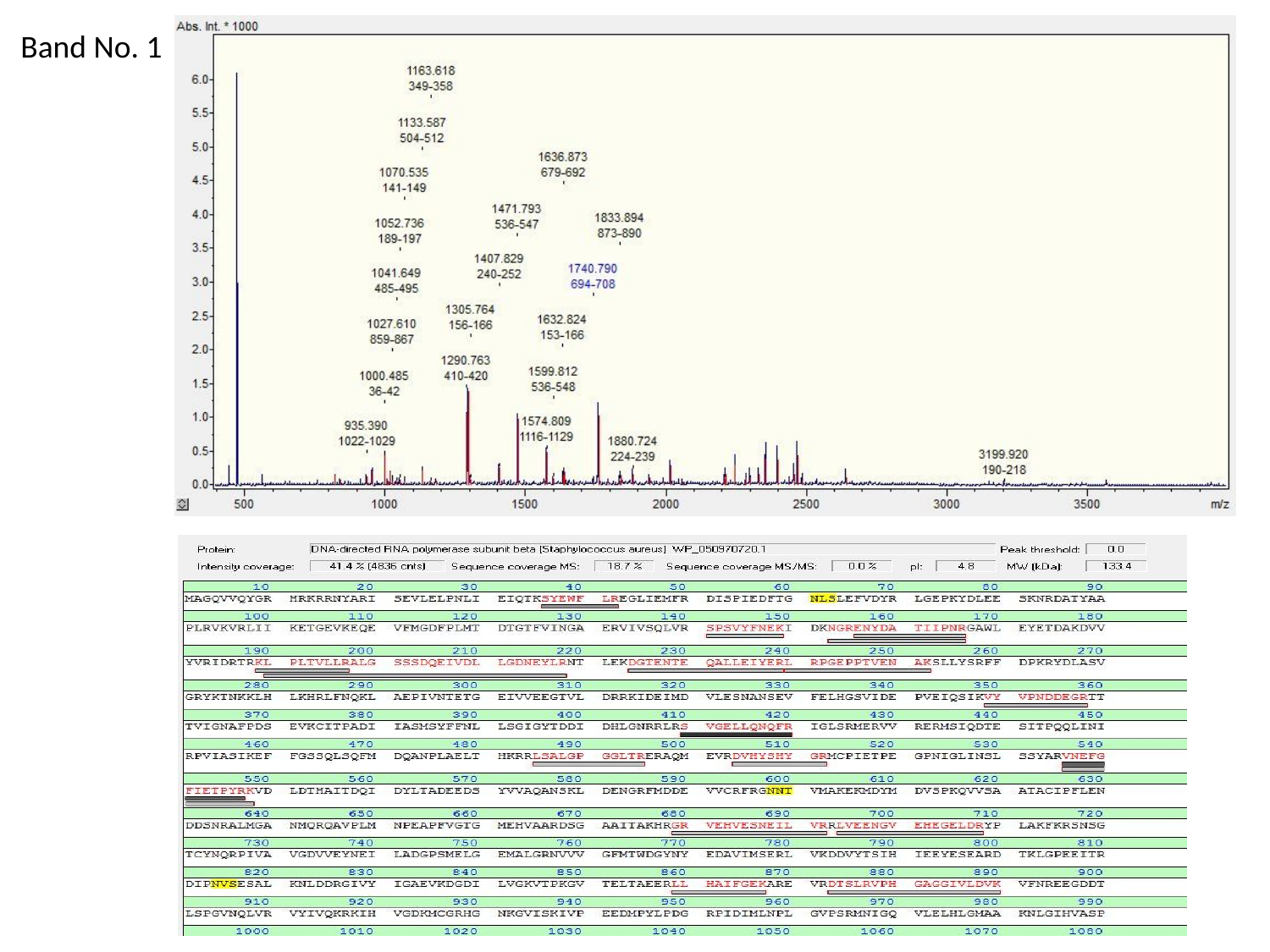

Band No. 1

## Slide 2
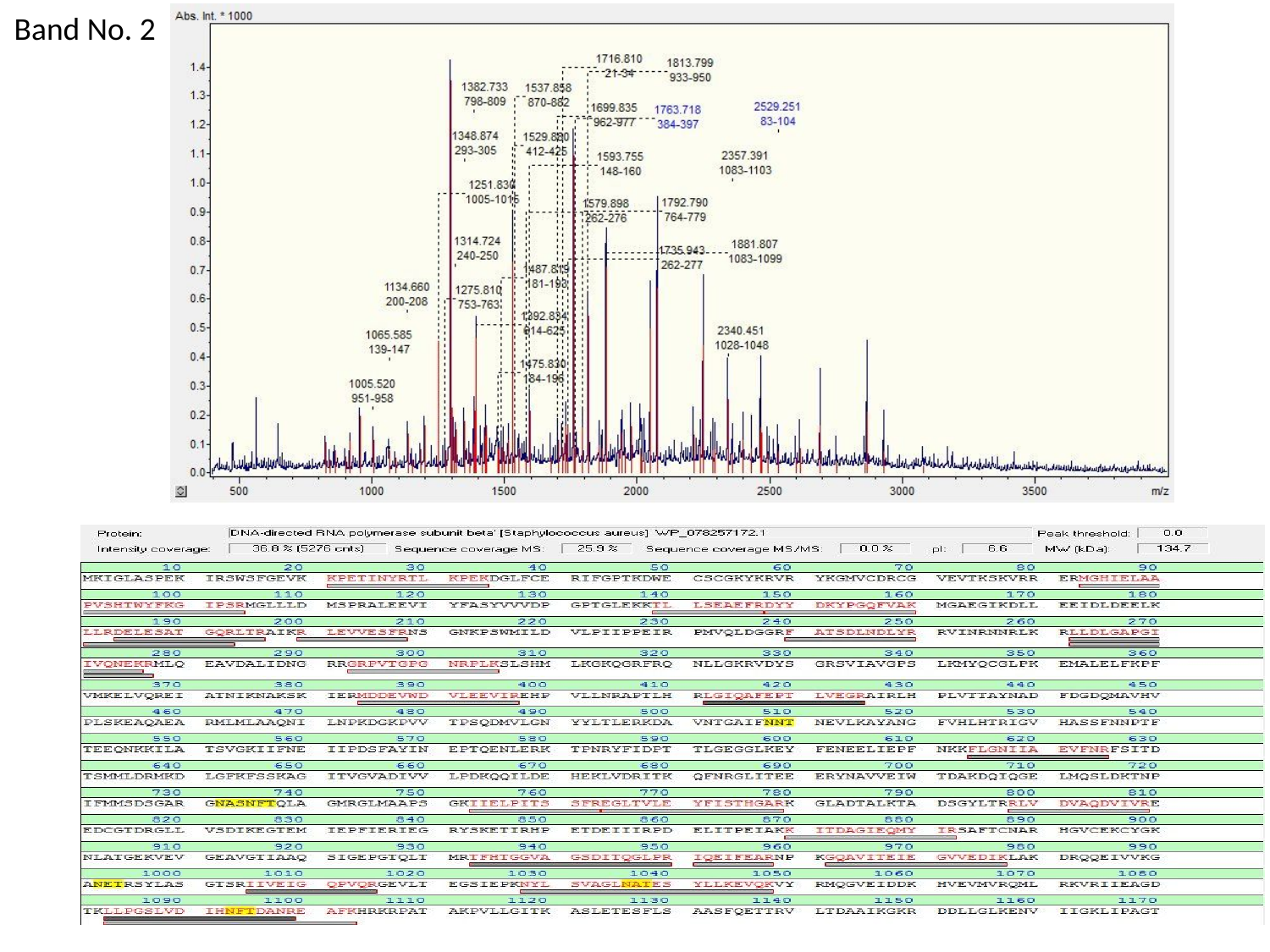

Band No. 2

## Slide 3
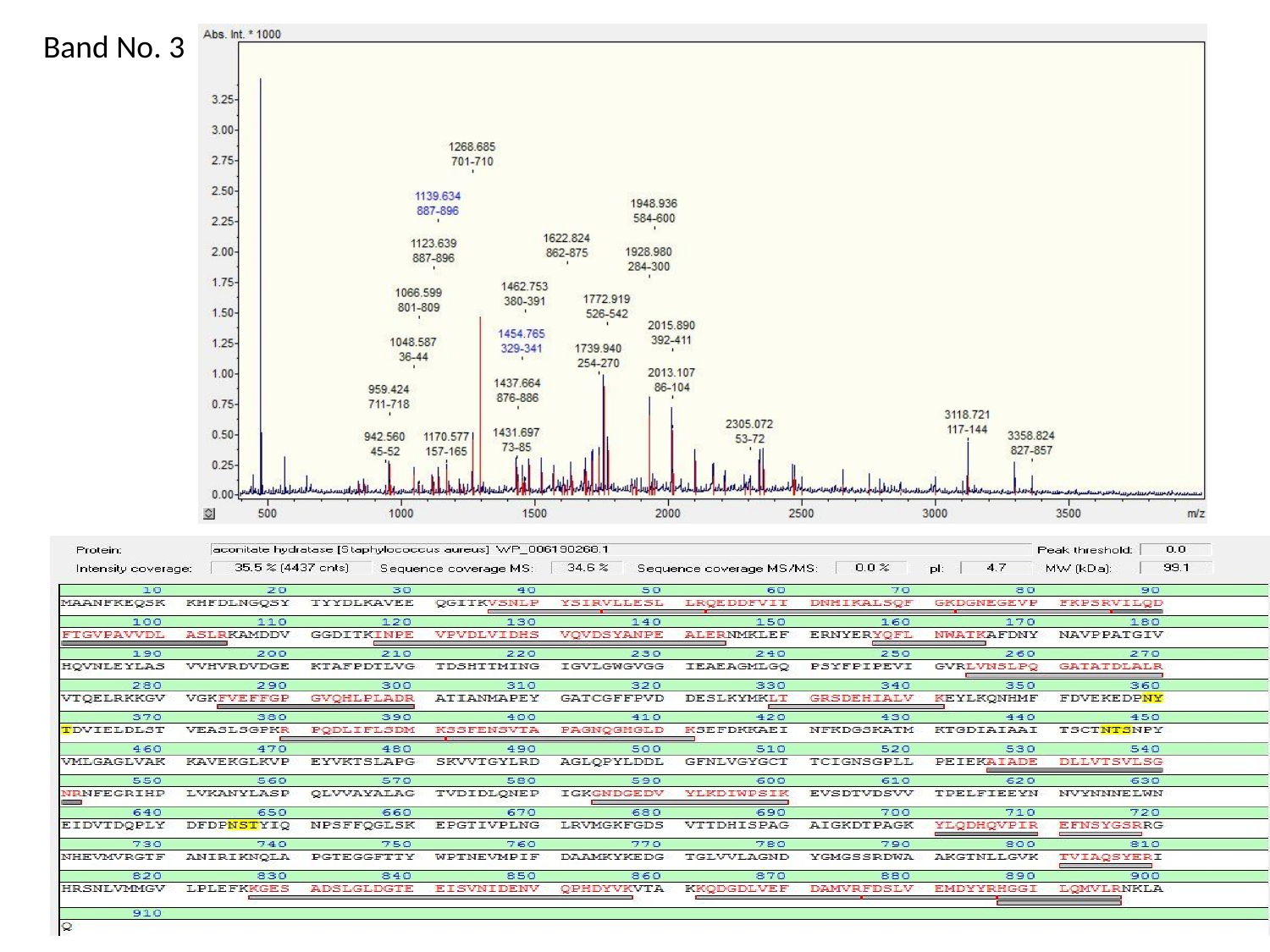

Band No. 3

## Slide 4
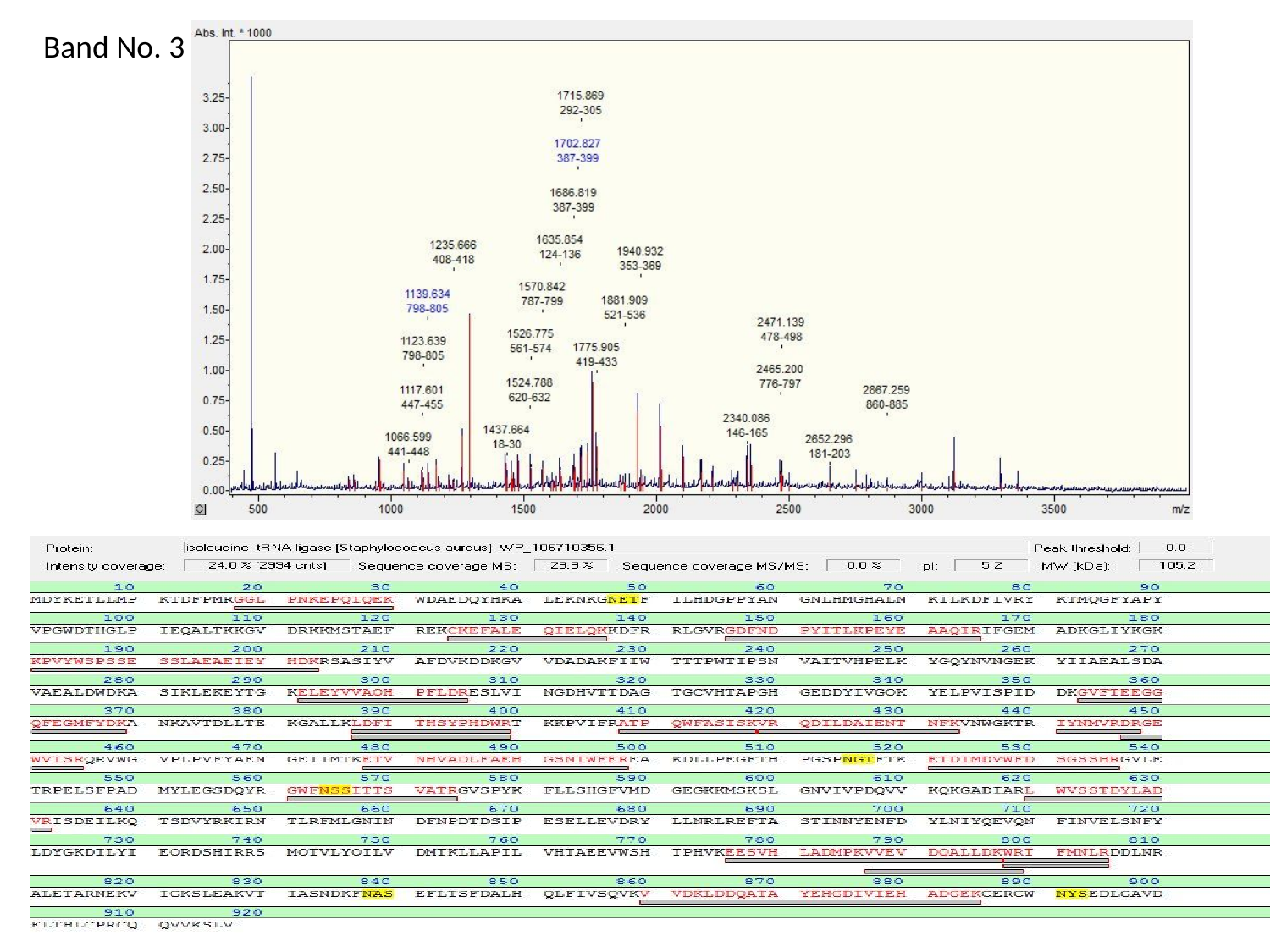

Band No. 3

## Slide 5
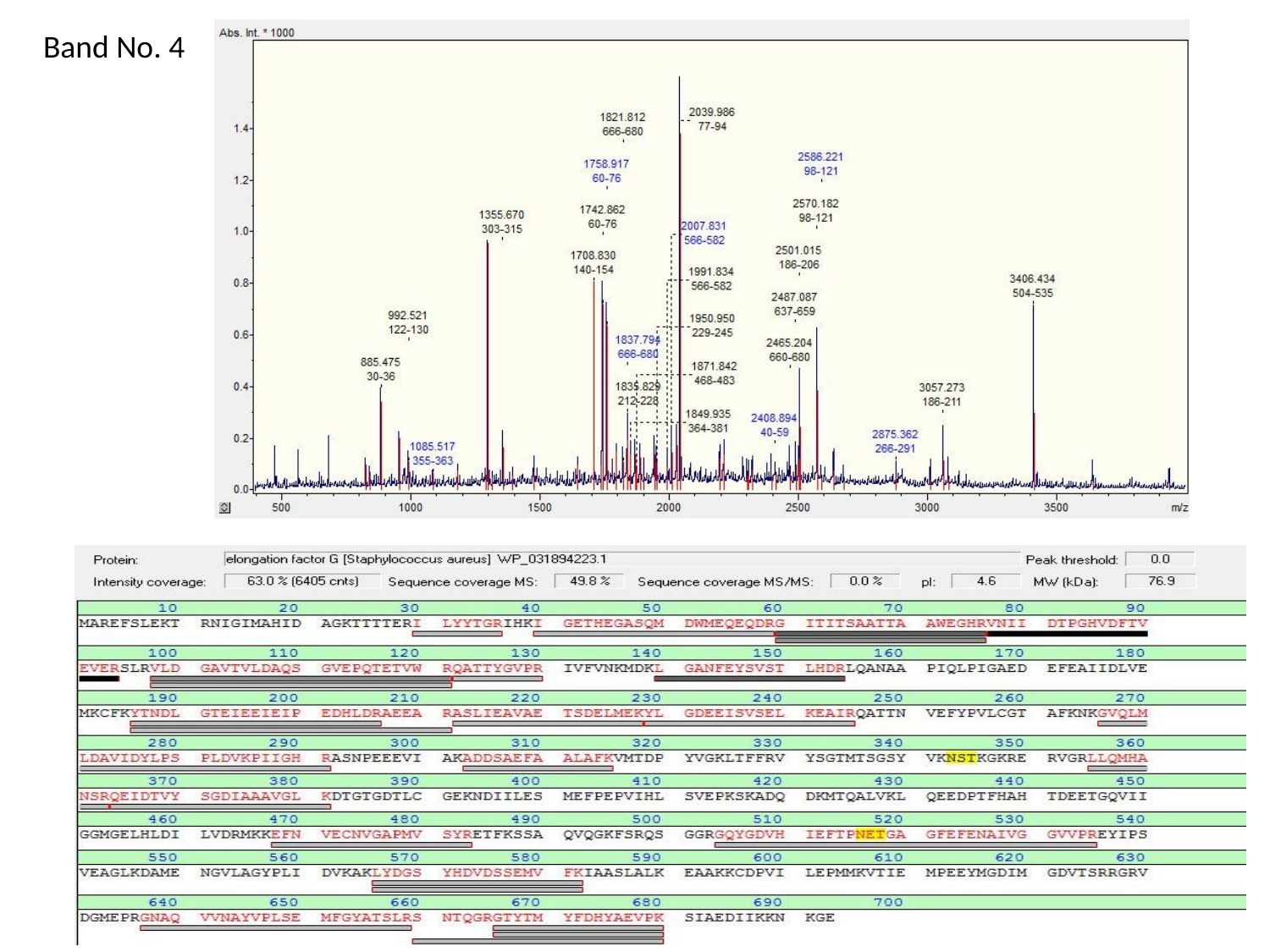

Band No. 4

## Slide 6
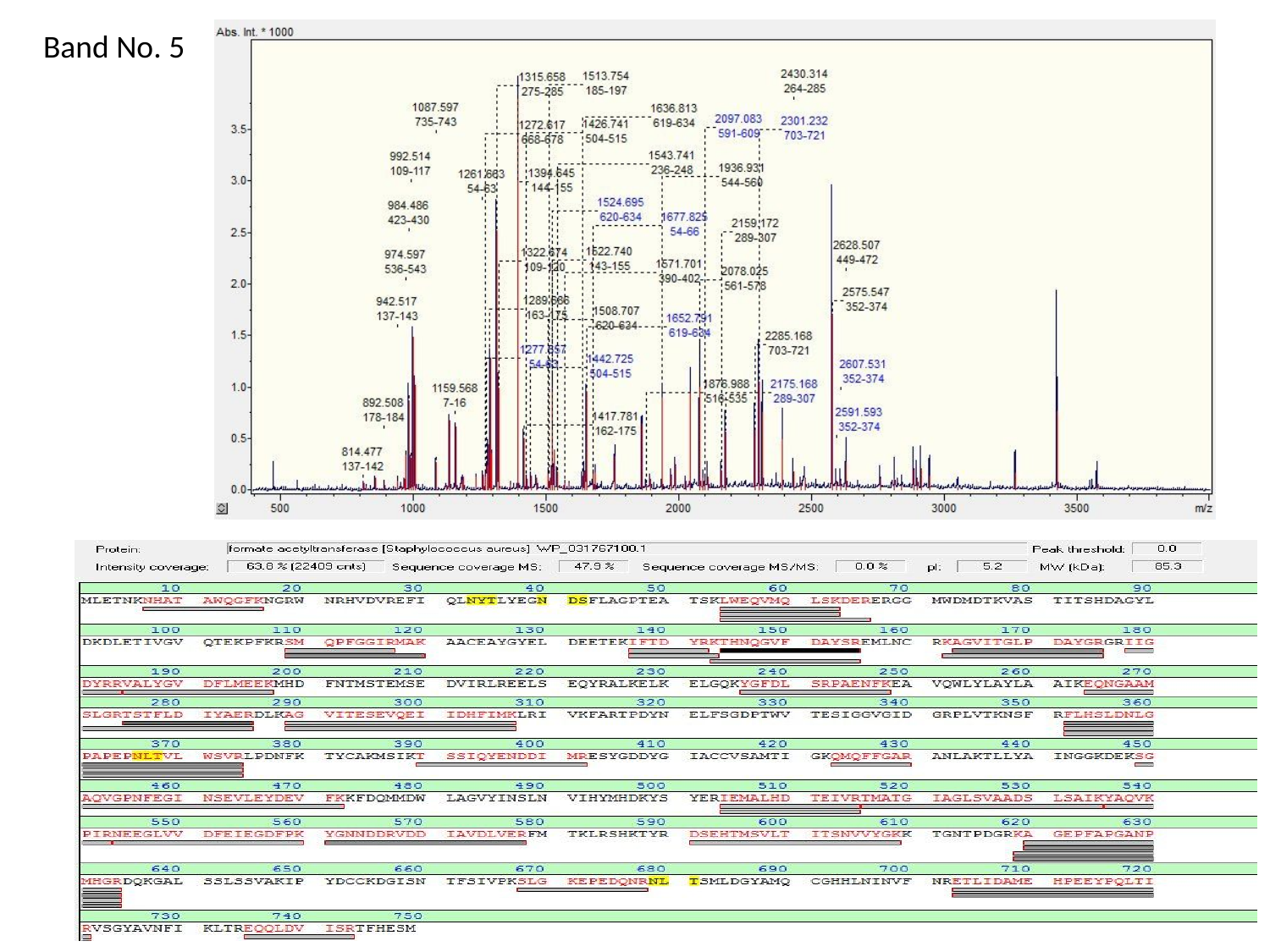

Band No. 5

## Slide 7
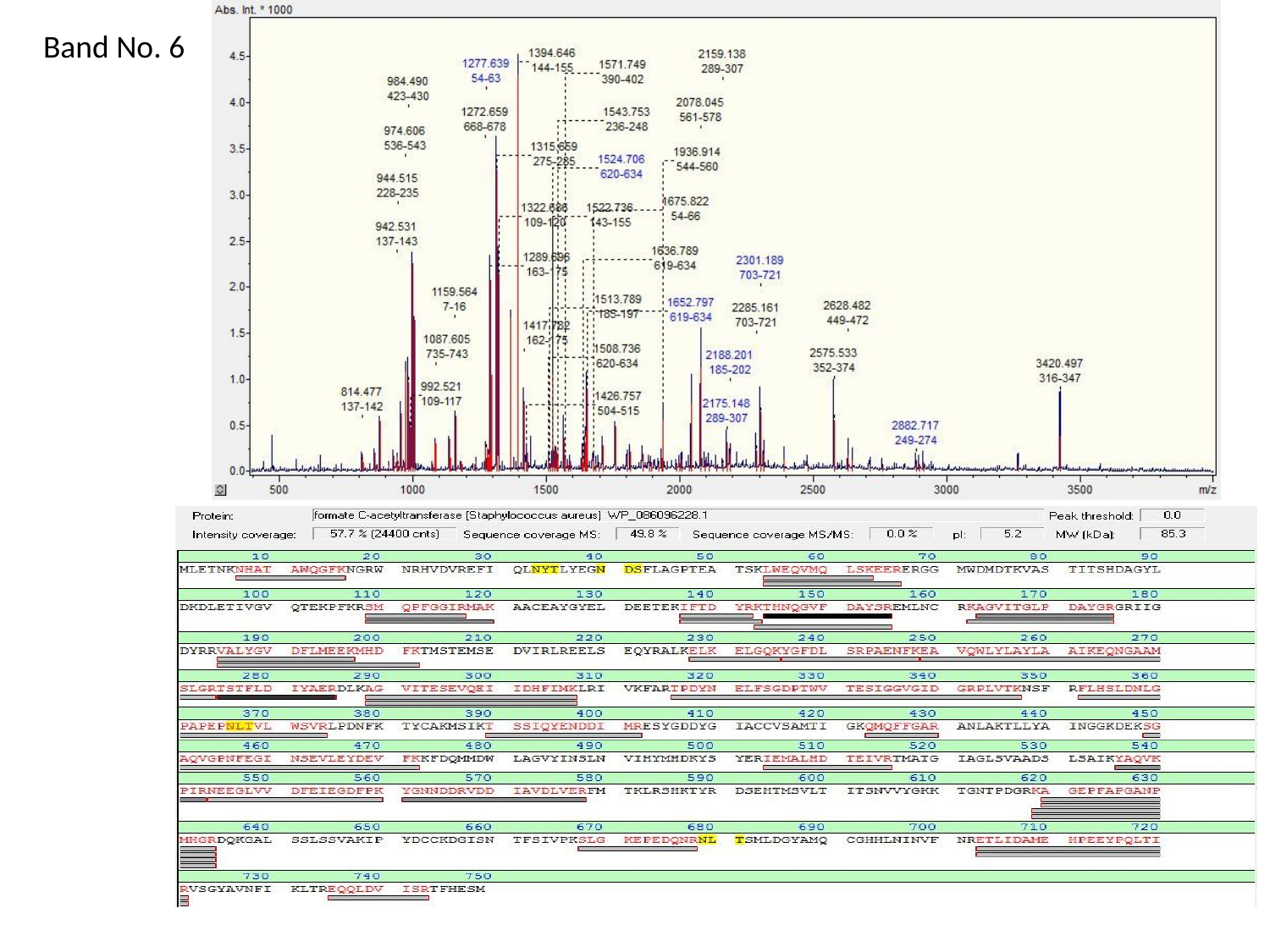

Band No. 6

## Slide 8
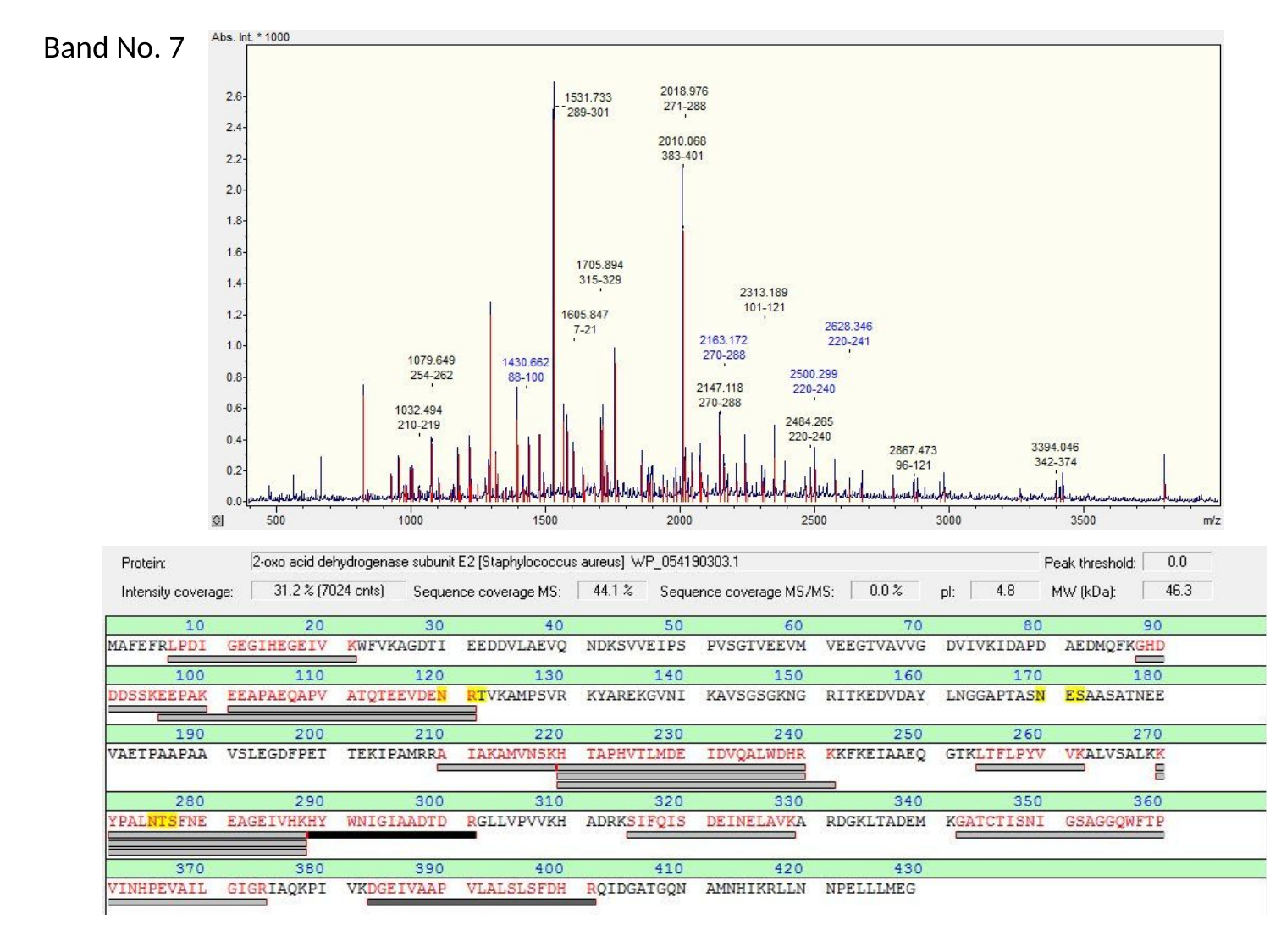

Band No. 7

## Slide 9
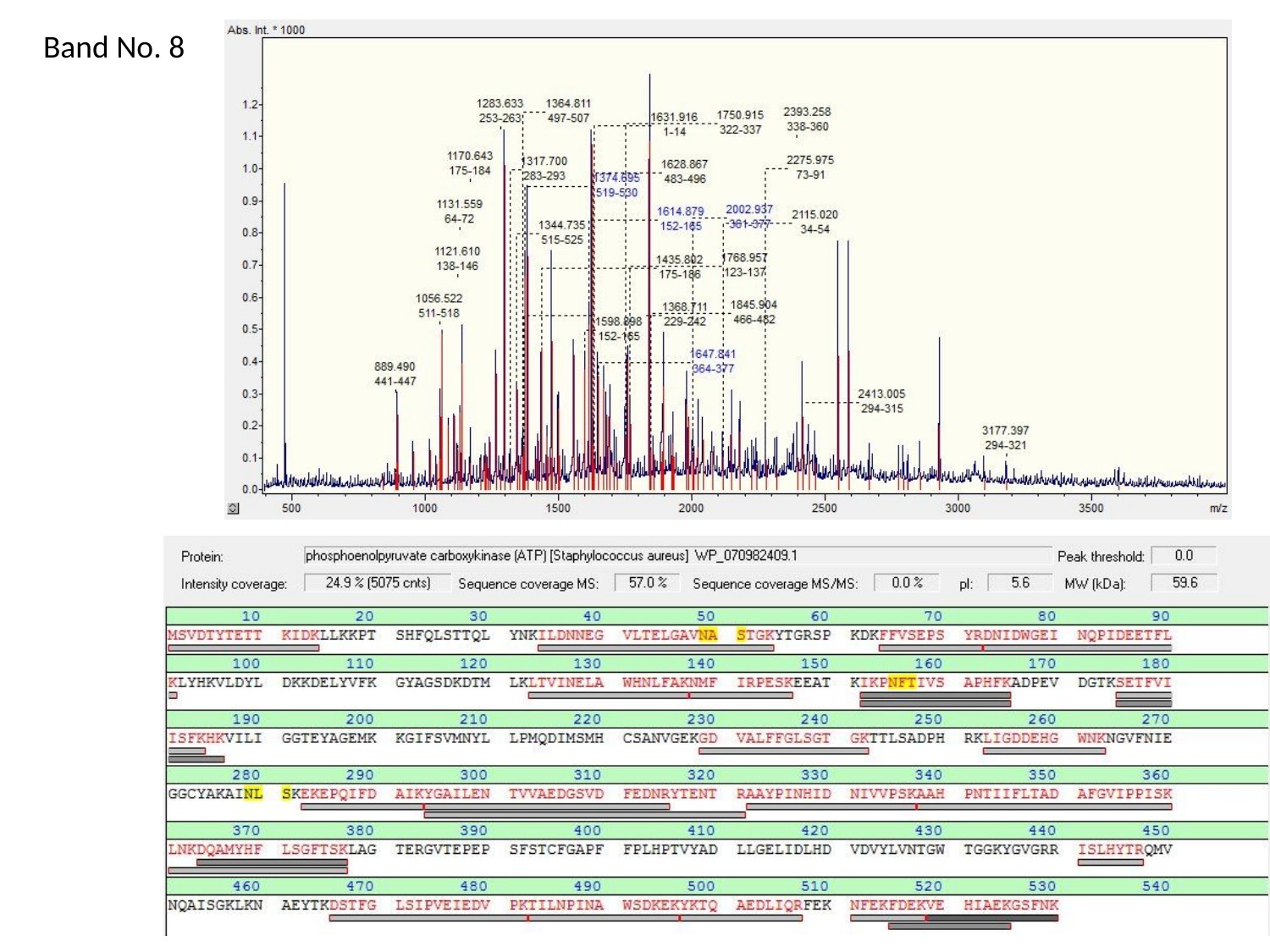

Band No. 8

## Slide 10
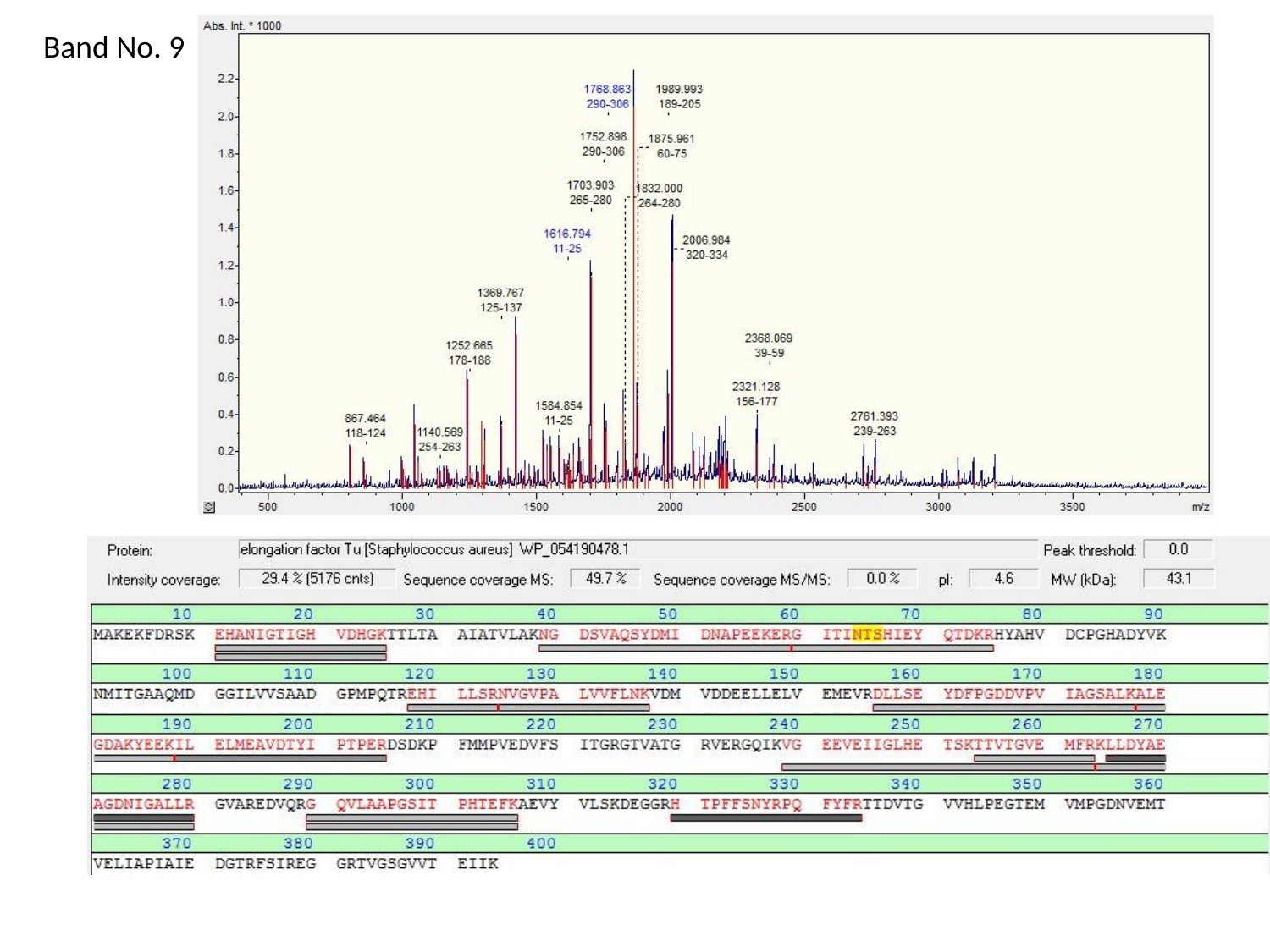

Band No. 9

## Slide 11
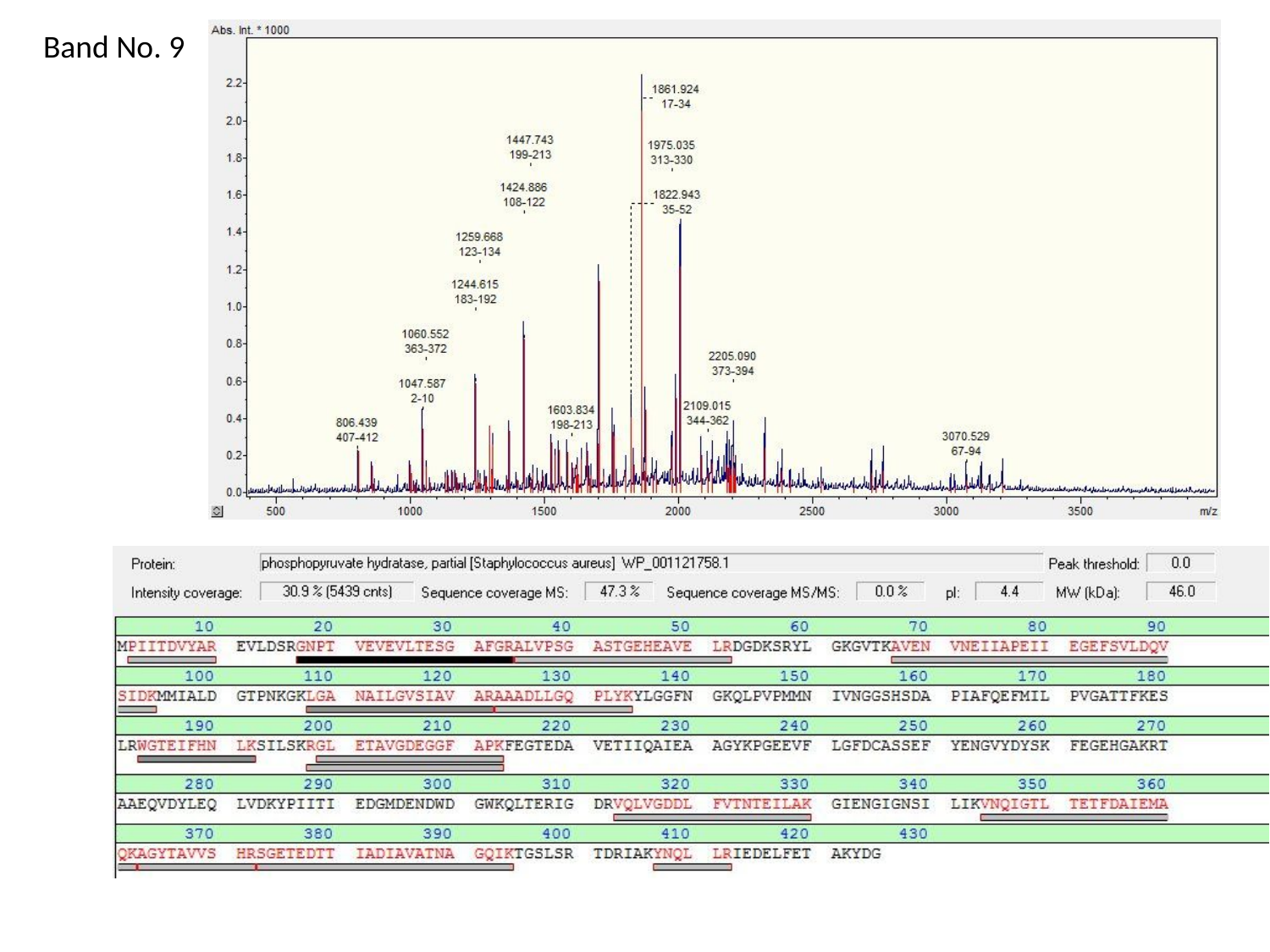

Band No. 9

## Slide 12
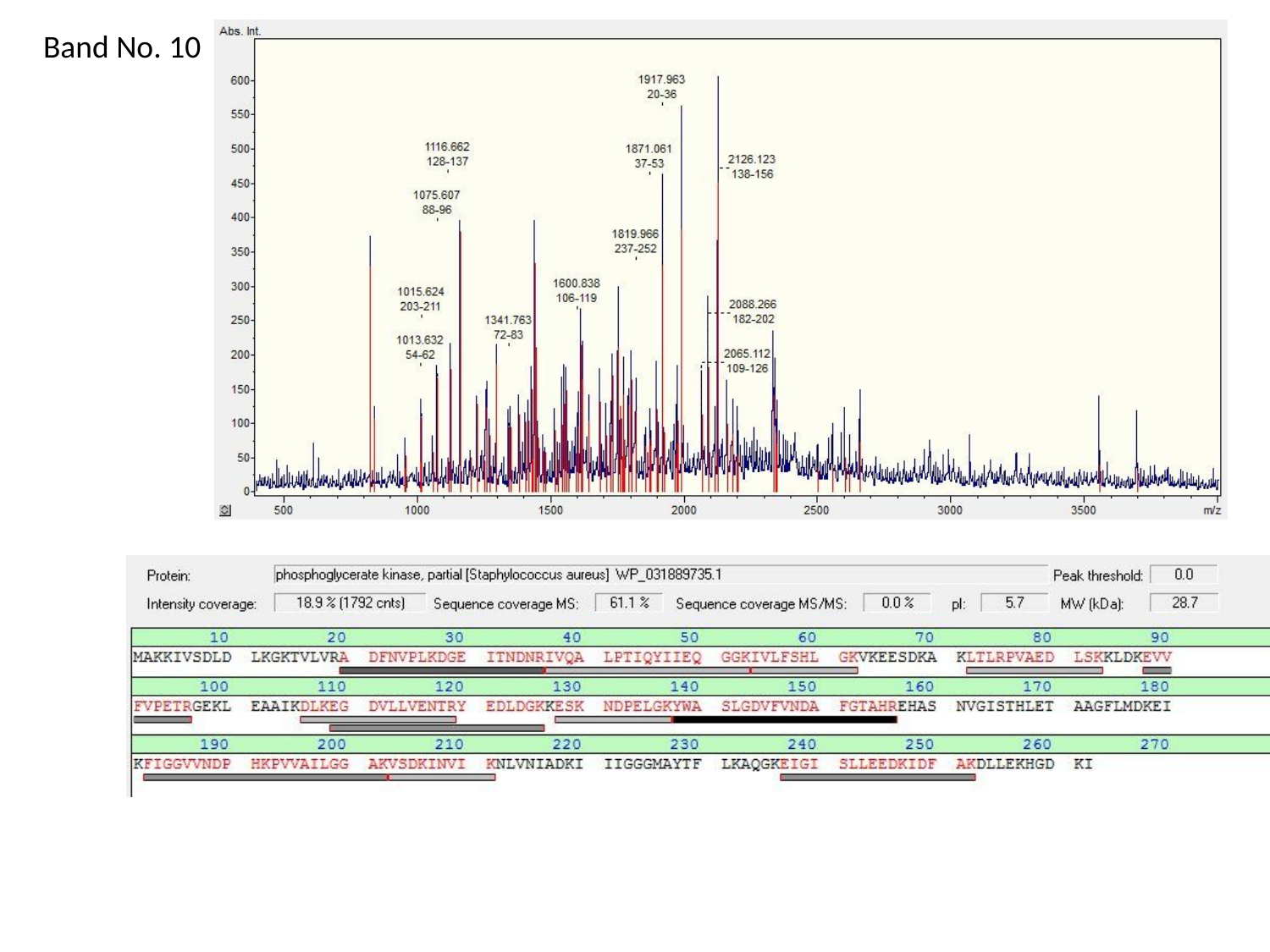

Band No. 10

## Slide 13
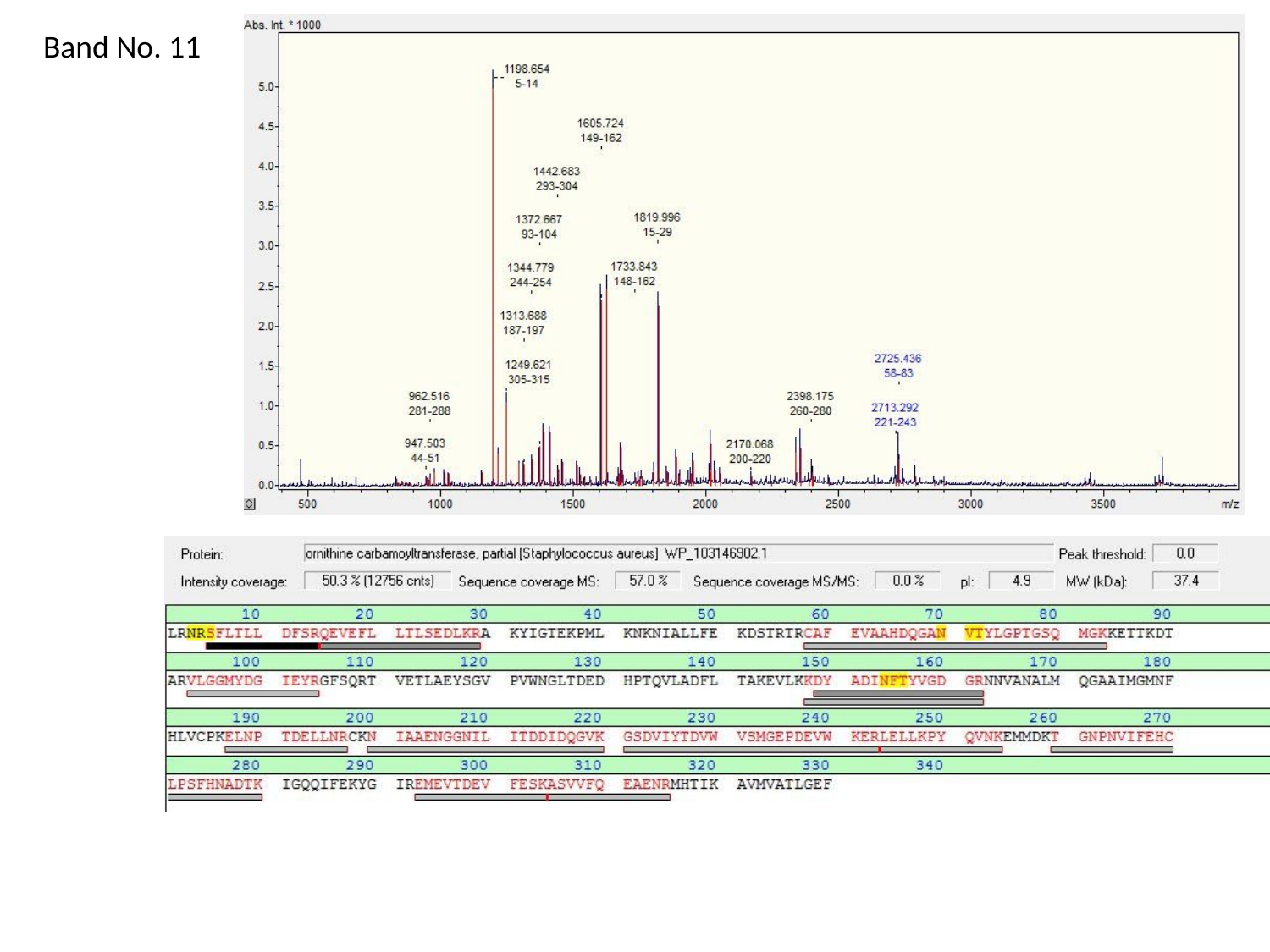

Band No. 11

## Slide 14
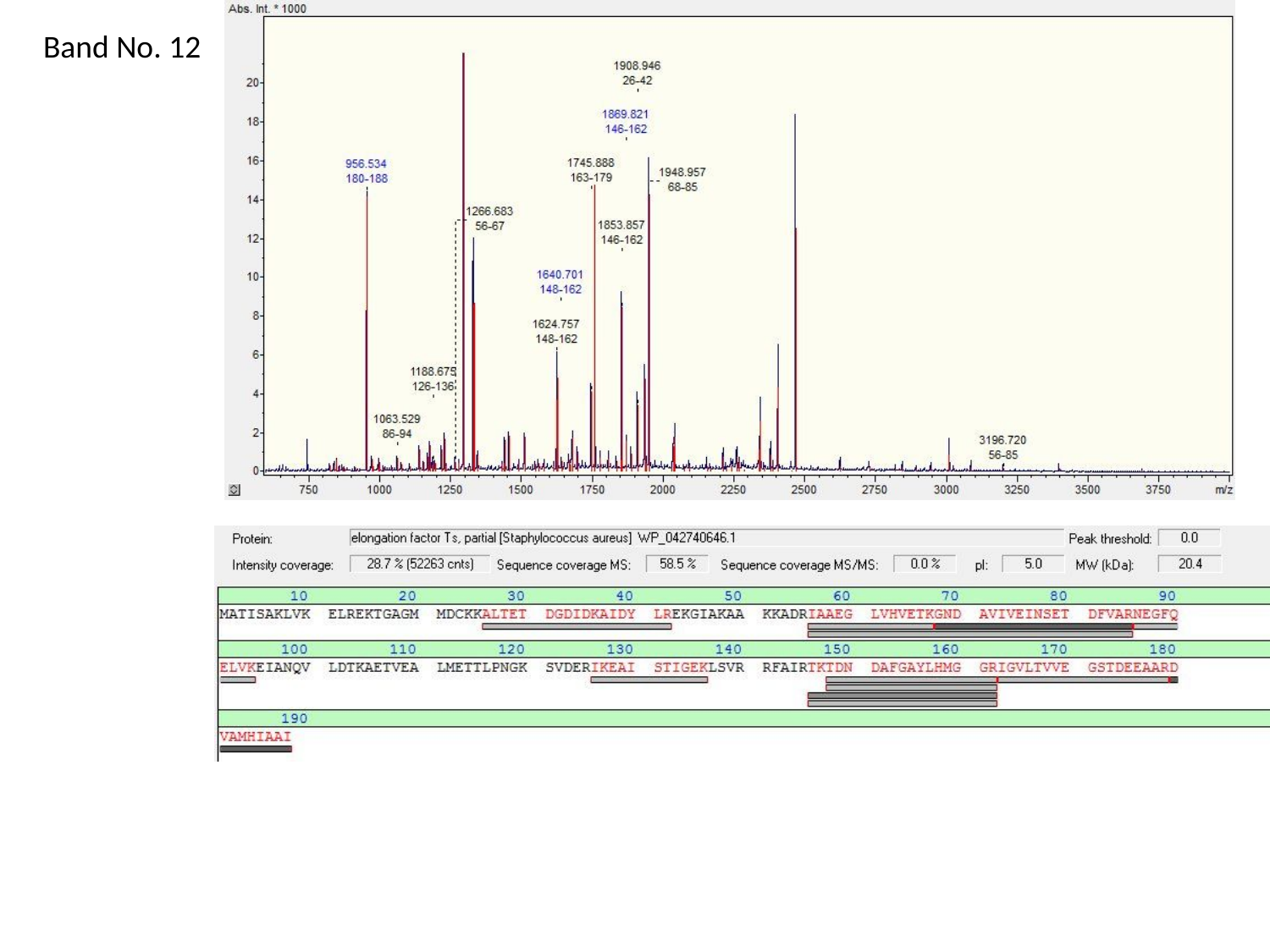

Band No. 12

## Slide 15
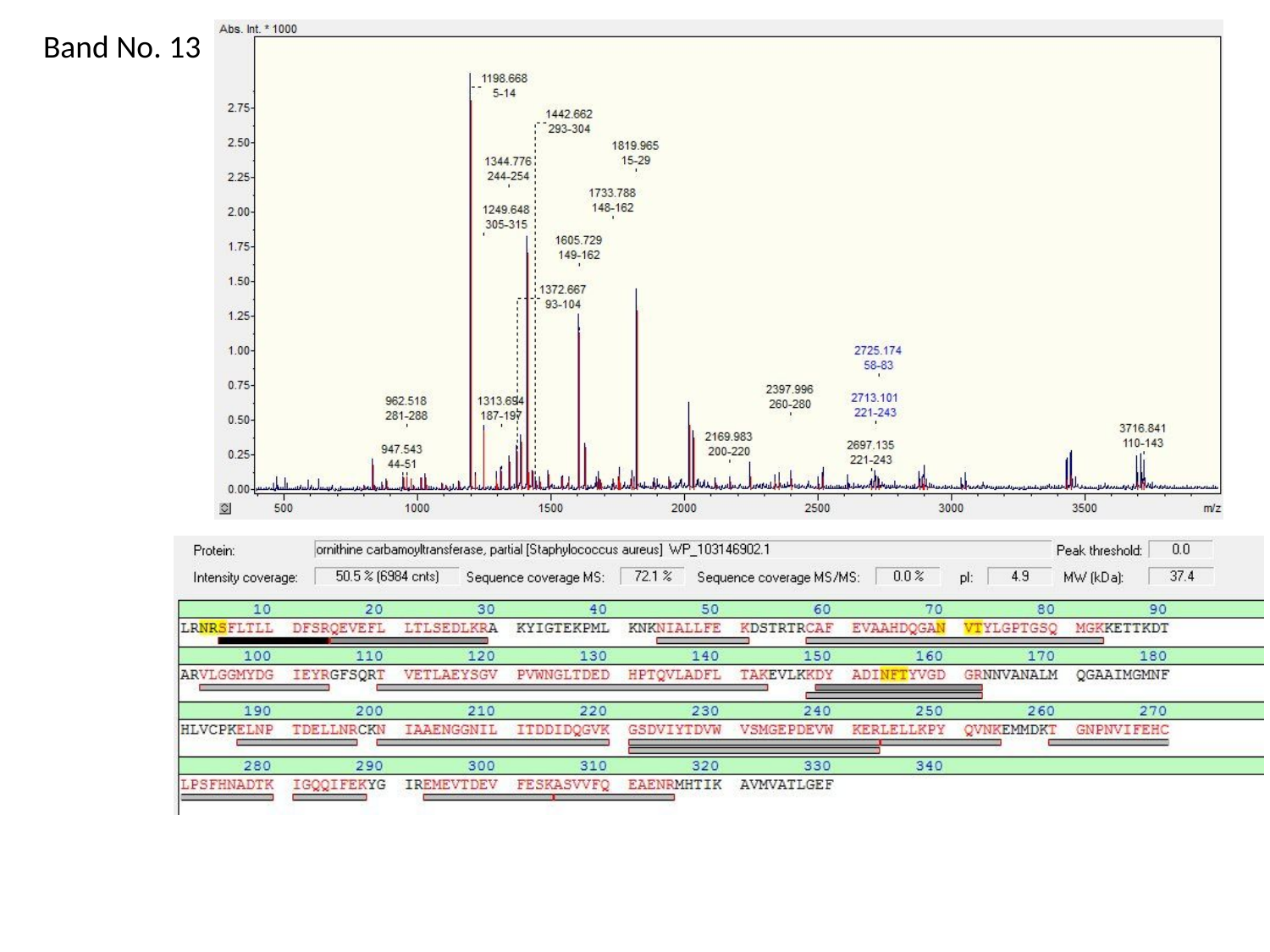

Band No. 13
