# Supplementary material for: Discovery of Furanoquinone Derivatives as a Novel Class of DNA Polymerase and Gyrase Inhibitors for MRSA Eradication in Cutaneous Infection
Source: Front Microbiol. 2019 May 29;10:1197. doi: 10.3389/fmicb.2019.01197 (PMC6549599; doi:10.3389/fmicb.2019.01197)

NaFu-NHOH

Pulse Sequence: s2pul
Solvent: DMSO
Ambient temperature
UNITYplus-400 "unityplus400"

Pulse 42.6 degrees
Acq. time 3.200 sec
Width 6000.6 Hz
64 repetitions
OBSERVE H1, 400.2893014 MHz
DATA PROCESSING
FT size 65536
Total time 3 min, 25 sec

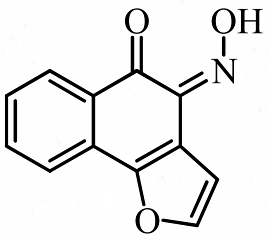

4a

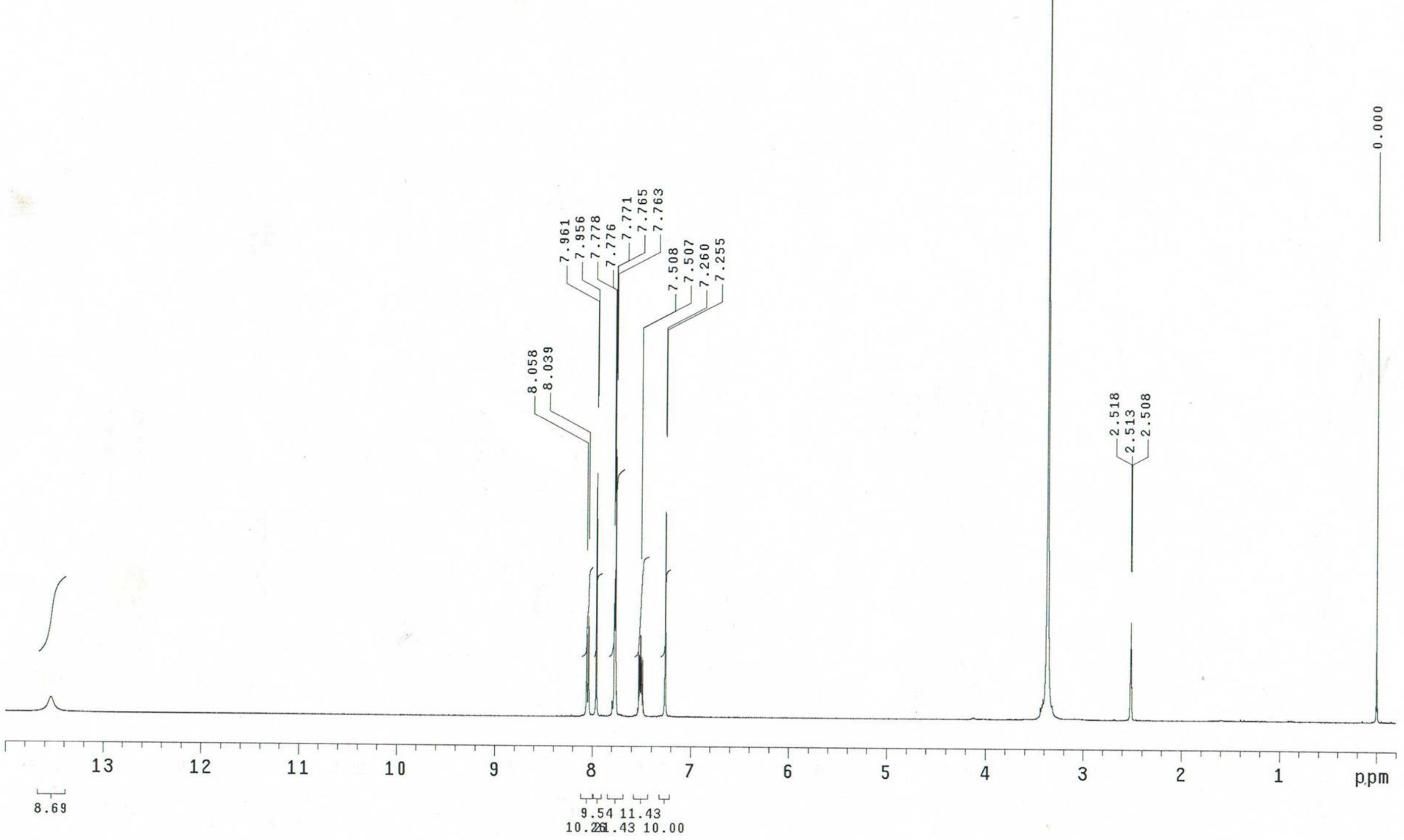

NaFu-NHOH

Pulse Sequence: s2pul

Solvent: DMSO
Ambient temperature
UNITYplus-400 "unityplus400"

Relax. delay 1.000 sec
Pulse 72.3 degrees
Acq. time 1.000 sec
Width 25000.0 Hz
32000 repetitions
OBSERVE C13, 100.6528745 MHz
DECOUPLE H1, 400.2913281 MHz
Power 44 dB
continuously on
WALTZ-16 modulated
DATA PROCESSING
Line broadening 1.0 Hz
FT size 65536
Total time 17 hr, 52 min, 27 sec

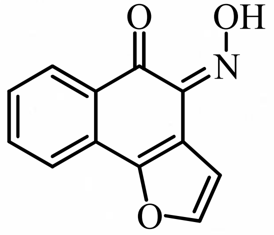

4a

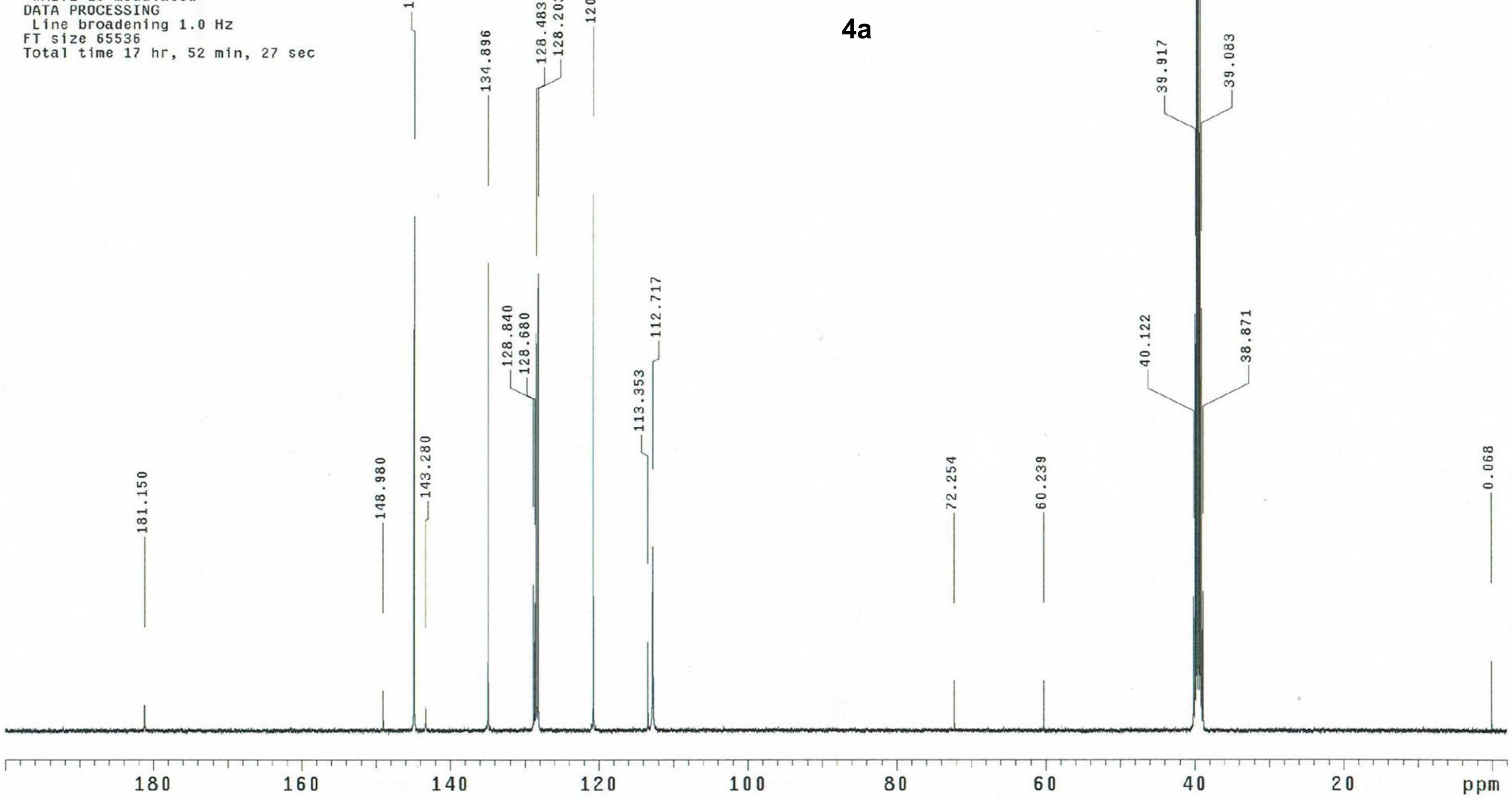

NaFu-NOCH3

Pulse Sequence: s2pul
Solvent: CDCl3
Ambient temperature
UNITYplus-400 "unityplus400"

Pulse 44.8 degrees
Acq. time 3.200 sec
Width 6000.6 Hz
32 repetitions
OBSERVE H1, 400.2874098 MHz
DATA PROCESSING
FT size 65536
Total time 1 min, 42 sec

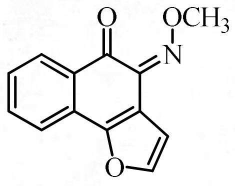

4b

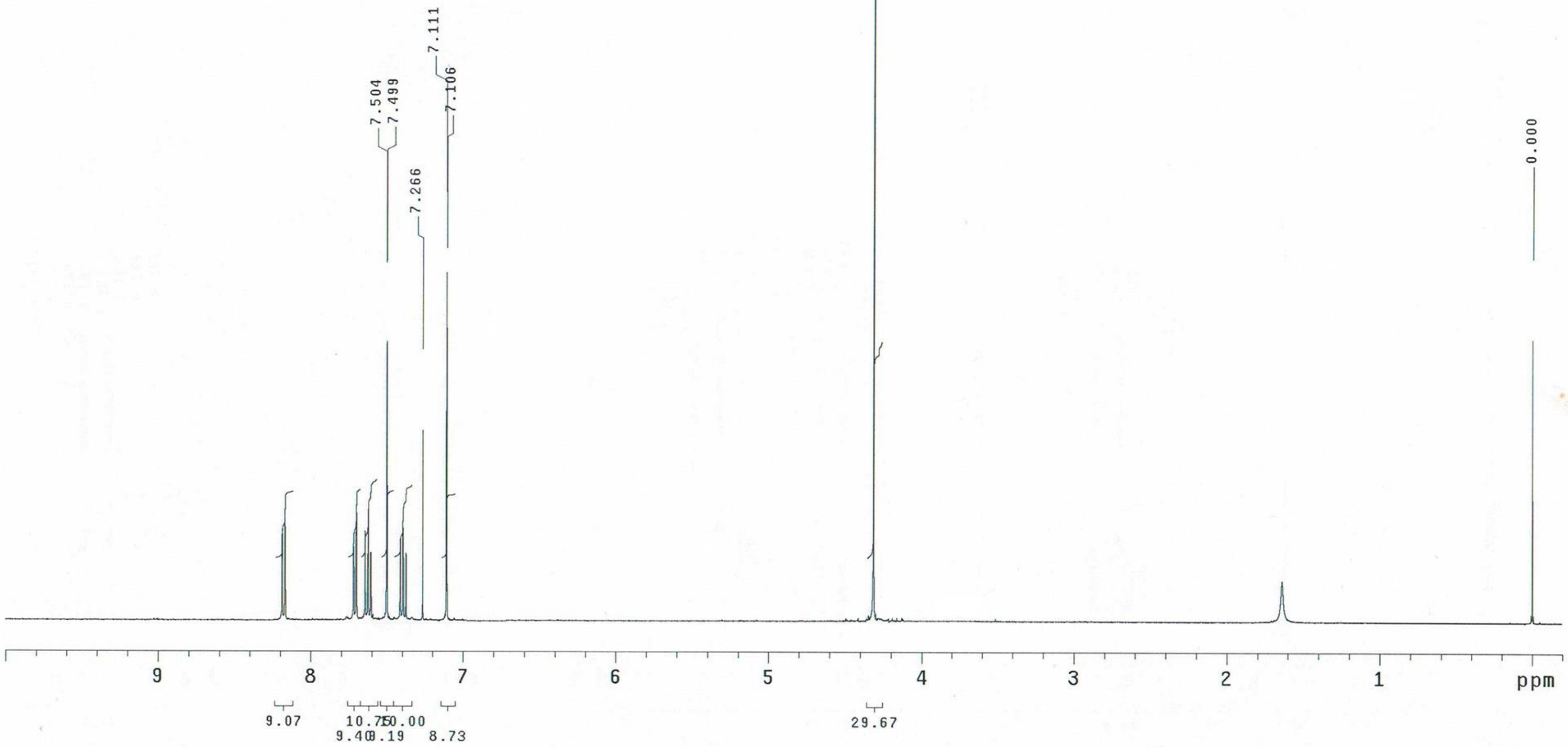

NaFu-NOCH3

Pulse Sequence: s2pul
Solvent: CDCl3
Ambient temperature
UNITYplus-400 "unityplus400"

Relax. delay 1.000 sec
Pulse 65.3 degrees
Acq. time 1.000 sec
Width 25000.0 Hz
22608 repetitions
OBSERVE C13, 100.6523512 MHz
DECOUPLE H1, 400.2894267 MHz
Power 44 dB
continuously on
WALTZ-16 modulated
DATA PROCESSING
Line broadening 1.0 Hz
FT size 65536
Total time 35 hr, 44 min, 54 sec

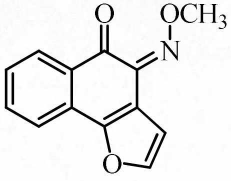

4b

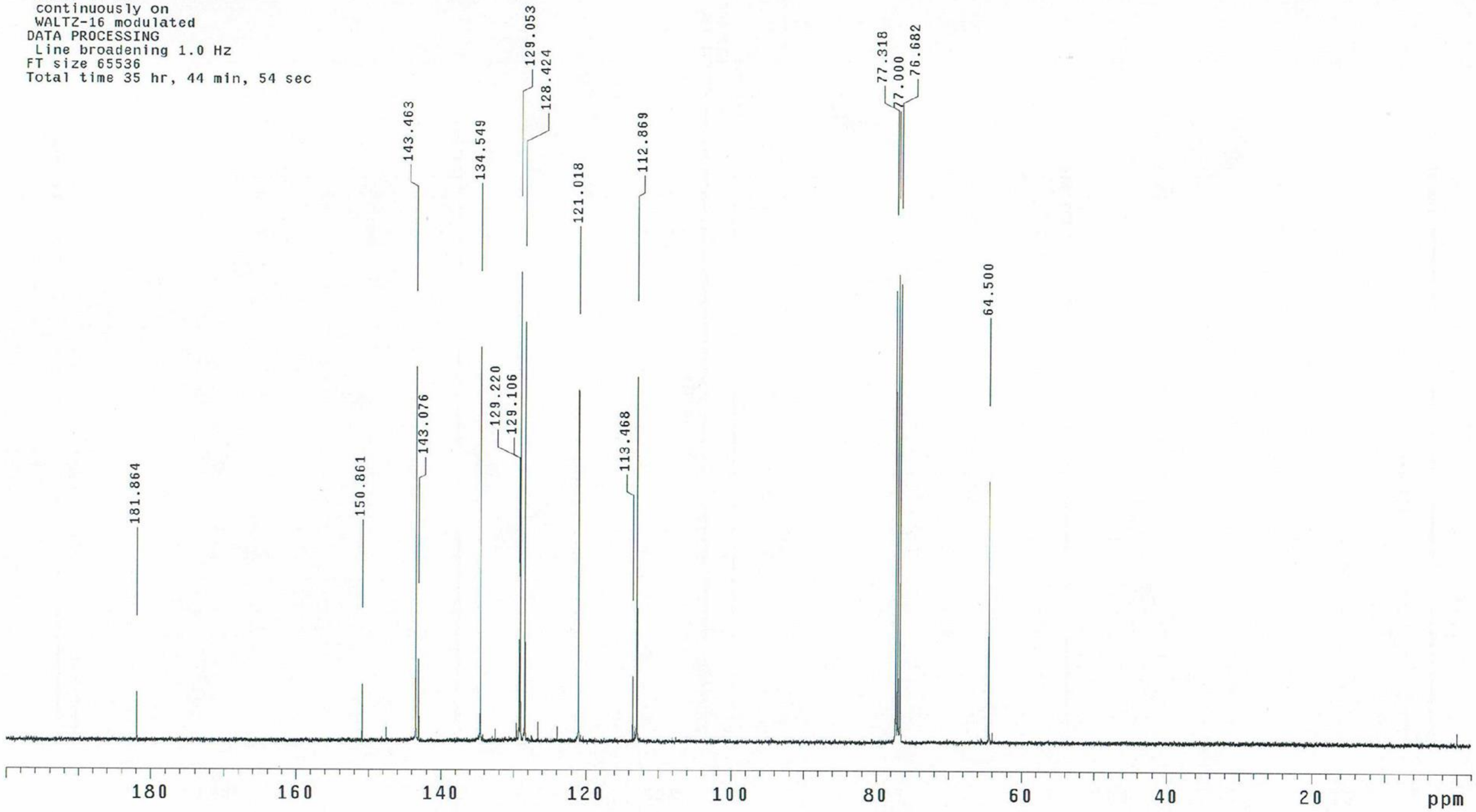

NaFu-O-Benzyl

Pulse Sequence: s2pul

Solvent: CDCl3
Ambient temperature
Mercury-400BB "Mercuryplus400"

Pulse 44.2 degrees
Acq. time 3.000 sec
Width 6006.0 Hz
16 repetitions
OBSERVE H1, 400.4046827 MHz
DATA PROCESSING
FT size 65536
Total time 0 min, 57 sec

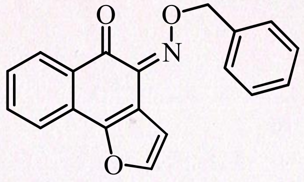

4c

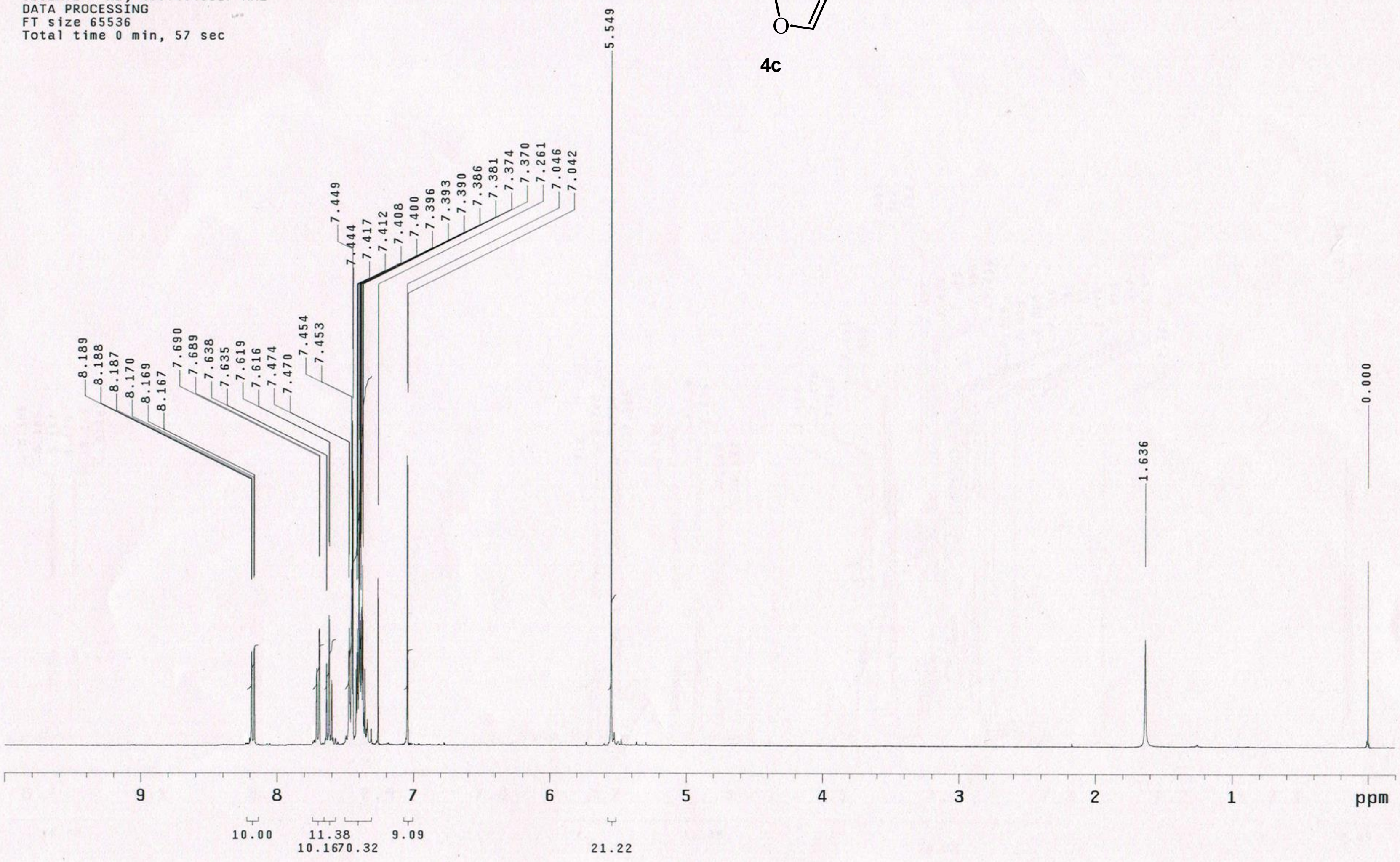

NaFu-O-Benzyl

Pulse Sequence: s2pul

Solvent: CDCl3
Ambient temperature
Mercury-400BB "Mercuryplus400"

Pulse 60.0 degrees
Acq. time 1.000 sec
Width 25000.0 Hz
1032 repetitions
OBSERVE C13, 100.6818405 MHz
DECOUPLE H1, 400.4066668 MHz
Power 37 dB
continuously on
WALTZ-16 modulated
DATA PROCESSING
Line broadening 1.0 Hz
FT size 65536
Total time 12 hr, 41 min, 47 sec

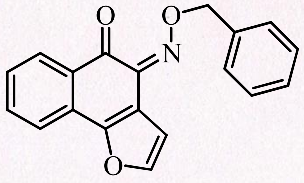

4c

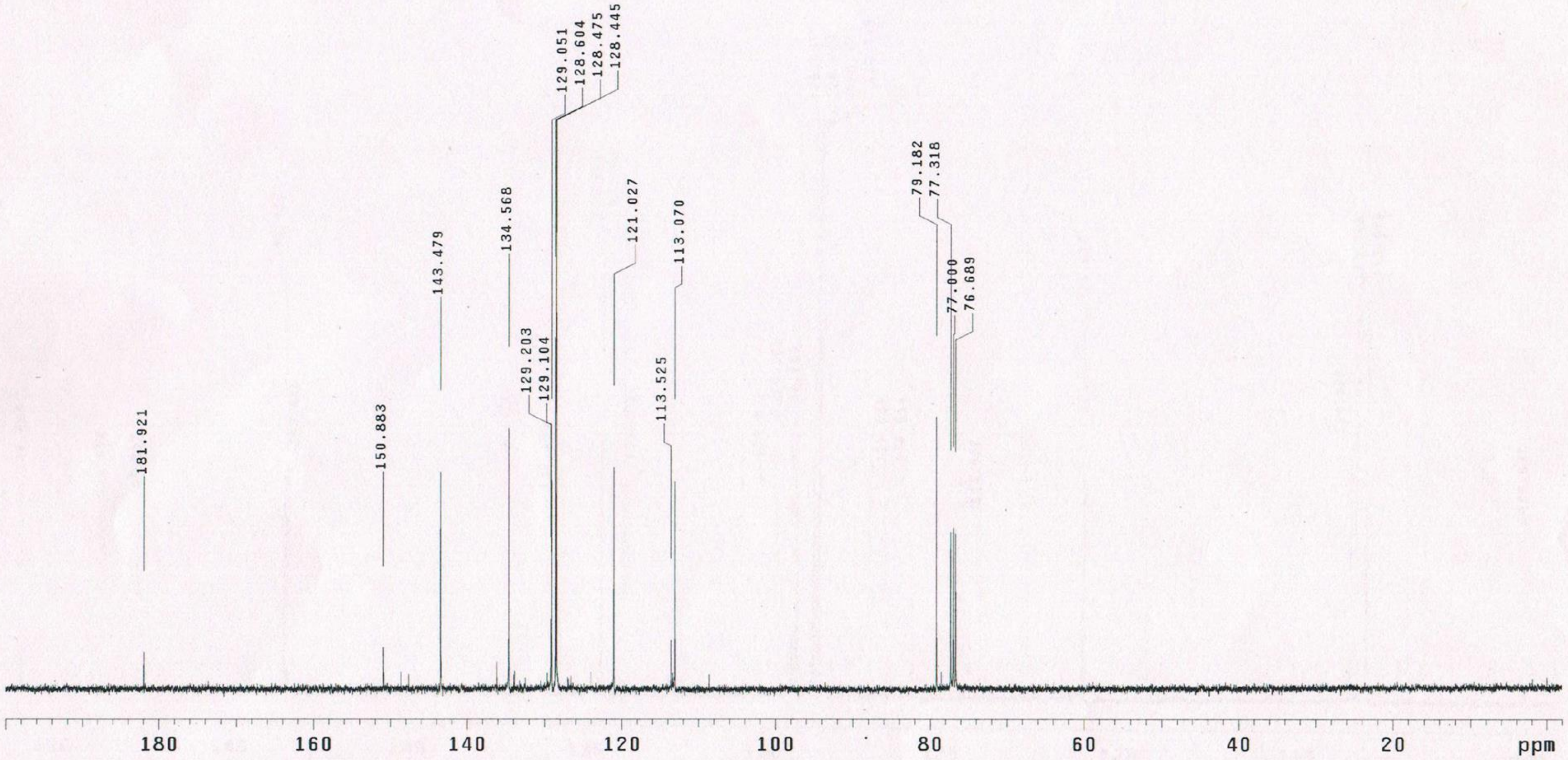

2

Pulse Sequence: s2pul
Solvent: DMSO
Ambient temperature
Mercury-400BB "Mercuryplus400"

Pulse 44.2 degrees
Acq. time 3.000 sec
Width 6006.0 Hz
32 repetitions
OBSERVE H1, 400.4065731 MHz
DATA PROCESSING
FT size 65536
Total time 1 min, 55 sec

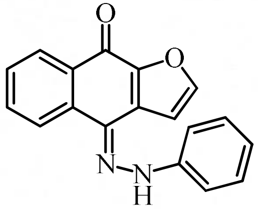

5h

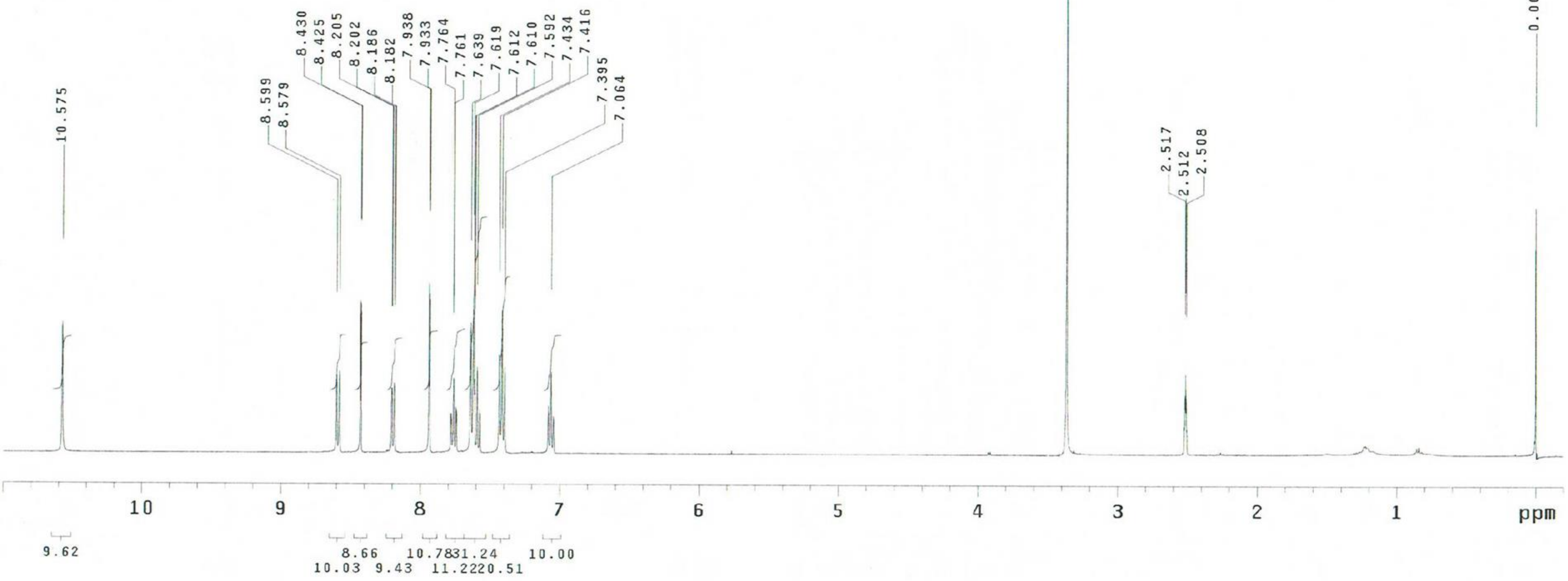

2

Pulse Sequence: s2pul
Solvent: DMSO
Ambient temperature
Mercury-400BB "Mercuryplus400"

Pulse 60.0 degrees
Acq. time 1.000 sec
Width 25000.0 Hz
2176 repetitions
OBSERVE C13, 100.6823612 MHz
DECOUPLE H1, 400.4085687 MHz
Power 37 dB
continuously on
WALTZ-16 modulated
DATA PROCESSING
Line broadening 1.0 Hz
FT size 65536
Total time 12 hr, 41 min, 47 sec

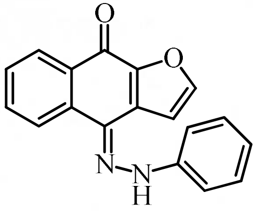

5h

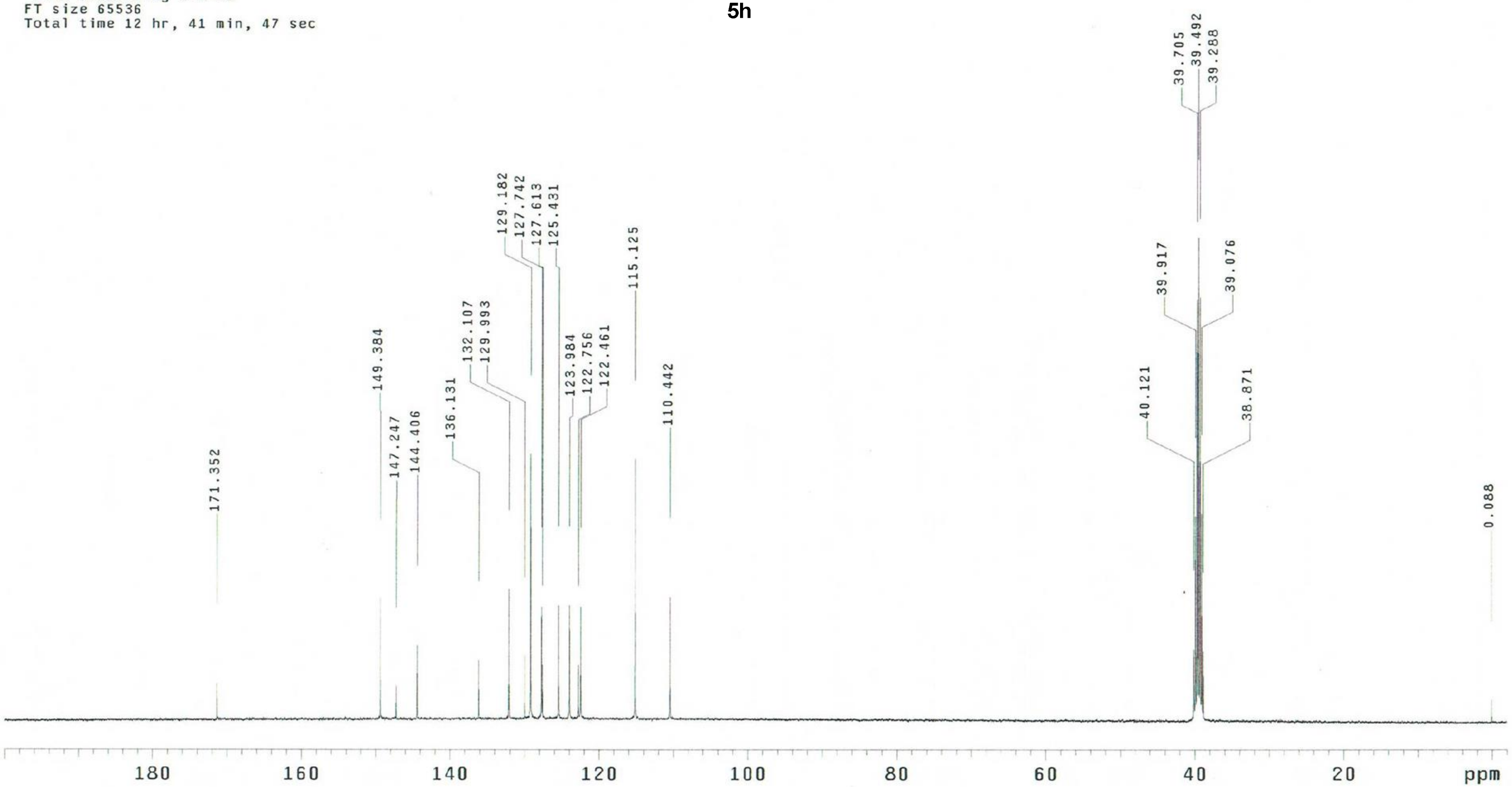

LiNaFu-F

Pulse Sequence: s2pul
UNITYplus-400 "unity400"
Date: Feb 22 2018
Solvent: DMSO
Ambient temperature
Total 32 repetitions

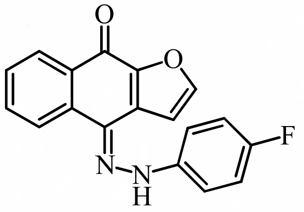

5i

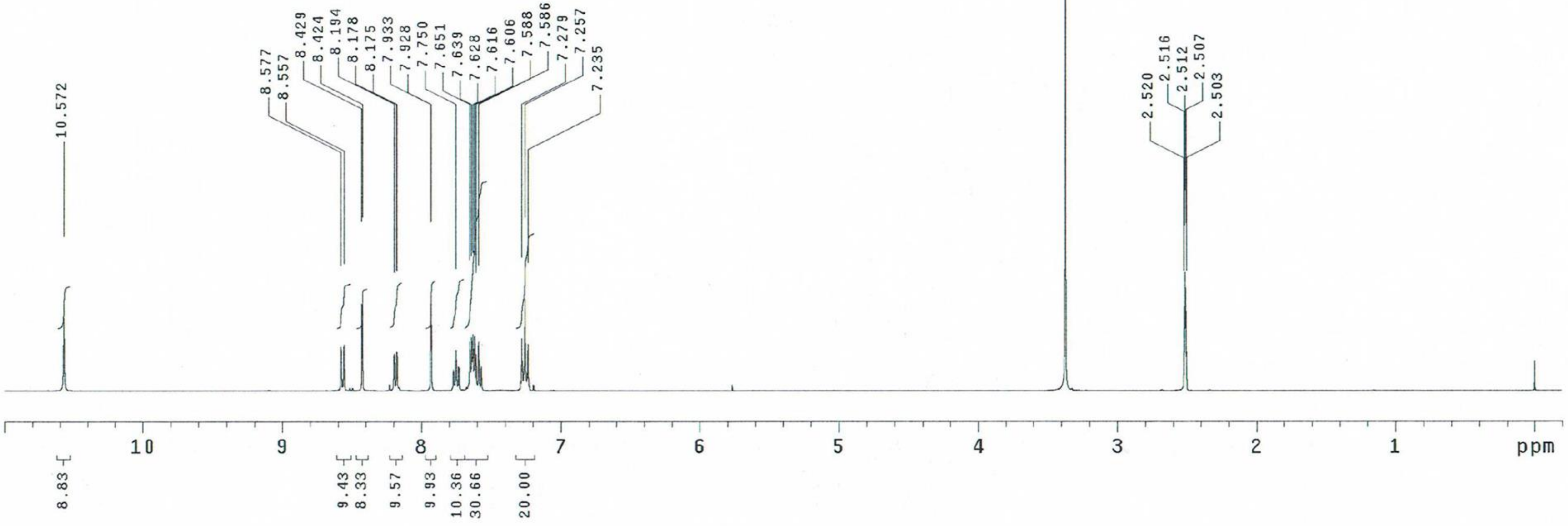

LiNaFu-F

Pulse Sequence: s2pul
UNITYplus-400 "unity400"
Date: Feb 22 2018
Solvent: CDCl3
Ambient temperature
Total 16000 repetitions

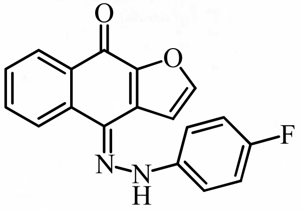

5i

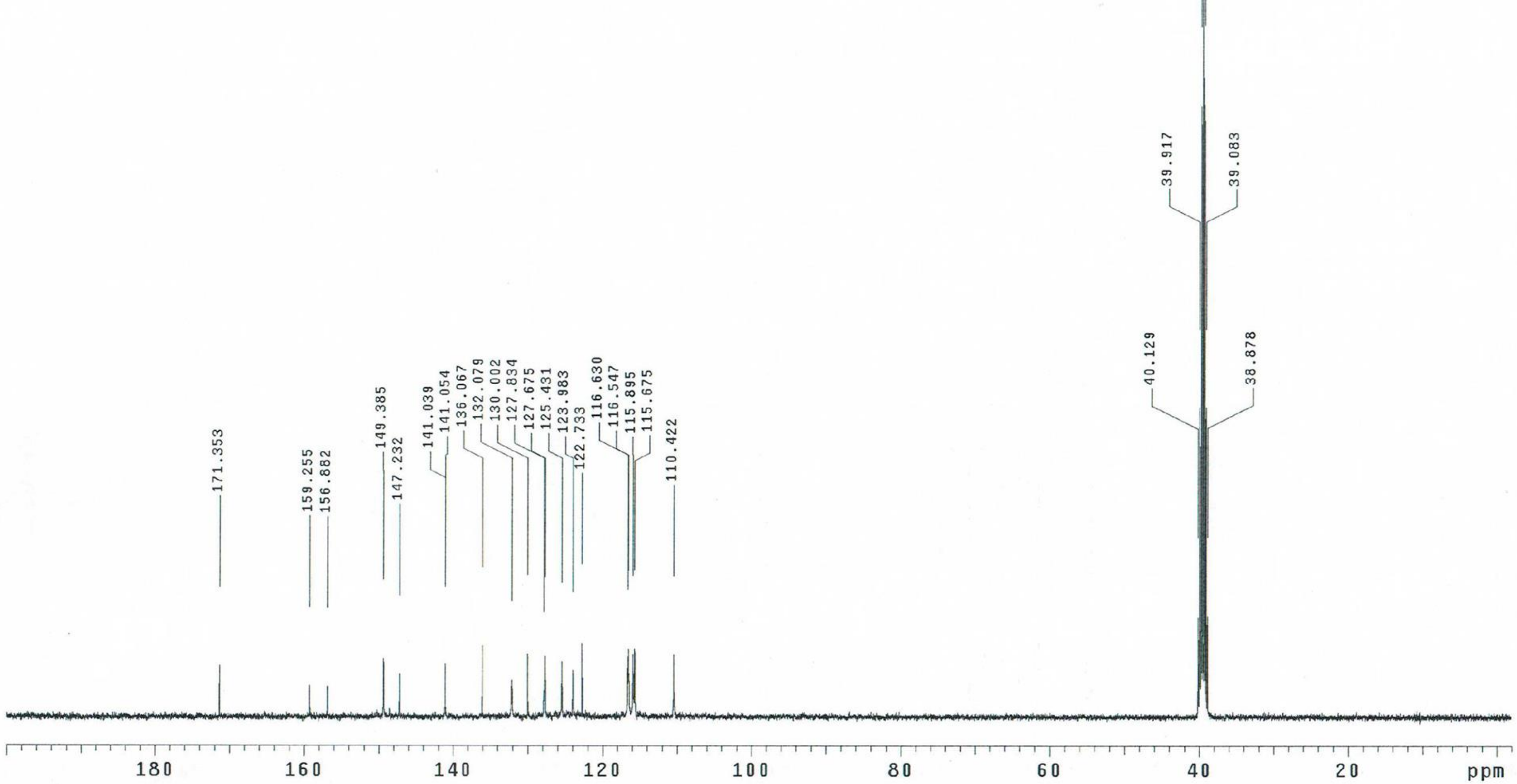

LiNaFu-OMe

Pulse Sequence: s2pul
Mercury-400BB "MerPlus400"
Date: Mar 1 2018
Solvent: dmso
Ambient temperature
Total 68 repetitions

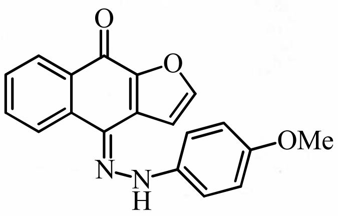

5j

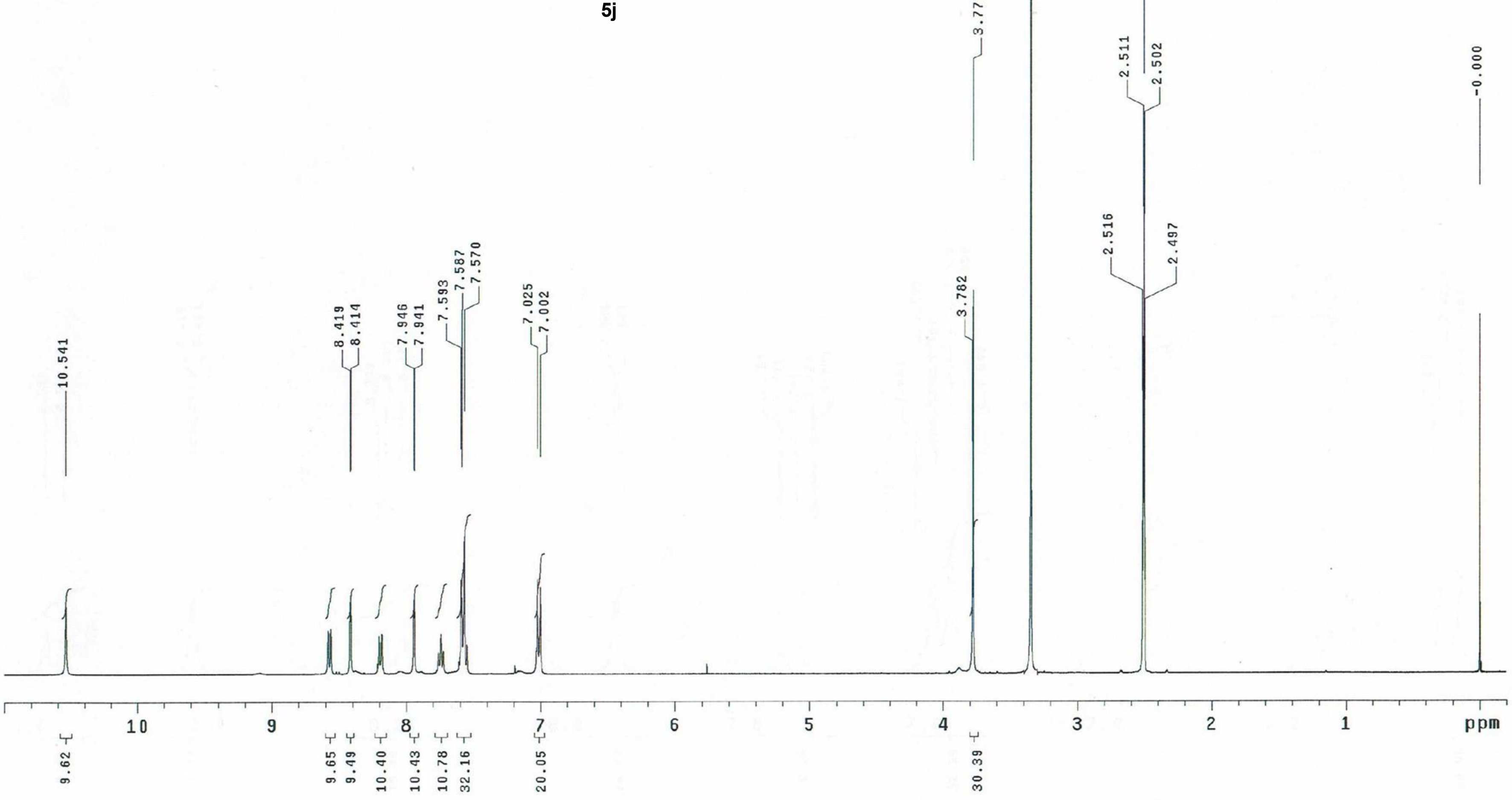

LiNaFu-OMe

Pulse Sequence: s2pul
Mercury-400BB "MerPlus400"
Date: Mar 1 2018
Solvent: dmso
Ambient temperature
Total 10288 repetitions

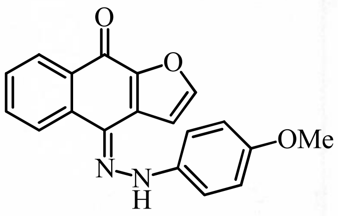

5j

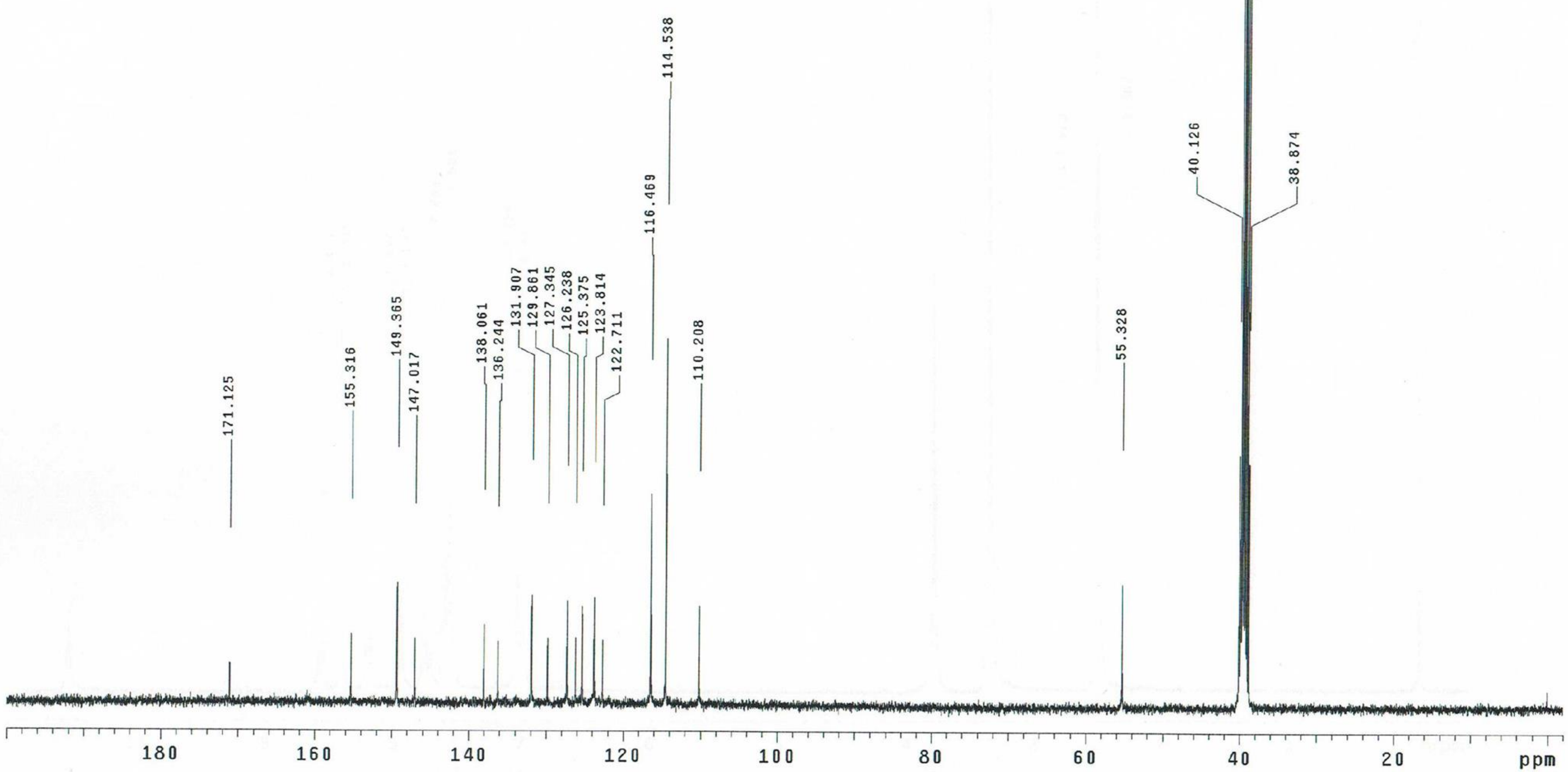

LiNaFu-Me

Pulse Sequence: s2pul
UNITYplus-400 "unity400"
Date: Feb 22 2018
Solvent: DMSO
Ambient temperature
Total 32 repetitions

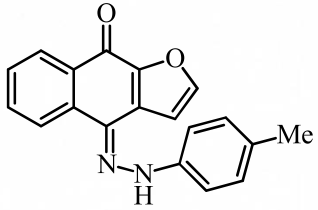

5k

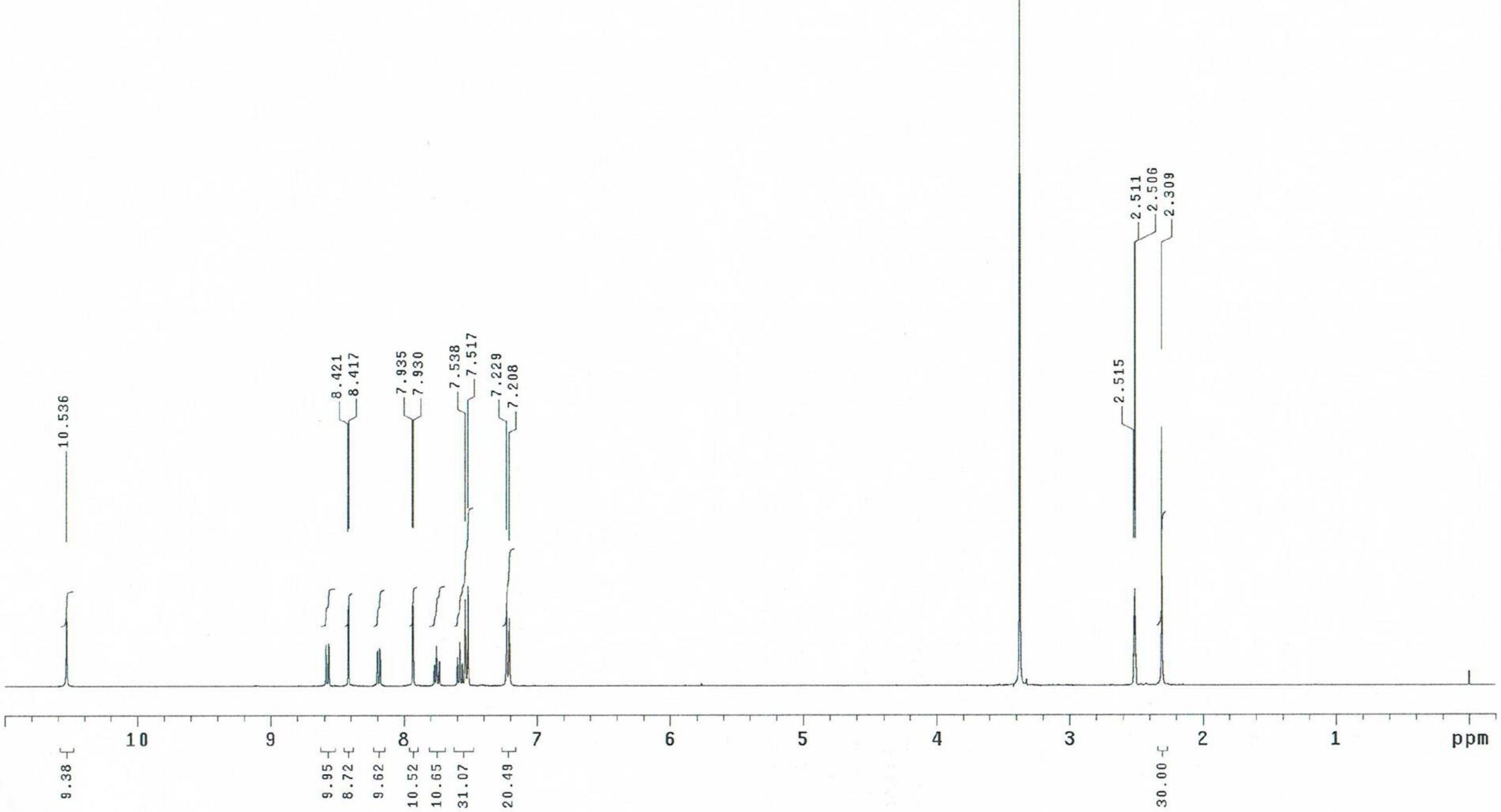

LiNaFu-Me

Pulse Sequence: s2pul

UNITYplus-400 "unity400"
Date: Feb 22 2018
Solvent: DMSO
Ambient temperature
Total 4032 repetitions

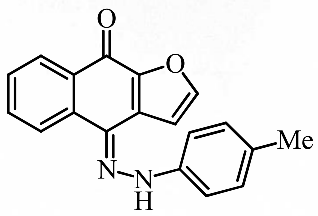

5k

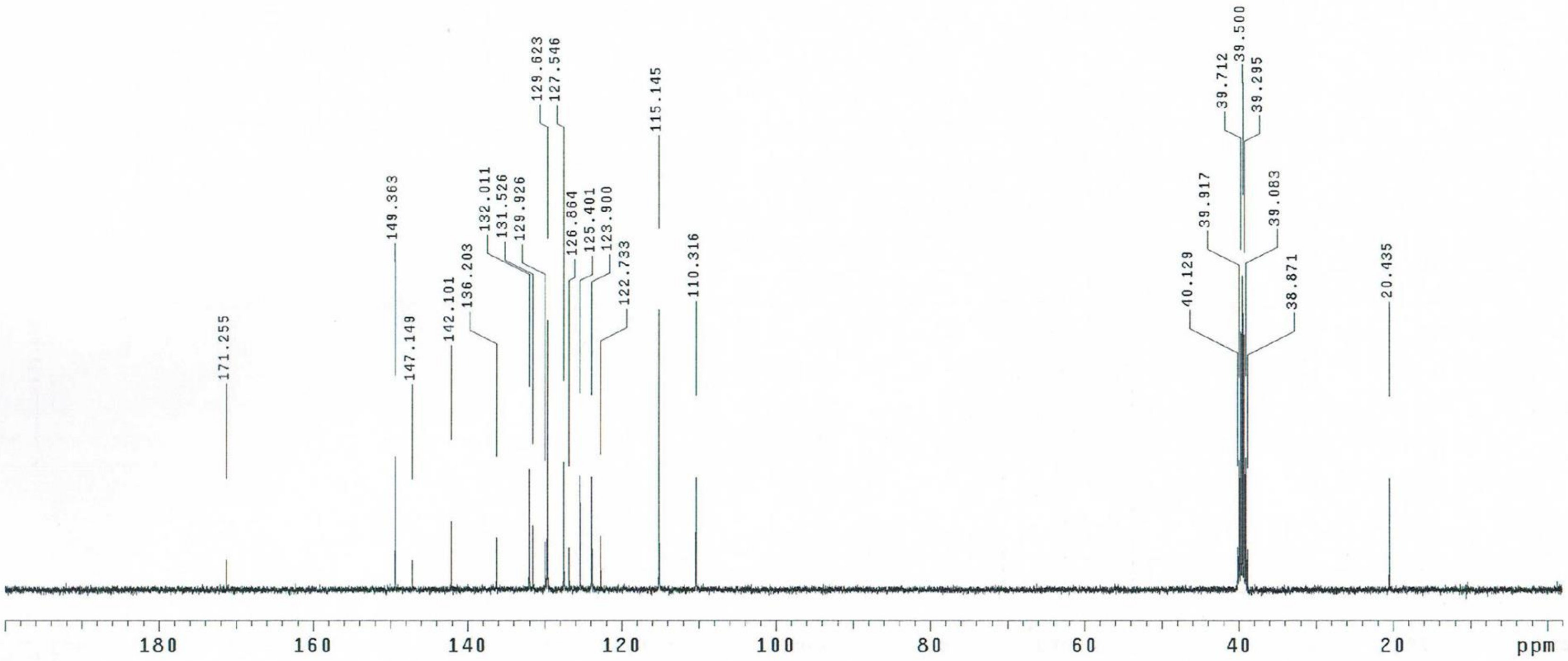

NaFu-NNAC

Pulse Sequence: s2pul

Solvent: DMSO
Ambient temperature
UNITYplus-400 "unityplus400"

Pulse 44.8 degrees
Acq. time 3.200 sec
Width 8000.0 Hz
48 repetitions
OBSERVE H1, 400.2893008 MHz
DATA PROCESSING
FT size 65536
Total time 3 min, 25 sec

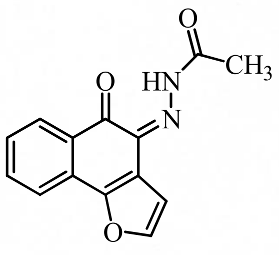

6d

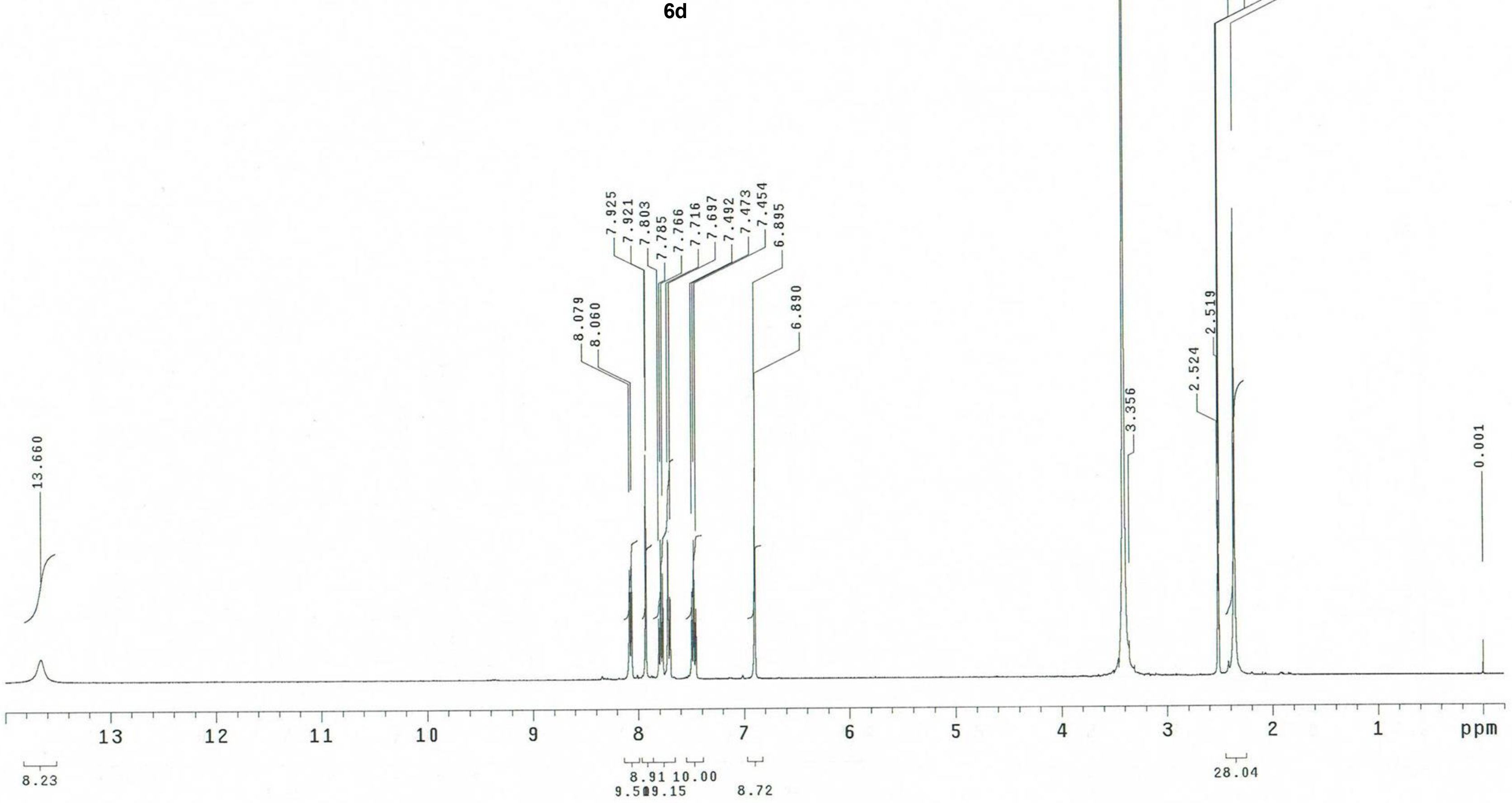

NaFu-NNAC

Pulse Sequence: s2pul

Solvent: DMSO
Ambient temperature
UNITYplus-400 "unityplus400"

Pulse 65.3 degrees
Acq. time 1.000 sec
Width 25000.0 Hz
8528 repetitions
OBSERVE C13, 100.6528692 MHz
DECOUPLE H1, 400.2913281 MHz
Power 44 dB
continuously on
WALTZ-16 modulated
DATA PROCESSING
Line broadening 1.0 Hz
FT size 65536
Total time 5 hr, 36 min, 56 sec

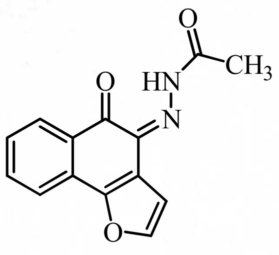

6d

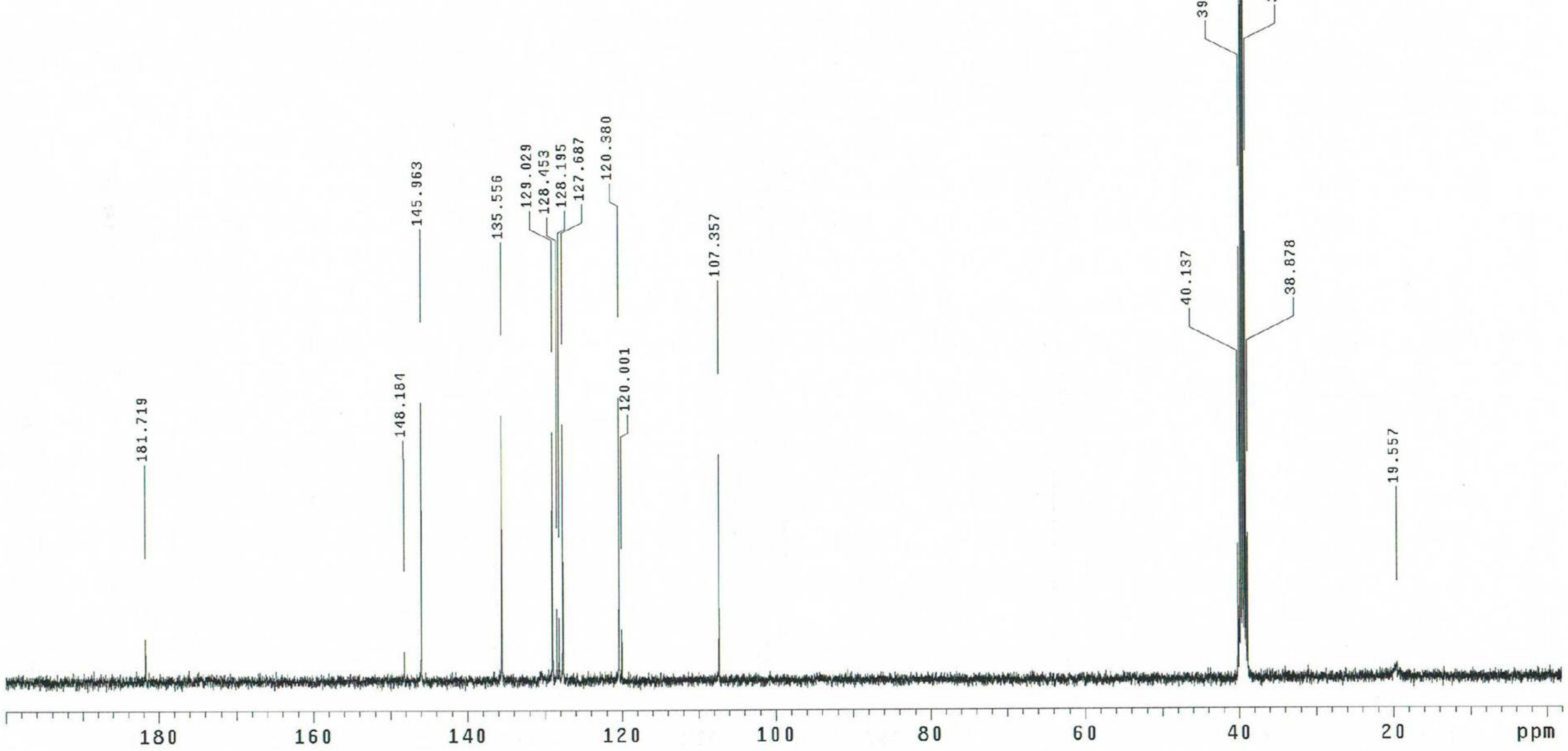

NaFu-Semicar

Pulse Sequence: s2pul

Solvent: DMSO
Ambient temperature
UNITYplus-400 "unityplus400"

Pulse 44.8 degrees
Acq. time 3.200 sec
Width 8000.0 Hz
32 repetitions
OBSERVE H1, 400.2893015 MHz
DATA PROCESSING
FT size 65536
Total time 1 min, 42 sec

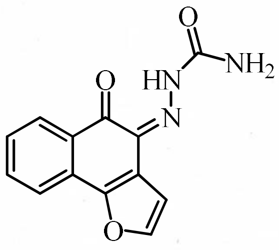

6e

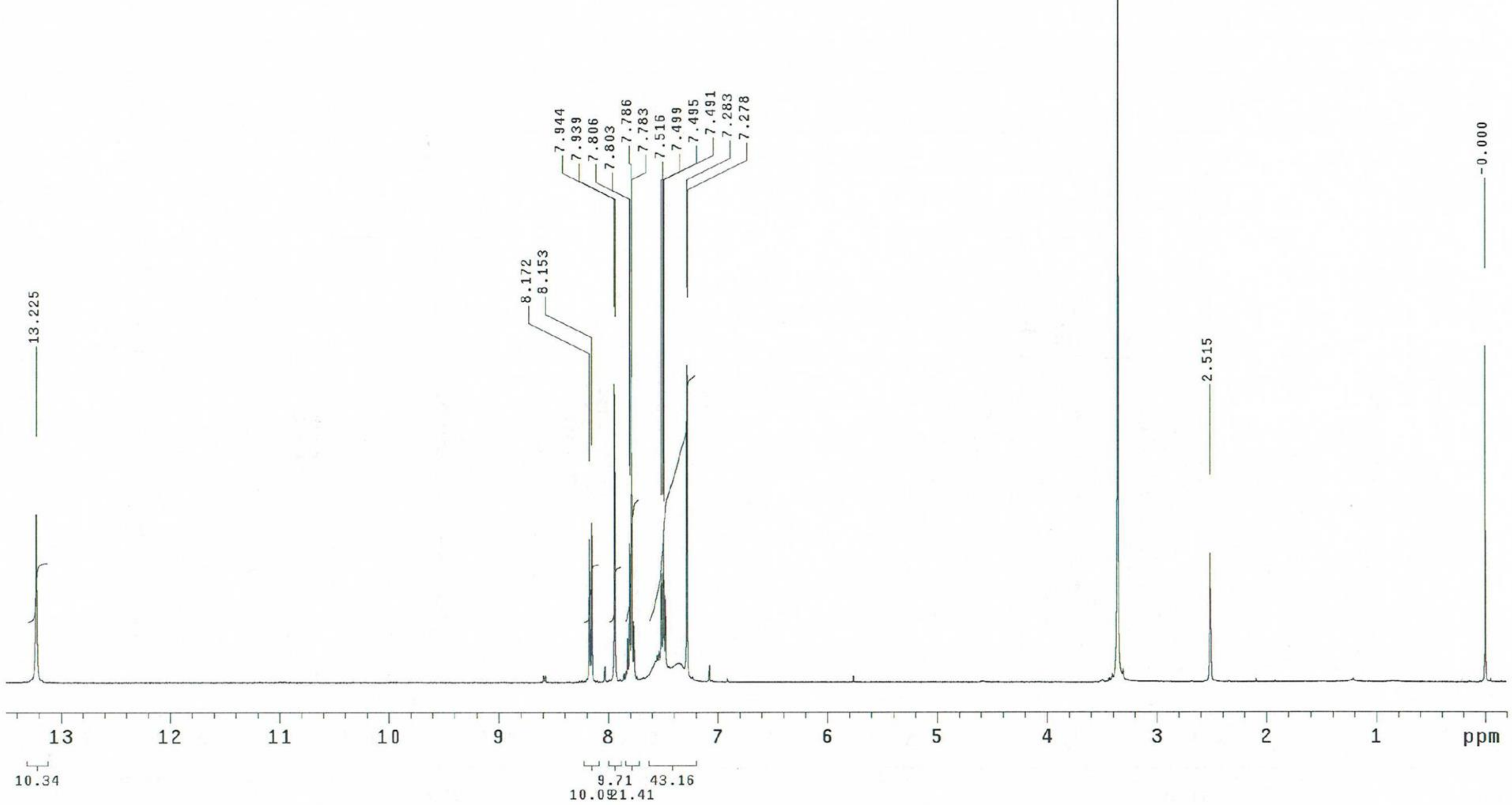

NaFu-Semicar

Pulse Sequence: s2pul

Solvent: DMSO
Ambient temperature
UNITYplus-400 "unityplus400"

Pulse 65.3 degrees
Acq. time 1.000 sec
Width 25000.0 Hz
1072 repetitions
OBSERVE C13, 100.6528745 MHz
DECOUPLE H1, 400.2913281 MHz
Power 44 dB
continuously on
WALTZ-16 modulated
DATA PROCESSING
Line broadening 1.0 Hz
FT size 65536
Total time 5 hr, 36 min, 56 sec

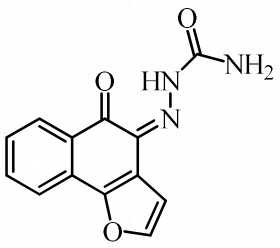

6e

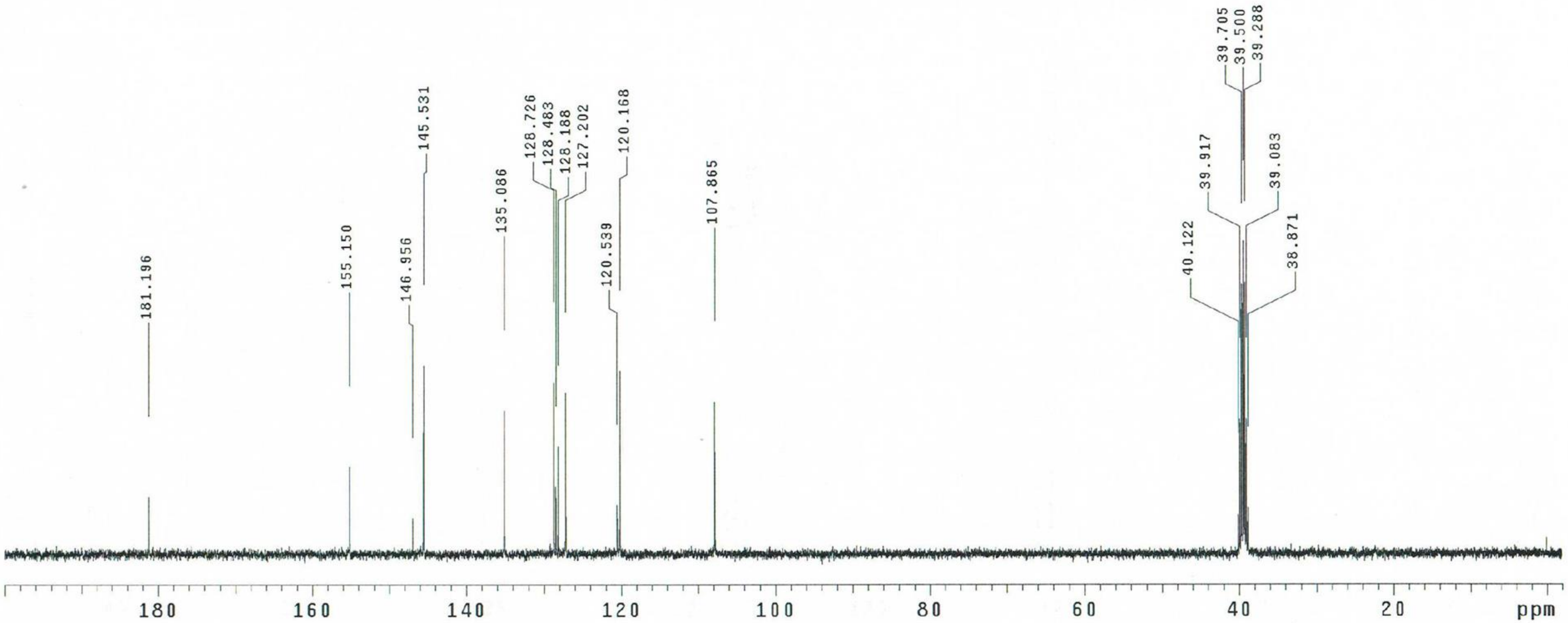

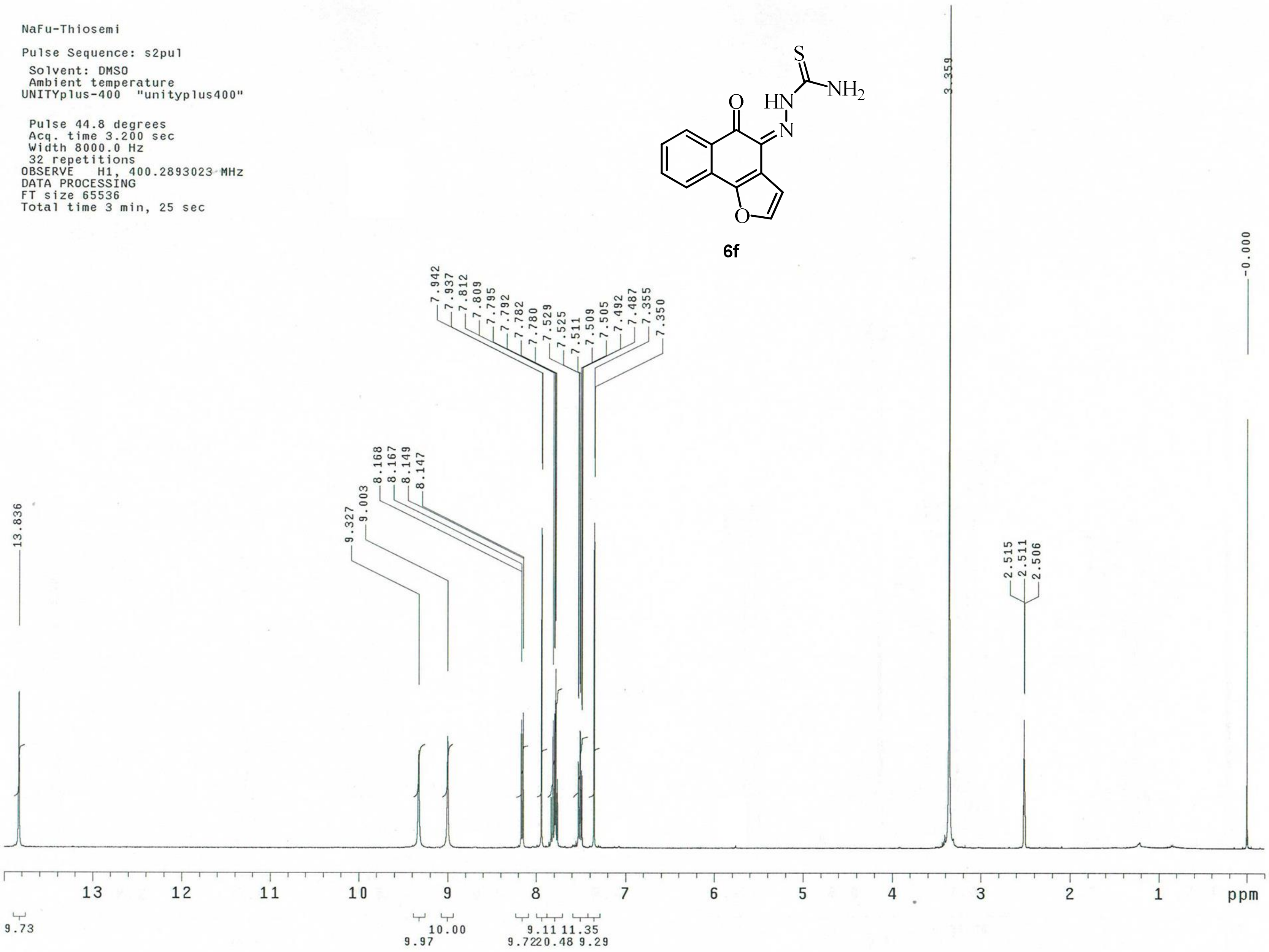
NaFu-Thiosemi
Pulse Sequence: s2pul
Solvent: DMSO
Ambient temperature
UNITYplus-400 "unityplus400"
Pulse 44.8 degrees
Acq. time 3.200 sec
Width 8000.0 Hz
32 repetitions
OBSERVE H1, 400.2893023 MHz
DATA PROCESSING
FT size 65536
Total time 3 min, 25 sec
S
NH2
HN
O
N
O
6f
13.836
9.327
9.003
8.168
8.167
8.149
8.147
7.942
7.937
7.812
7.809
7.795
7.792
7.782
7.780
7.529
7.525
7.511
7.509
7.505
7.492
7.487
7.355
7.350
3.359
2.515
2.511
2.506
-0.000
13
12
11
10
9
8
7
6
5
4
3
2
1
ppm
9.73
9.97
10.00
9.72
20.48
9.11
11.35
9.29

NaFu-Thiosemi

Pulse Sequence: s2pul

Solvent: DMSO
Ambient temperature
UNITYplus-400 "unityplus400"

Pulse 65.3 degrees
Acq. time 1.000 sec
Width 25000.0 Hz
2048 repetitions
OBSERVE C13, 100.6528737 MHz
DECOUPLE H1, 400.2913281 MHz
Power 44 dB
continuously on
WALTZ-16 modulated
DATA PROCESSING
Line broadening 1.0 Hz
FT size 65536
Total time 5 hr, 36 min, 56 sec

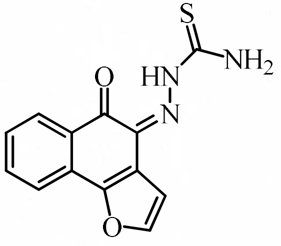

6f

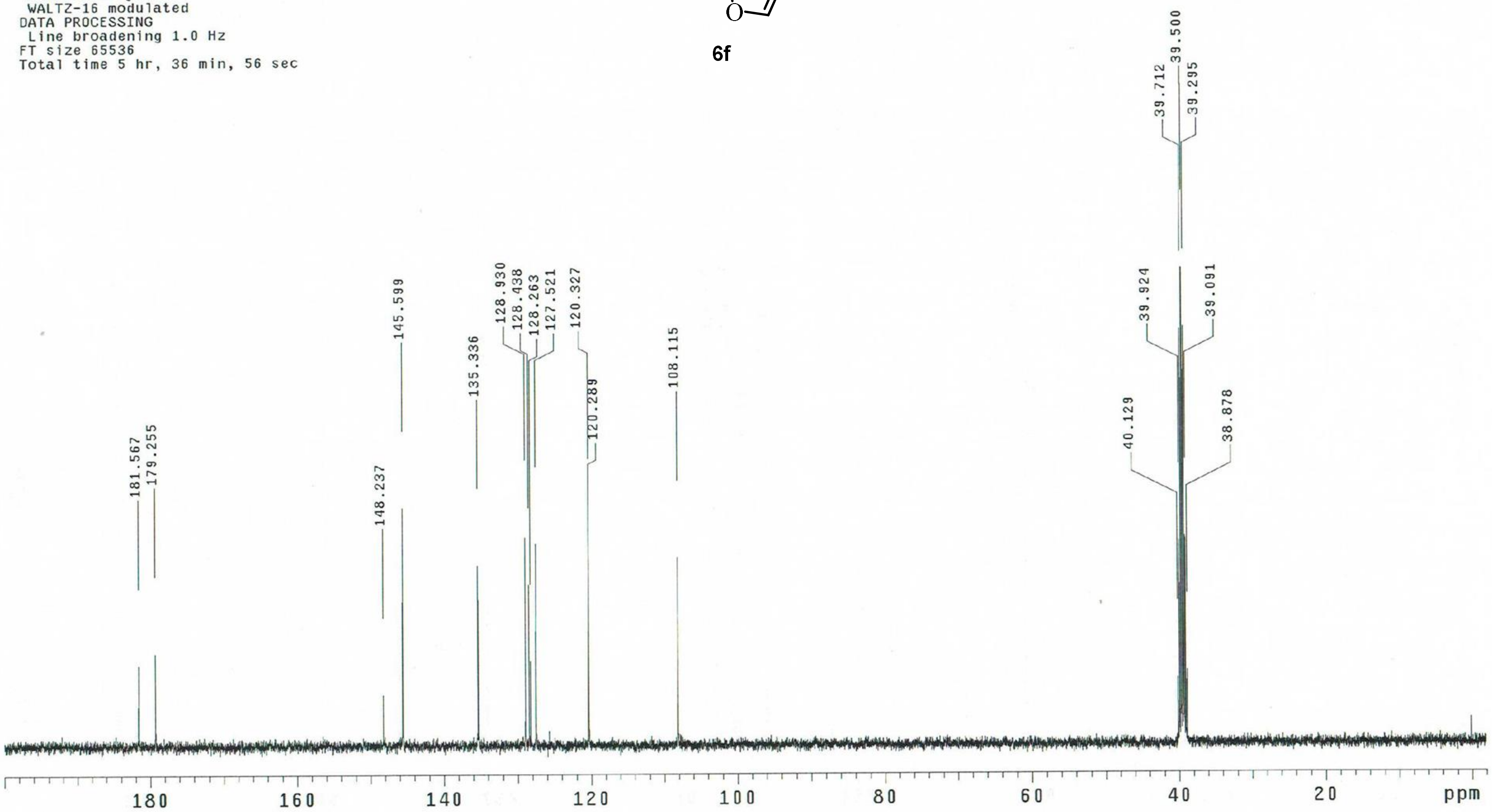

NaFu-4-CH3-thiosemi

Pulse Sequence: s2pul
Solvent: DMSO
Ambient temperature
UNITYplus-400 "unityplus400"

Pulse 44.8 degrees
Acq. time 3.200 sec
Width 8000.0 Hz
40 repetitions
OBSERVE H1, 400.2893035 MHz
DATA PROCESSING
FT size 65536
Total time 2 hr, 51 min, 13 sec

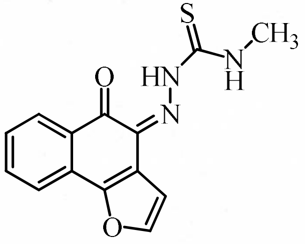

6g

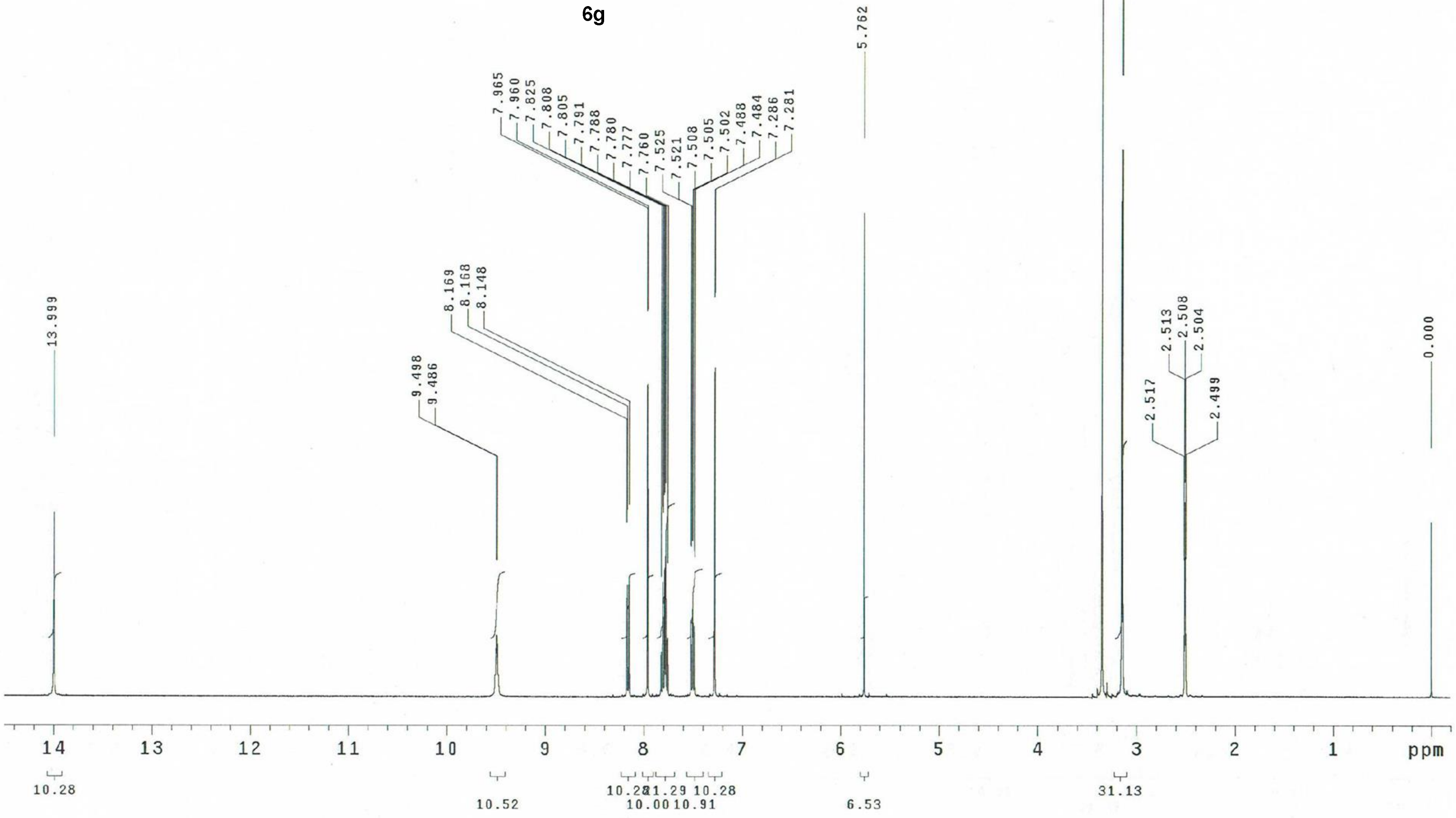

NaFu-4-CH3-thiosemi

Pulse Sequence: s2pul

Solvent: DMSO
Ambient temperature
UNITYplus-400 "unityplus400"

Pulse 65.3 degrees
Acq. time 1.000 sec
Width 25000.0 Hz
6800 repetitions
OBSERVE C13, 100.6528745 MHz
DECOUPLE H1, 400.2913281 MHz
Power 44 dB
continuously on
WALTZ-16 modulated
DATA PROCESSING
Line broadening 1.0 Hz
FT size 65536
Total time 5 hr, 36 min, 56 sec

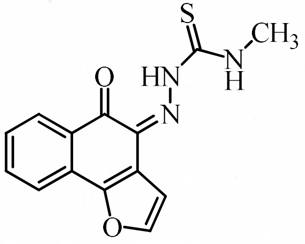

6g

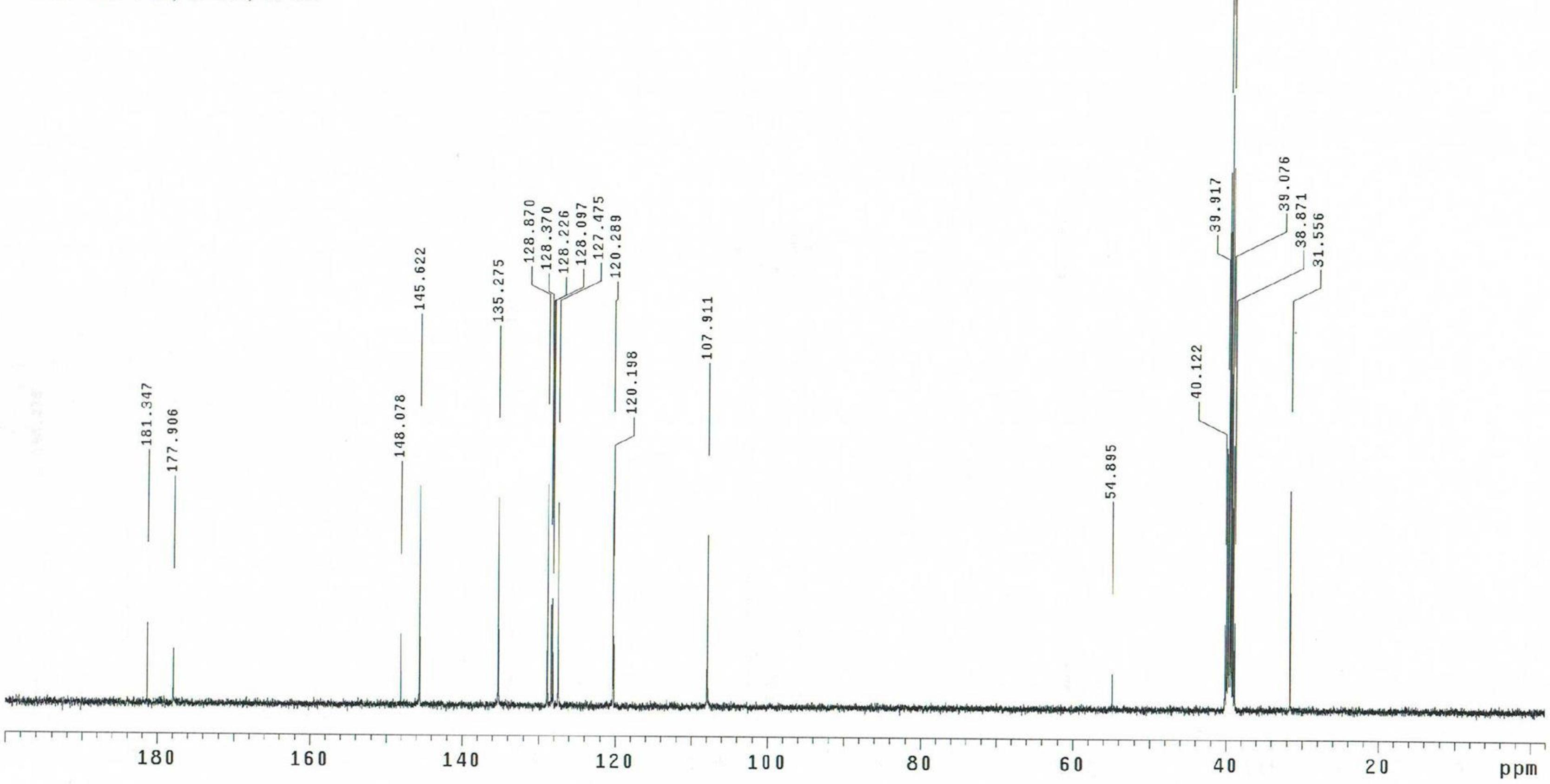

NaFu-NNPh

Pulse Sequence: s2pul
Solvent: CDCl3
Ambient temperature
UNITYplus-400 "unityplus400"

Pulse 44.8 degrees
Acq. time 3.200 sec
Width 8000.0 Hz
64 repetitions
OBSERVE H1, 400.2874131 MHz
DATA PROCESSING
FT size 65536
Total time 3 min, 25 sec

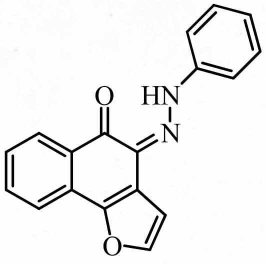

6h

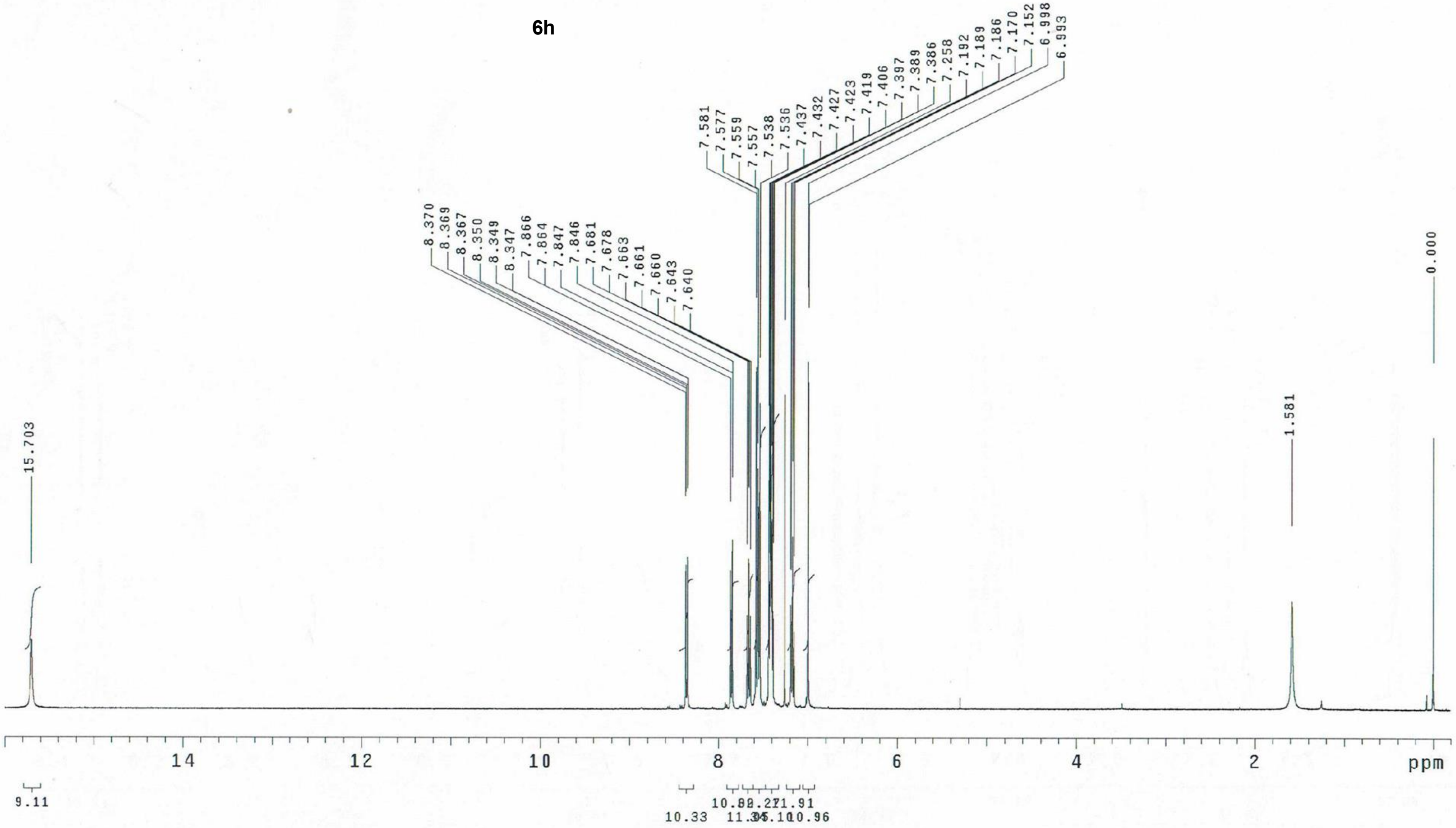

NaFu-NNPh

Pulse Sequence: s2pul
Solvent: CDC13
Ambient temperature
UNITYplus-400 "unityplus400"

Pulse 65.3 degrees
Acq. time 1.000 sec
Width 25000.0 Hz
4832 repetitions
OBSERVE C13, 100.6523505 MHz
DECOUPLE H1, 400.2894267 MHz
Power 44 dB
continuously on
WALTZ-16 modulated
DATA PROCESSING
Line broadening 1.0 Hz
FT size 65536
Total time 5 hr, 36 min, 56 sec

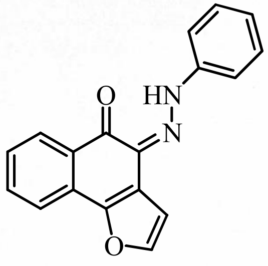

6h

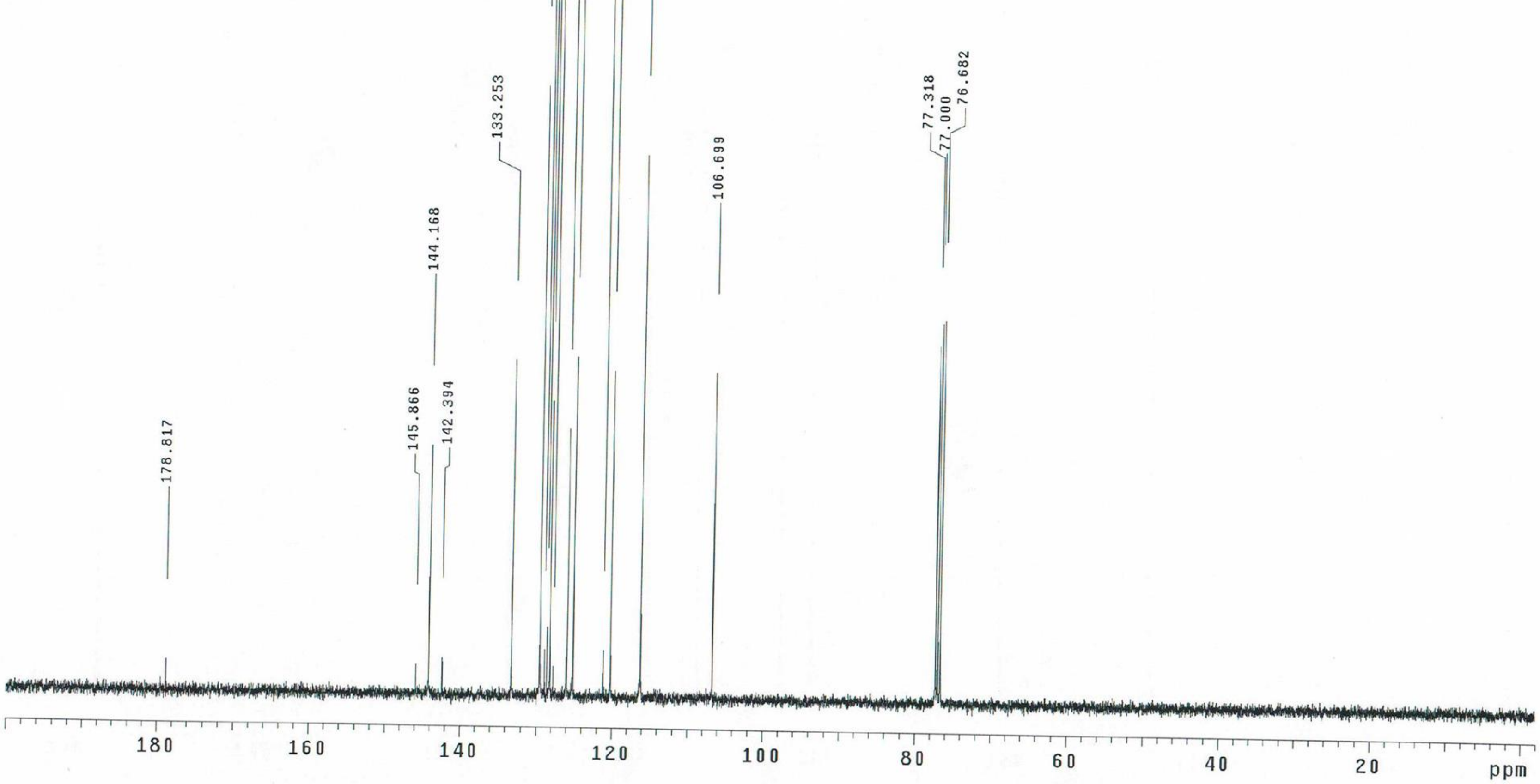

NaFu-NN4FPh

Pulse Sequence: s2pul
Solvent: CDCl3
Ambient temperature
UNITYplus-400 "unityplus400"

Pulse 44.8 degrees
Acq. time 3.200 sec
Width 8000.0 Hz
16 repetitions
OBSERVE H1, 400.2874128 MHz
DATA PROCESSING
FT size 65536
Total time 0 min, 51 sec

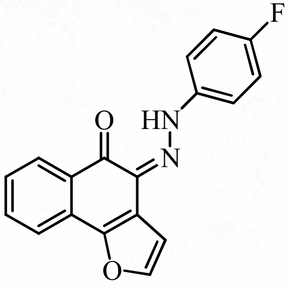

6i

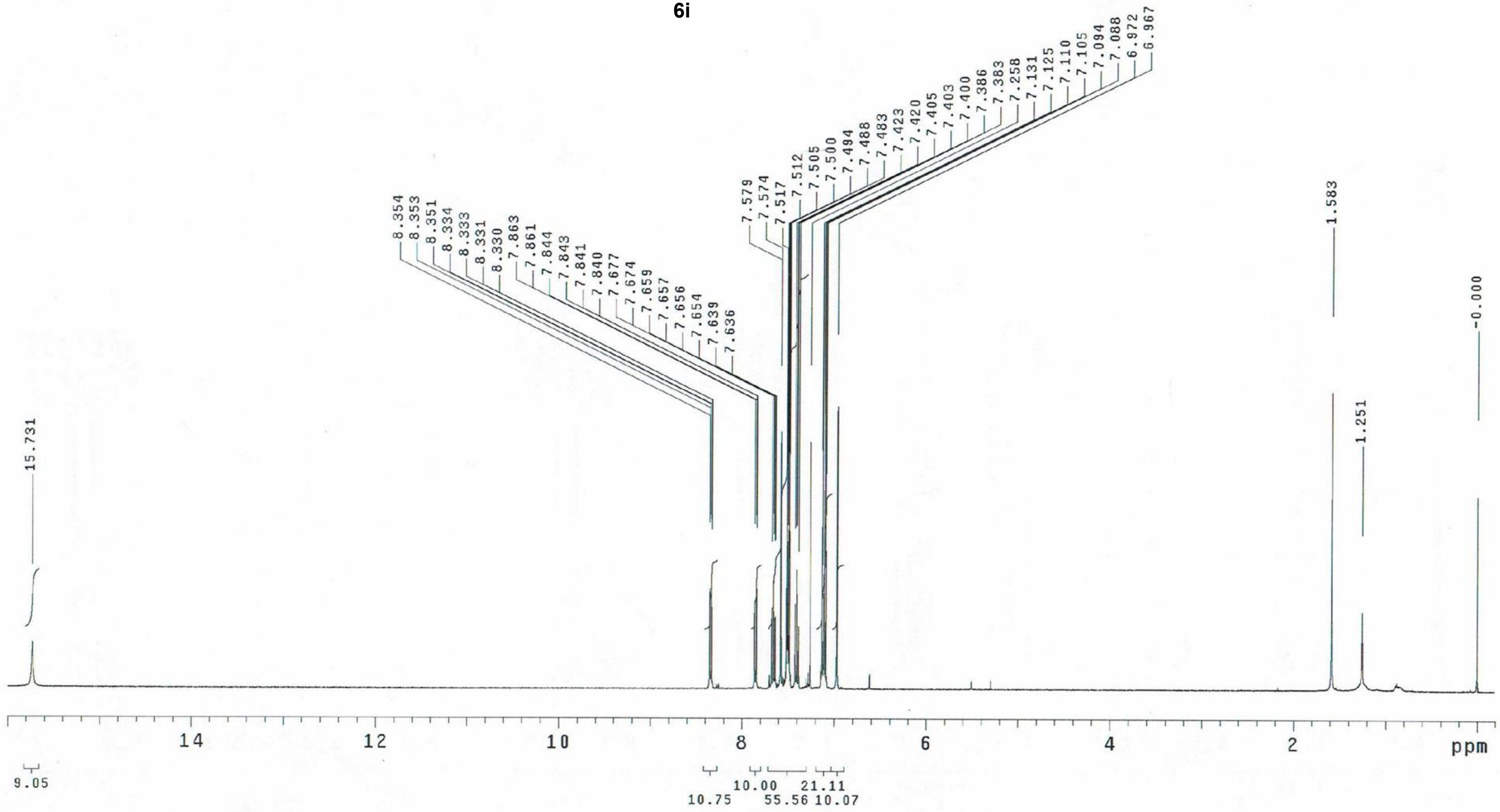

NaFu-NN4FPh

Pulse Sequence: s2pul

Solvent: CDCl3
Ambient temperature
UNITYplus-400 "unityplus400"

Pulse 65.3 degrees
Acq. time 1.000 sec
Width 25000.0 Hz
5680 repetitions
OBSERVE C13, 100.6523505 MHz
DECOUPLE H1, 400.2894267 MHz
Power 44 dB
continuously on
WALTZ-16 modulated
DATA PROCESSING
Line broadening 1.0 Hz
FT size 65536
Total time 5 hr, 36 min, 56 sec

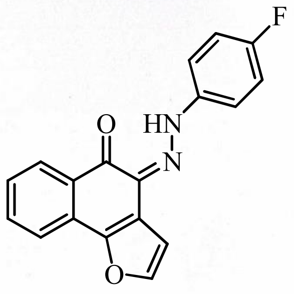

6i

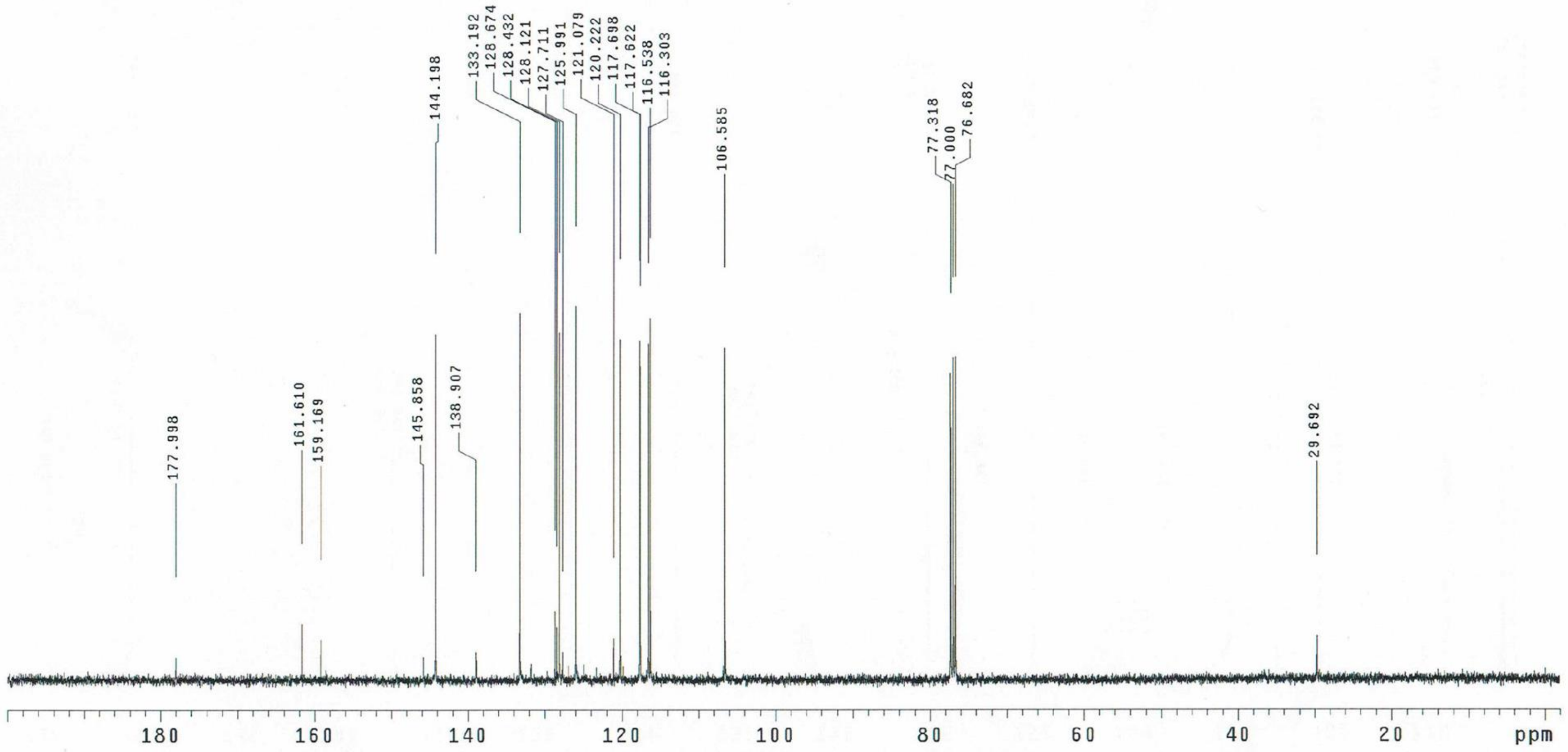

OMe-1

Pulse Sequence: s2pul
Mercury-400BB "MerPlus400"
Date: Jan 18 2018
Solvent: cdcl3
Ambient temperature
Total 32 repetitions

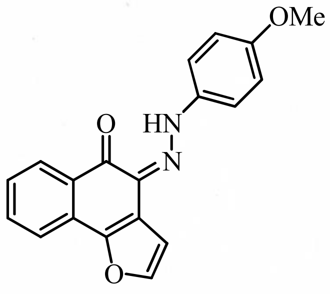

6j

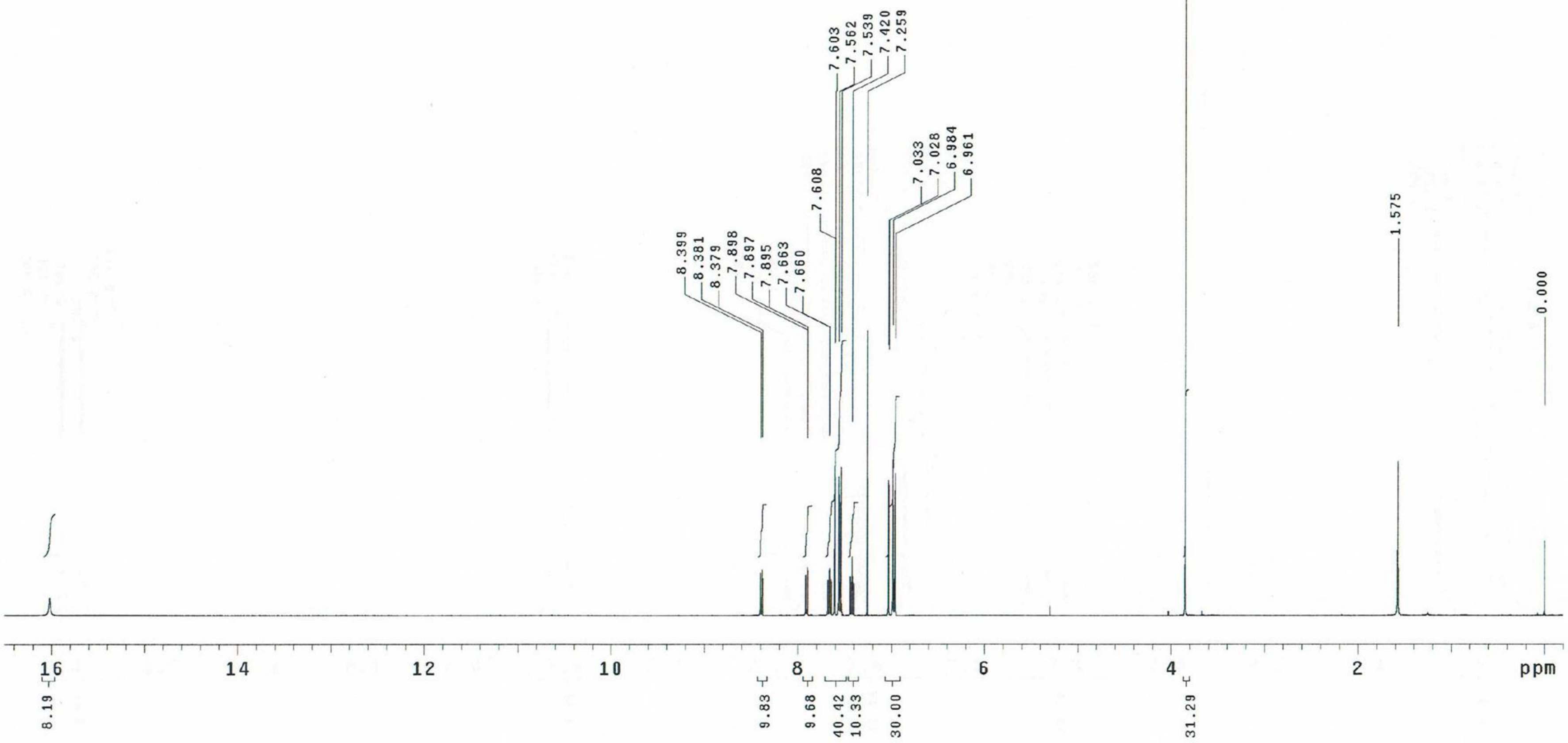

OMe-1

Pulse Sequence: s2pul
Mercury-400BB "MerPlus400"
Date: Jan 18 2018
Solvent: acetone
Ambient temperature
Total 1696 repetitions

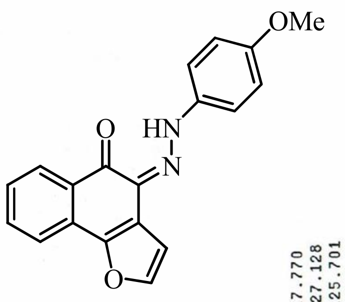

6j

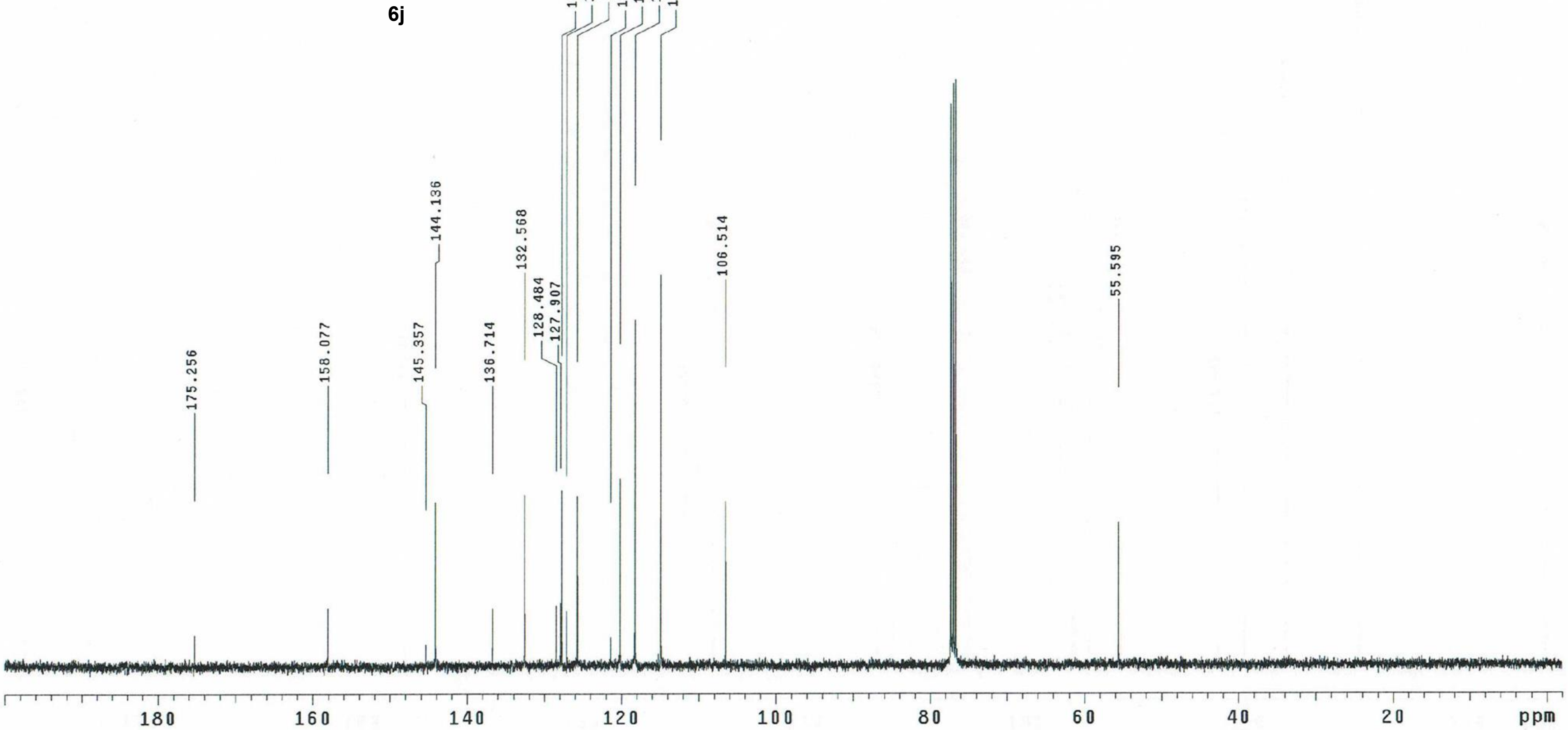

NaFu-NN4CH3Ph

Pulse Sequence: s2pul

Solvent: CDCl3
Ambient temperature
UNITYplus-400 "unityplus400"

Pulse 44.8 degrees
Acq. time 3.200 sec
Width 8000.0 Hz
36 repetitions
OBSERVE H1, 400.2874138 MHz
DATA PROCESSING
FT size 65536
Total time 2 hr, 51 min, 13 sec

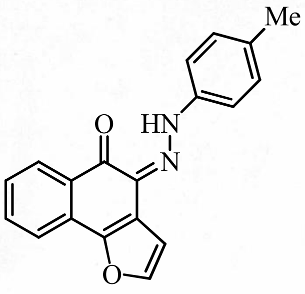

6k

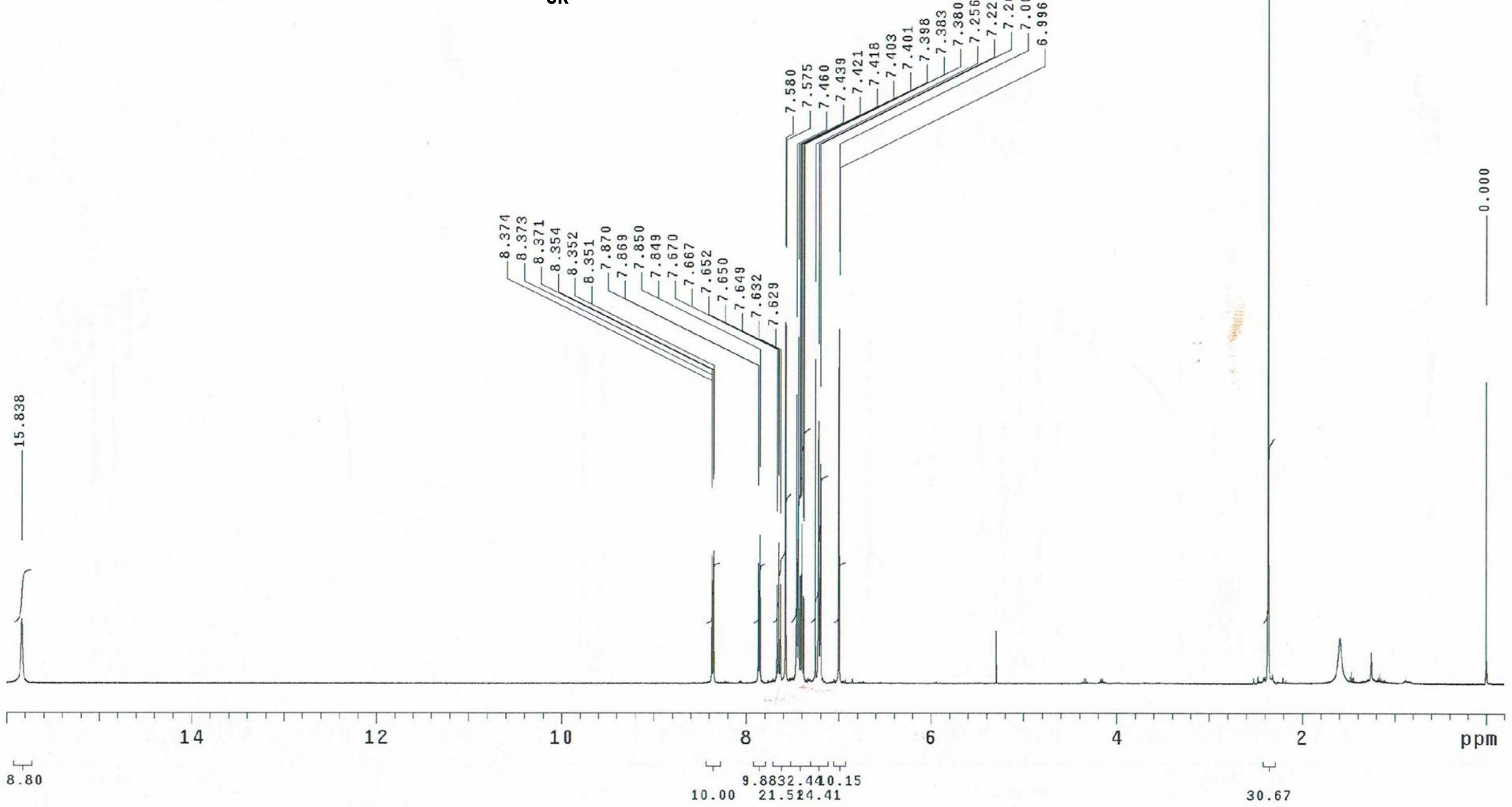

NaFu-NN4CH3Ph

Pulse Sequence: s2pul

Solvent: CDCl3
Ambient temperature
UNITYplus-400 "unityplus400"

Pulse 65.3 degrees
Acq. time 1.000 sec
Width 25000.0 Hz
2304 repetitions
OBSERVE C13, 100.6523505 MHz
DECOUPLE H1, 400.2894267 MHz
Power 44 dB
continuously on
WALTZ-16 modulated
DATA PROCESSING
Line broadening 1.0 Hz
FT size 65536
Total time 5 hr, 36 min, 56 sec

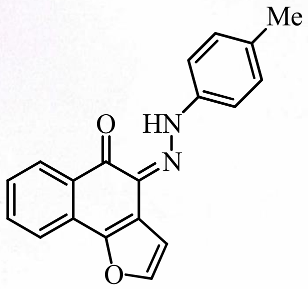

6k

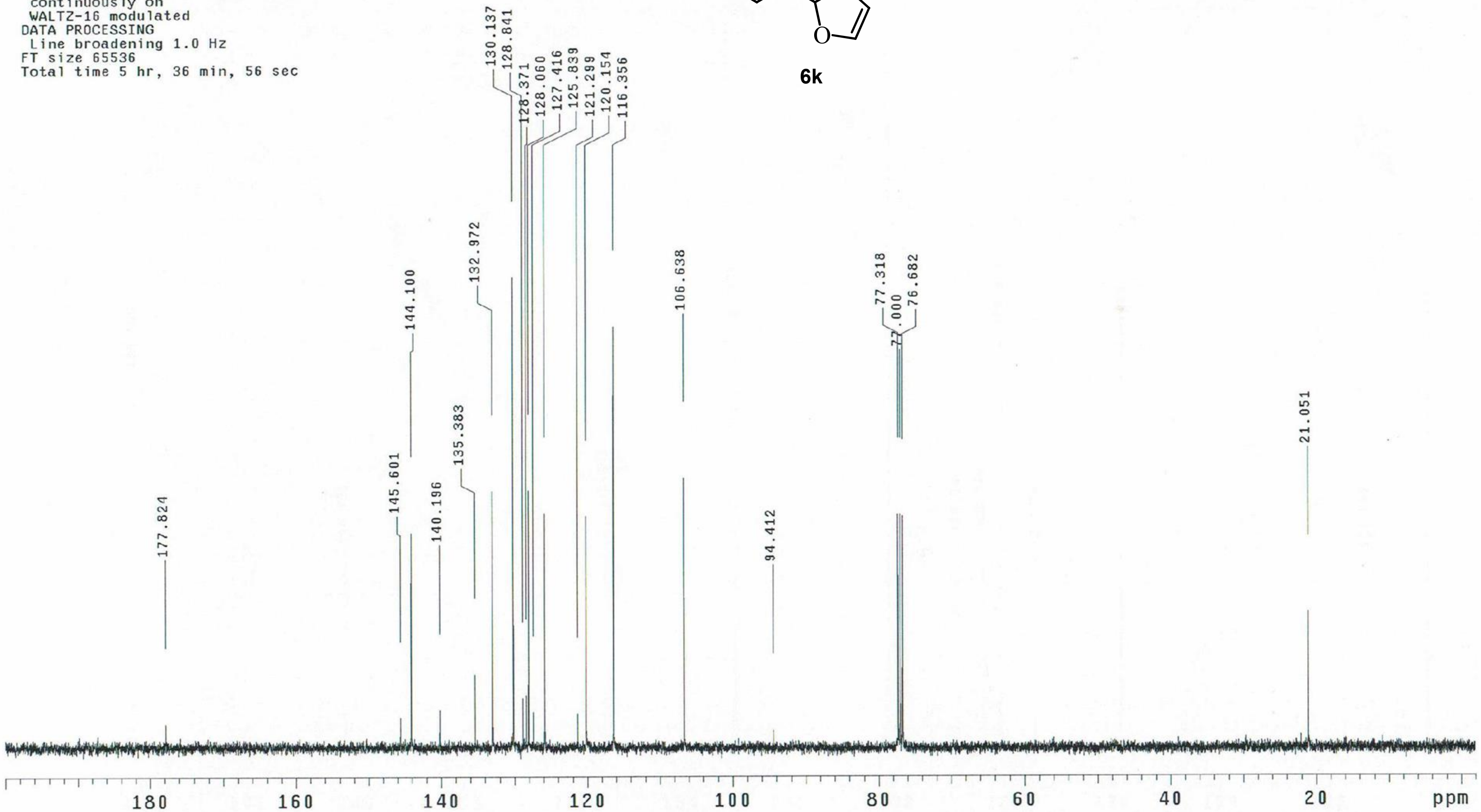

LiNaFu-MeS

Pulse Sequence: s2pul

Solvent: CDCl3
Ambient temperature
Mercury-400BB "Mercuryplus400"

Pulse 42.1 degrees
Acq. time 3.000 sec
Width 6006.0 Hz
32 repetitions
OBSERVE H1, 400.4046827 MHz
DATA PROCESSING
FT size 65536
Total time 1 min, 55 sec

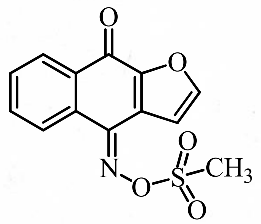

7b

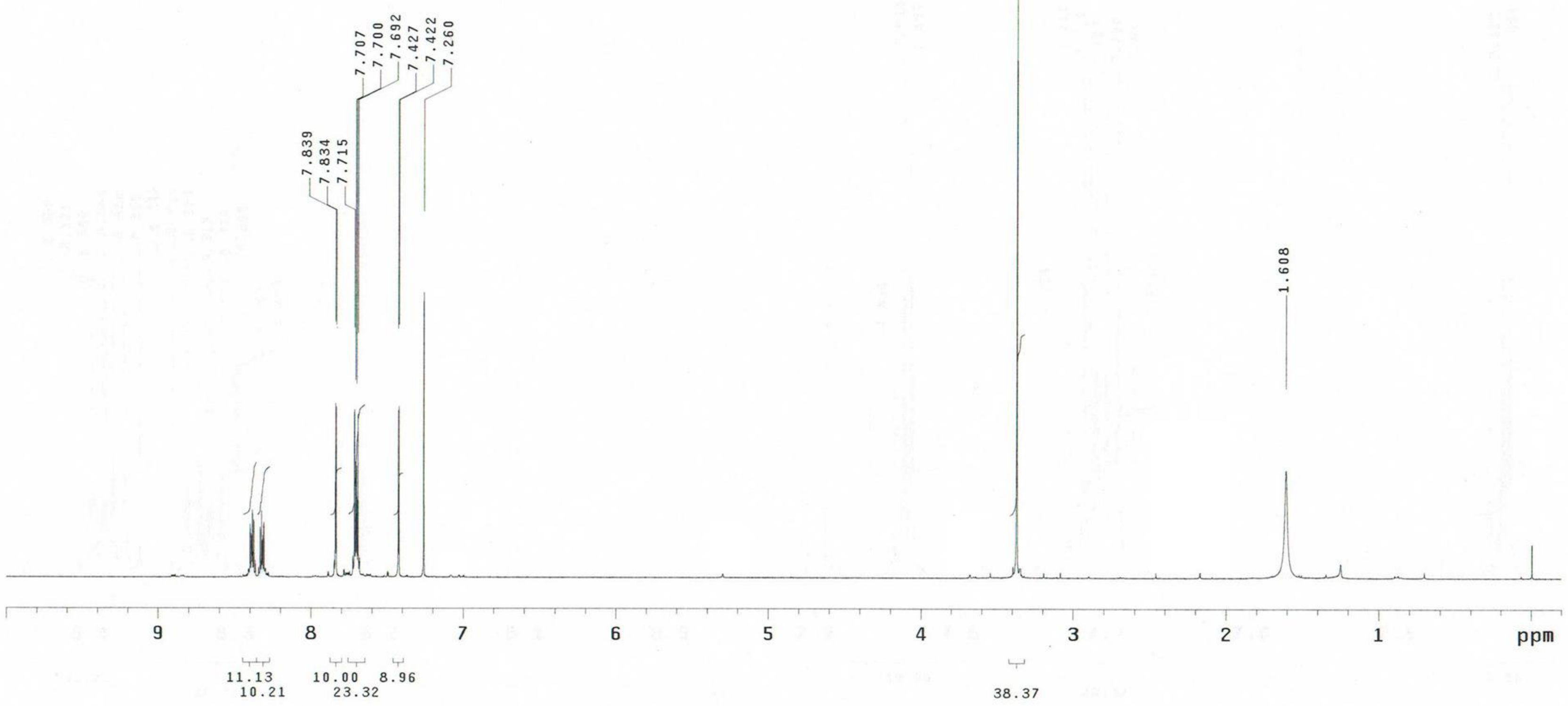

LiNaFu-MeS

Pulse Sequence: s2pul

Solvent: CDCl3
Ambient temperature
Mercury-400BB "Mercuryplus400"

Pulse 57.1 degrees
Acq. time 1.000 sec
Width 25000.0 Hz
6536 repetitions
OBSERVE C13, 100.6818389 MHz
DECOUPLE H1, 400.4066668 MHz
Power 37 dB
continuously on
WALTZ-16 modulated
DATA PROCESSING
Line broadening 1.0 Hz
FT size 65536
Total time 12 hr, 41 min, 47 sec

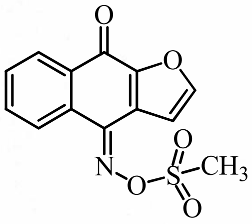

7b

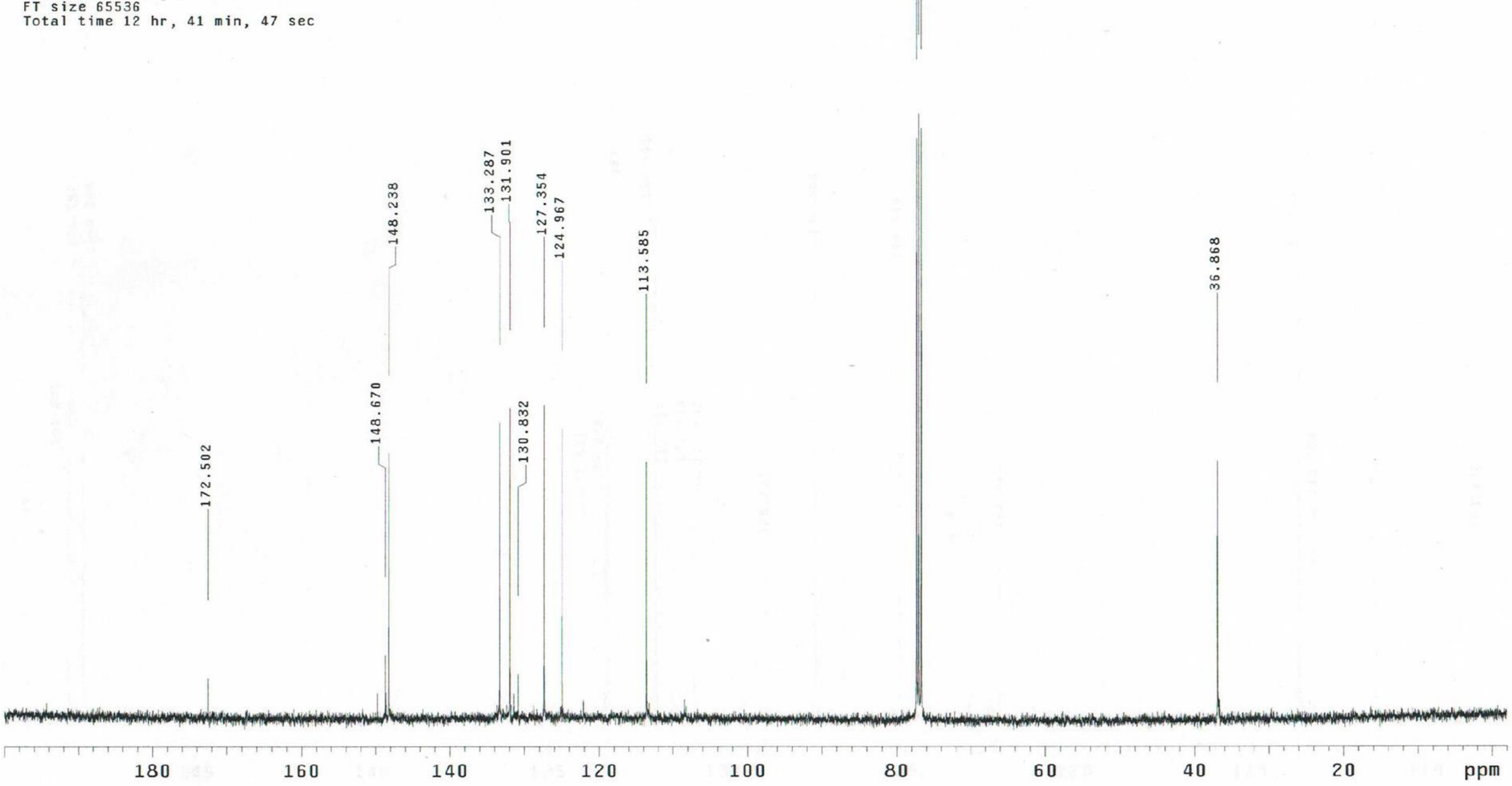

NaFu-NOAC

Pulse Sequence: s2pul

Solvent: DMSO
Ambient temperature
UNITYplus-400 "unityplus400"

Pulse 42.6 degrees
Acq. time 3.200 sec
Width 6000.6 Hz
56 repetitions
OBSERVE H1, 400.2893034 MHz
DATA PROCESSING
FT size 65536
Total time 3 min, 25 sec

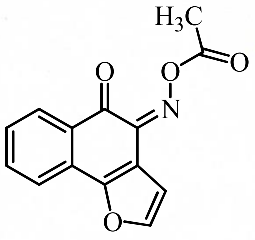

8a

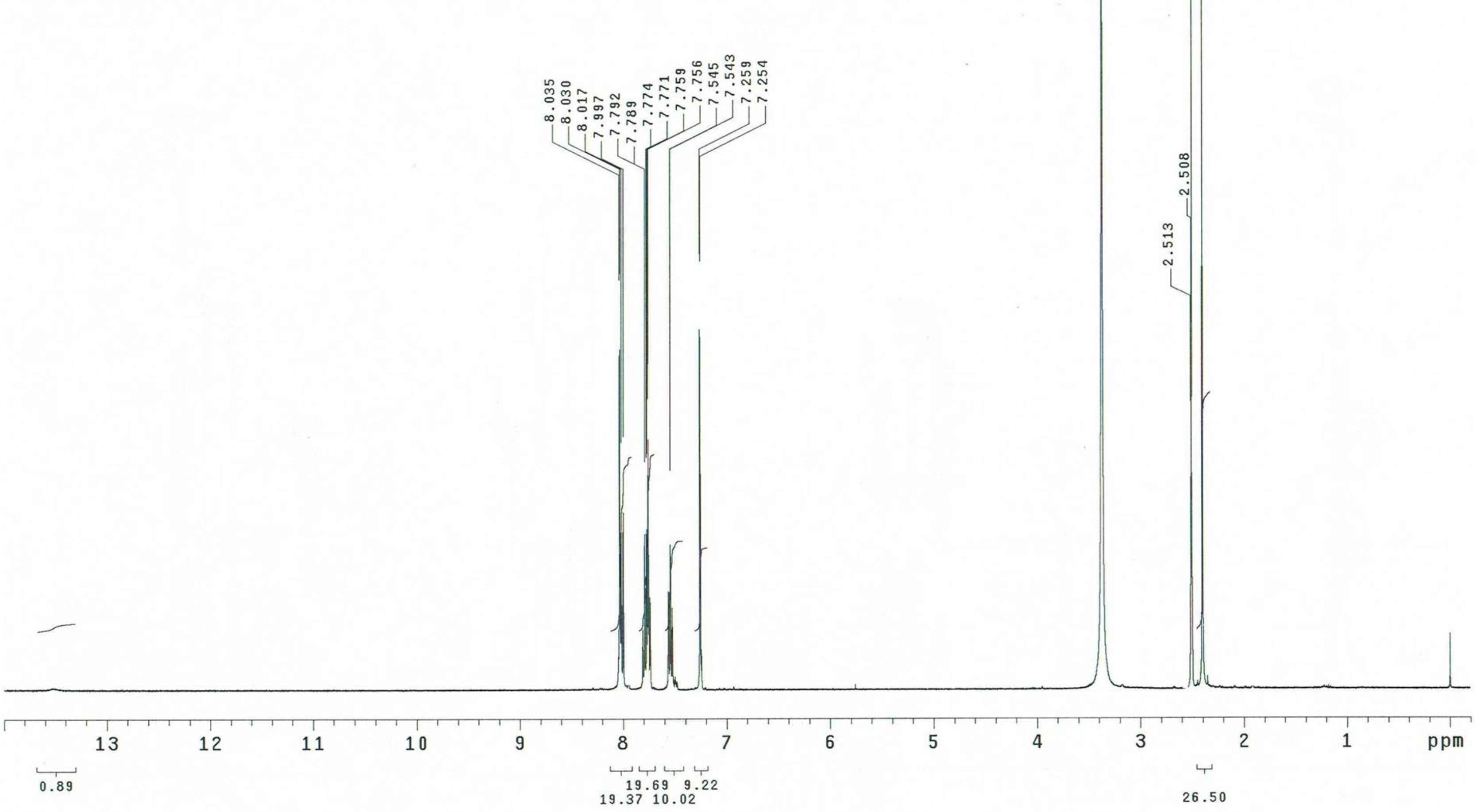

NaFu-NOAC

Pulse Sequence: s2pul

Solvent: DMSO
Ambient temperature
UNITYplus-400 "unityplus400"

Pulse 72.3 degrees
Acq. time 1.000 sec
Width 25000.0 Hz
5536 repetitions
OBSERVE C13, 100.6528737 MHz
DECOUPLE H1, 400.2913281 MHz
Power 44 dB
continuously on
WALTZ-16 modulated
DATA PROCESSING
Line broadening 1.0 Hz
FT size 65536
Total time 5 hr, 36 min, 56 sec

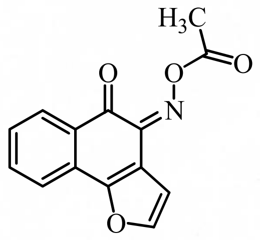

8a

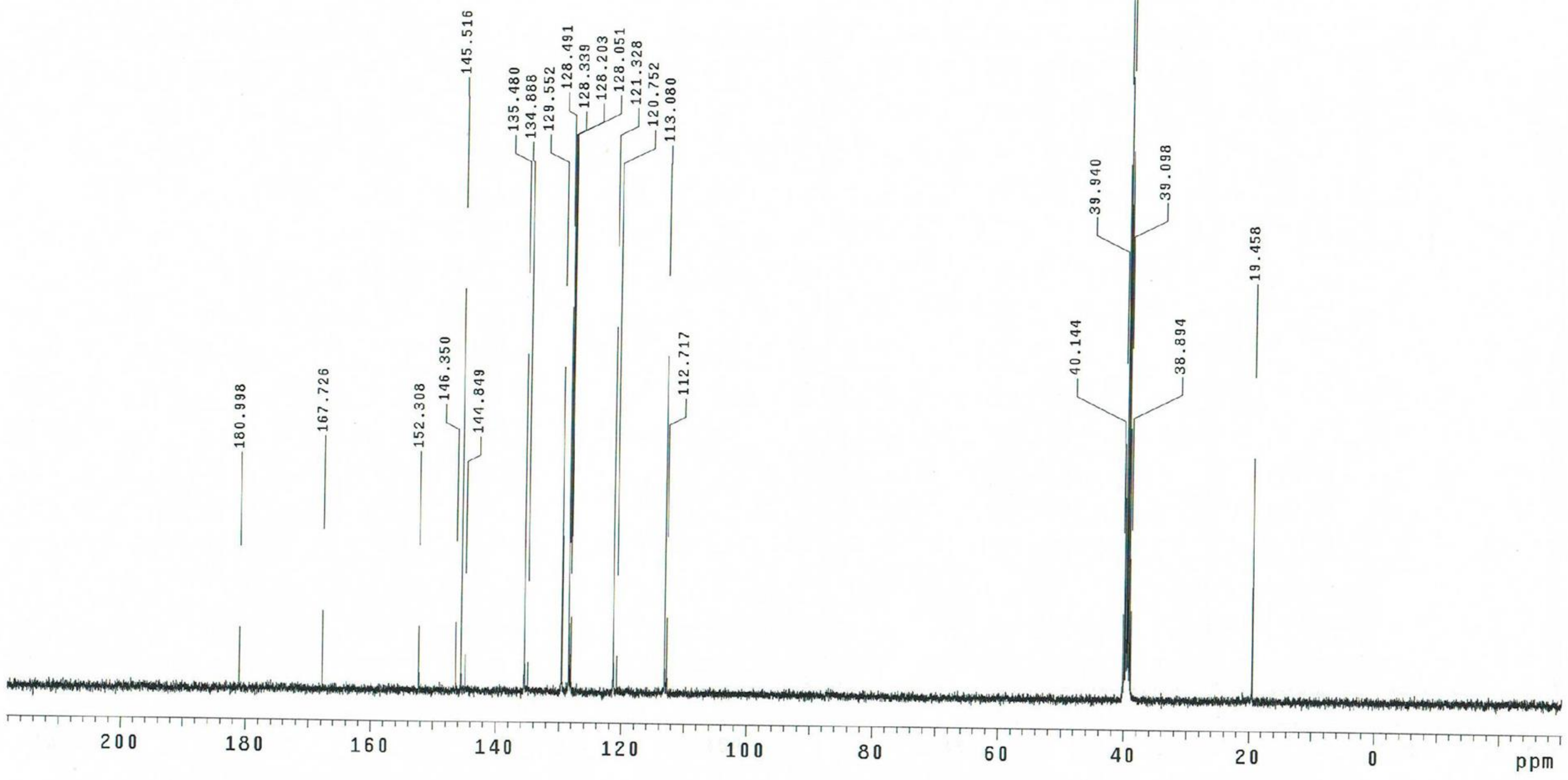

NaFu-NOS

Pulse Sequence: s2pul
Solvent: DMSO
Ambient temperature
Mercury-400BB "Mercuryplus400"

Pulse 42.1 degrees
Acq. time 3.000 sec
Width 6006.0 Hz
32 repetitions
OBSERVE H1, 400.4065764 MHz
DATA PROCESSING
Line broadening 0.1 Hz
FT size 65536
Total time 1 min, 55 sec

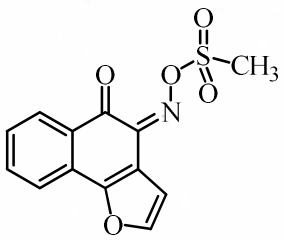

8b

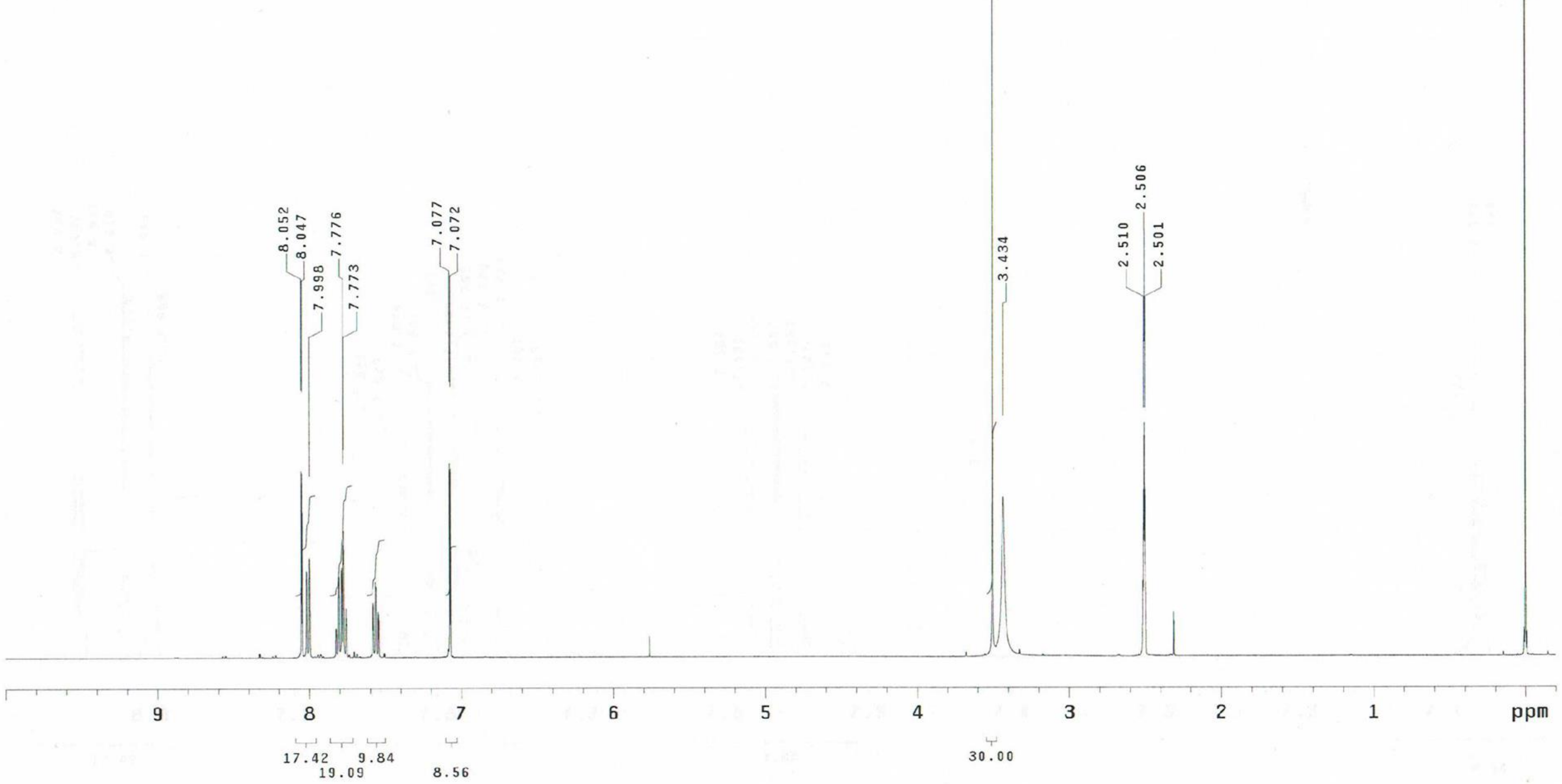

NaFu-NOS

Pulse Sequence: s2pul

Solvent: DMSO
Ambient temperature
Mercury-400BB "Mercuryplus400"

Pulse 57.1 degrees
Acq. time 1.000 sec
Width 25000.0 Hz
2256 repetitions
OBSERVE C13, 100.6823612 MHz
DECOUPLE H1, 400.4085687 MHz
Power 37 dB
continuously on
WALTZ-16 modulated
DATA PROCESSING
Line broadening 1.0 Hz
FT size 65536
Total time 12 hr, 41 min, 47 sec

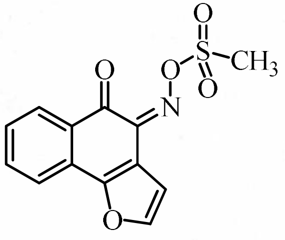

8b

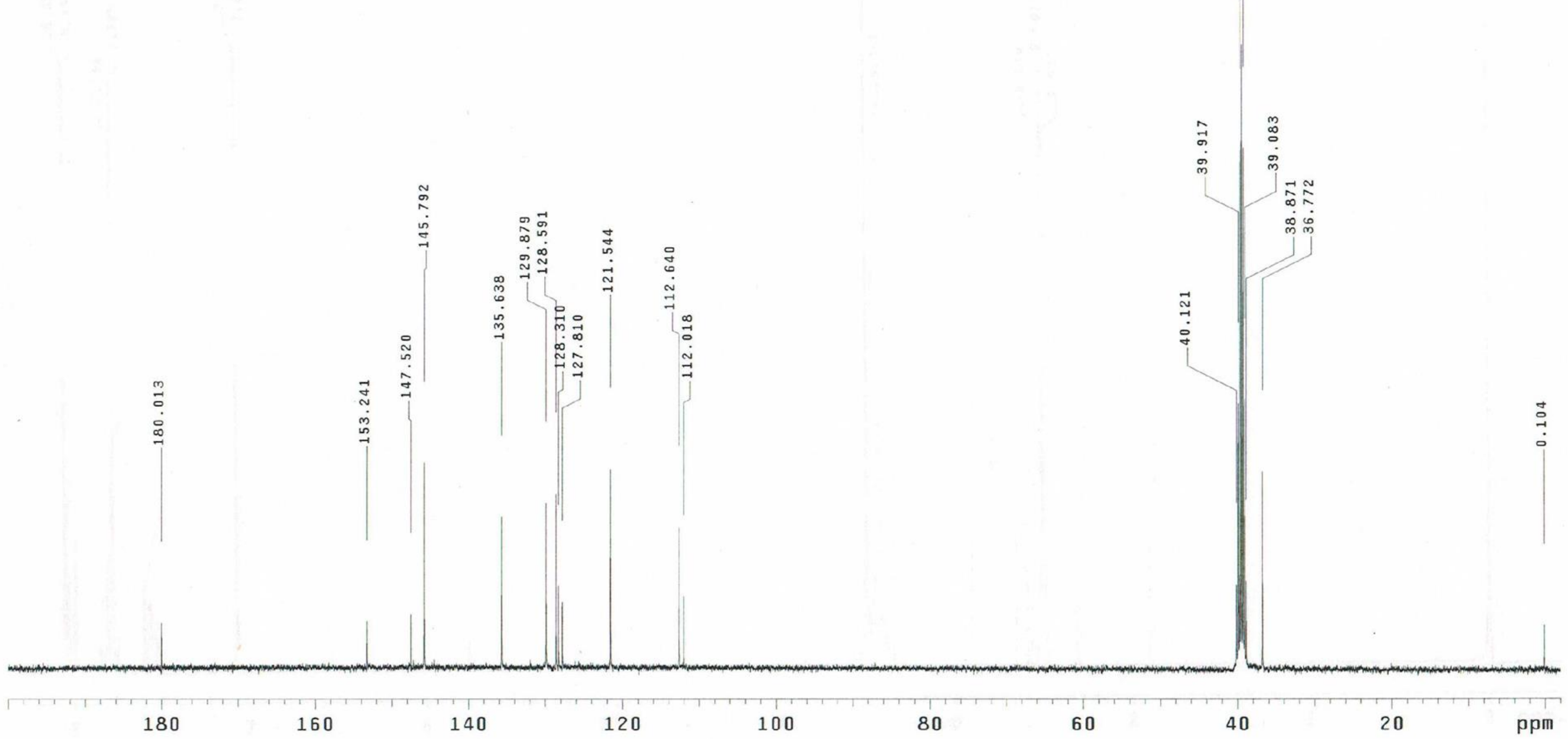

Supplement: Supplementary file 4 [file Data_Sheet_1.PDF]
